# Methods and exploratory findings of the first Swiss agricultural health cohort FarmCoSwiss

**DOI:** 10.1038/s41598-025-94440-0

**Published:** 2025-03-28

**Authors:** Julia Doetzer, Priska Ammann, Medea Imboden, Karin Ingold, Ayoung Jeong, Andrea Kaiser-Grolimund, Emmanuel Schaffner, Mirko S. Winkler, Samuel Fuhrimann, Nicole Probst-Hensch

**Affiliations:** 1 Swiss Tropical and Public Health Institute, Allschwil, Switzerland; 2 University of Basel, Basel, Switzerland; 3 Department of Environmental Social Sciences, Eawag, Swiss Federal Institute of Aquatic Science and Technology, Überlandstrasse 133, 8600 Dübendorf, Switzerland; 4 Institute of Political Science, University of Bern, Fabrikstrasse 8, 3012 Bern, Switzerland; 5 Oeschger Centre for Climate Change Research, University of Bern, Hochschulstrasse 4, 3012 Bern, Switzerland; 6 Department of Anthropology, Durham University, Dawson Building, South Road, Durham, DH1 3LE UK

**Keywords:** Agriculture, Cohort study, Occupational health, Occupational exposures, Epidemiology, Occupational health, Public health

## Abstract

FarmCoSwiss is Switzerland’s first agricultural cohort study on farmers’ health and wellbeing. It aims to longitudinally describe farmers’ mental and physical health and identify risk and protective factors. Between November 2022 and August 2023, 872 participants (65.9% men) were enrolled in the baseline survey assessing farm characteristics, occupational hazards, lifestyle and wellbeing, and physical and mental health. Selected variables were descriptively compared to the general population using Swiss Health Survey (SHS) data (2022) and the Swiss-wide SAPALDIA cohort (2020–2023). Findings suggest better physical health-related quality of life (HRQoL) in younger participants, and better mental HRQoL in older farmers. Furthermore, descriptive comparisons indicate that female farmers may have higher physical, but lower mental HRQoL than men. Most participants (60.5%) were classified as overweight or obese based on self-reported height and weight. Descriptive comparisons between the SHS and FarmCoSwiss suggest that farmers might spend less hours sitting, consume less alcohol and tobacco, but eat more red and processed meat. FarmCoSwiss participants further reported lower prevalences of most diseases compared to SAPALDIA participants. Occupational accidents were common in the farmers’ cohort. These high accident rates as well as high BMI values and indications for sex-based differences in physical and mental health highlight the need for further research and in-depth studies. Given increasing political, societal, and environmental pressures on agriculture, epidemiological evidence on farmers’ health and wellbeing is crucial to maintain a thriving agricultural workforce.

**Supplementary Information:**

The online version contains supplementary material available at 10.1038/s41598-025-94440-0.

## Introduction

In Switzerland, the agricultural sector employs roughly 150,000 people and is a crucial component of the country’s economy and cultural heritage, preserving landscapes and contributing to national food security^1^. However, the agricultural sector is considered to be one of the most dangerous occupational sectors worldwide^2^. Many work-related activities contribute to an above-average number of accidents and injuries^3^. Furthermore, farmers deal with specific occupational hazards, such as exposure to agrochemicals or physically demanding labor, that could lead to short and long-term health effects. Farmers have been recognized to be at a higher risk for respiratory diseases, certain cancer types, and neurological disorders^4–6^. For instance, the multinational BOLD study identified an association between respiratory symptoms and farming but found no association with lung function^7^.

In addition, studies show that, for example, the use of pesticides, climate change, financial difficulties, administrative burden, and loneliness can further take a toll on farmers’ mental health^8^. Largely due to high rates of farmer suicide across the globe, the issue of the impact of various factors on farmer’s mental health now seems to have gained attention in academia and among various policy actors^9^.

While risk factors are mostly in the spotlight of discussions related to farmer’s health and wellbeing, some studies have also highlighted protective factors, such as the allergy-protective effect of exposure to microbial diversity^10^. Additionally, farmers’ mortality rates have been found to be generally lower than for other occupational groups^11^. A review of studies examining the effects of different farming systems (organic vs. non-organic) on farmers’ health found that organic farmers demonstrated better overall physical and mental health indicators^12^.

Despite many studies reporting coherent findings on certain exposures and their association with health outcomes in the agricultural sector, it is important to understand complex determinant’s of farmer’s wellbeing in a particular cultural, social, ecological, and economic context. Given the challenges arising from societal changes, urbanization, digitalization, political conflicts, and climate change affecting agrifood systems, observing the health and wellbeing of farmers and their families over time is essential for maintaining a healthy and motivated agricultural workforce and for advancing sustainable food production^13,14^.

In Switzerland, little evidence is available on farmers’ overall health. The Swiss national health survey (SHS) of 2017 compared farmers with a self-employed reference population and found that the proportion of farmers who described their health as average or poor was higher than in the reference population^15^. A study published in 2020 showed that the suicide trend among male farmers is higher than that of other men in Switzerland. According to the study, the gap has widened since 2006^16^. Agricultural labor in Switzerland has also been linked to an increased risk of self-reported symptoms of chronic phlegm and chronic bronchitis compared to the general Swiss population^17^. A qualitative study interviewing Swiss dairy farmers identified health problems as one of the most frequently cited factors that negatively influence farmers’ quality of life^18^.

In order to investigate broad and longitudinal aspects of farmers’ and their partners’ health in Switzerland, the occupational cohort FarmCoSwiss (Swiss Farmer Family Health and Wellbeing Cohort) was established in 2022 in the frame of the TRAPEGO project (Evidence-based Transformation in Pesticide Governance), which investigates sustainable agricultural transformation in light of societal, economic, and environmental trade-offs in Switzerland with a focus on plant protection products (PPPs) (www.trapego.ch). In the present study, we answer two research questions: (i) Which methods were used to establish the FarmCoSwiss cohort and conduct the baseline survey? and (ii) What is the general health status of the FarmCoSwiss population at baseline, and how might this inform future research on specific health aspects within the cohort? Hence, the objective of the present study is twofold: First, to describe the procedures and methodology employed in the baseline FarmCoSwiss cohort study and second, to characterize study participants at baseline in an exploratory manner to inform and guide future research.

## Methods

### Study design

FarmCoSwiss is an epidemiological cohort study investigating farmers’ health and wellbeing as well as health risk and protective factors beyond the focus on PPPs. Between the 25th of November 2022 and the 22nd of August 2023, the newly established FarmCoSwiss prospective cohort study collected baseline data of farmers and their partners living and working in Switzerland.

### Eligibility of participants

Interested individuals were eligible to participate in the study if they resided in Switzerland, were at least 18 years old, spoke German, French or Italian fluently, and worked in Swiss agriculture at the time of recruitment. Working in agriculture was defined as being self-employed, working full-time, part-time or in seasonal employment, or working without payment on a family farm. Irrespective of their occupation, farmers’ partners were also invited to participate, as they often reside on the same premise on the farm and may, therefore, also be exposed to the agricultural-specific risk and protective factors, challenges, and benefits.

### Recruitment

In the absence of access to a national registry of farms or farmers, the recruitment strategy was based on the principle of voluntary response sampling. To counteract selection bias, representativeness was increased by targeting a broad spectrum of dissemination channels and by monitoring participation closely. For dissemination, a study leaflet in print and digital format in German (see supplement S1), French (S2) and Italian (S3) and short information texts with a link to the study’s registration page (https://www.swisstph.ch/farmcoswiss) were compiled.

The recruitment process involved extensive contact with various agricultural organizations and businesses, including cantonal (subnational unit) farmer associations, women’s agricultural associations, organic farming associations, agricultural education facilities, and private companies in the agricultural sector. The study leaflet was distributed through email newsletters, social media, agricultural newspapers, and events, reaching both male and female farmers in Switzerland with different farming (organic/non-organic) and production (crop production/animal husbandry) systems. Additionally, a total of 10,000 personalized invitation letters were distributed Swiss-wide to farmers in each canton using random sampling, based on the size of the farming population within each canton.

Interested individuals first completed a short web-based survey to assess eligibility. Eligible farmers received a postal invitation with study details, an informed consent form (ICF), and a non-participation (non-responder) form. After returning the signed ICF by postal mail, participants received an email with a personalized link to the online baseline questionnaire. A print version was available upon request.

### Sample size

For sample size calculations, a difference between 12-Item Short Form Survey (SF-12v2, included in the baseline questionnaire) scores of 5% (SD 10%) between organic and non-organic farmers was assumed, based on a two-sample means test assuming a cluster-randomized design (alpha = 0.05, power = 0.8, and roh = 0.2). The target minimal sample size of 737 farmers was exceeded as a total of 872 participants were included at baseline.

### Baseline questionnaire

Eligibility screening, registration, the onboarding process, and the online questionnaire were applied in the web-based data capture and management tool Research Electronic Data Capture (REDCap) hosted at the Swiss Tropical and Public Health Institute^19,20^.

The baseline survey focused on assessing the current physical and mental health and well-being of farmers and their partners, and consisted of the eight following domains: (i) socio-demographic information, (ii) farm management and household structure, (iii) physical and mental health-related quality of life (HRQoL), (iv) lifestyle (physical activity, diet, smoking, drinking behavior, and BMI), (v) occupational hazard exposure and perception, (vi) human flourishing, sleep, and stress, (vii) work satisfaction, and (viii) medical diagnoses.

HRQoL was assessed with the SF12v2 survey instrument by Ware, Kosinski and Keller^21^, and overall wellbeing was measured by the Secure Flourish Index (SFI) by VanderWeele^22^. Higher values in the SF-12v2 and in the SFI indicate better health and wellbeing. An occupational risk matrix was developed to measure the subjective frequency of exposure to 20 pre-selected hazards and the perception of their health hazardousness using Likert scales. The hazards were chosen based on existing literature and expert knowledge about the Swiss agricultural context. They included five domains (physical, chemical, biological, psychosocial, and environmental) with four hazards each. The baseline questionnaire in German, French, and Italian can be found in the supplement (S4–S6).

### Statistical analysis

Descriptive statistics were conducted using the statistical program R (version 4.3.2)^23^. Mean and standard deviation or median and range were used to describe continuous variables. Categorical variables were reported as counts and percentages. Some metric variables were categorized for a better overview (see chapter 2.7 and Table 1 in the appendix).

The SF-12 was calculated using the scoring software “PRO CoRE 2.2”^24^. Calculations are based on 12 different items representing eight health domains. Scale scores were weighted based on the 1990 general U.S. population and the final score was standardized to the 1998 general U.S. population. Lastly, scores were aggregated to two summary scores; the mental and physical component scores (MCS, PCS). As the age groups 15–24 and 75 + in the FarmCoSwiss cohort only included very few participants (n = 2 and n = 14, respectively), data of these age categories were excluded from age-stratified MCS and PCS calculations.

Regarding the assessment of representativeness, farm and participant characteristics were compared to publicly available data (2022) from the Swiss Federal Statistical Office. For sex, region, and farming system (organic/non-organic), variable definitions employed in the FarmCoSwiss sample aligned closely with those of the Swiss Federal Statistical Office and were, thus, directly compared. Slight discrepancies in variable definitions are shown in detail in the results section.

#### Comparison to the general population

At the time of this study, Switzerland lacks in-depth surveys and a population-based health cohort representative of the general population, rendering the comparison between different populations difficult^25^. However, we had access to data collected in two different studies that were comparable in terms of utilized questionnaire instruments, variable definitions, and time period of data collection. To examine potential differences between the farmers’ cohort and the general population, FarmCoSwiss data was descriptively compared to results of the Swiss Health Survey (SHS) of 2022 and to results from the fifth follow-up of the Swiss-wide Study on Air Pollution And Lung Disease In Adults (SAPALDIA) cohort conducted between 2020 and 2023, where possible^26,27^. The SHS is a component of Switzerland’s multi-year statistical program, with population-representative data collection occurring every five years since 1992. Publicly available SHS data from 2022 was used for the comparison of lifestyle variables (sitting time, meat, alcohol, and tobacco consumption, and BMI) between FarmCoSwiss participants and the Swiss general population. Due to very small sample sizes (n < 15) in the age groups 15–24 and 75 + in the FarmCoSwiss cohort, these age categories were excluded from age-stratified comparisons with the SHS data. Considering identical variable definitions and institutional access, data from the Swiss-wide, population-based SAPALDIA cohort was used to descriptively compare diagnosed disease prevalences assessed in both the FarmCoSwiss and SAPALDIA studies. SAPALDIA is a biobank cohort with participants recruited from eight different geographic locations in Switzerland, covering the country’s different language regions. As the cohort has been monitored since 1991, the vast majority of SAPALDIA participants in the fifth follow-up (2020–2023) were above the age of 50. Consequently, the comparison of disease prevalences was restricted to participants aged 50 and above in both the FarmCoSwiss and the SAPALDIA cohort. Detailed information on the representativeness, as well as inclusion and exclusion criteria of the SAPALDIA study can be found elsewhere^28^.

Data concerning the prevalences of (non-)occupational accidents were sourced from the Federal Statistical Office, as SAPALDIA did not include questions on accidents^29^. For the SF-12v2 and the variables physical activity, stress and sleep quality, there was no comparable data available for the general population in either the SHS or SAPALDIA. Hence, we present only FarmCoSwiss results for these variables in chapter 3 “Results” and interpret our findings with other study results in chapter 4 “Discussion”.

Based on the present study’s objectives and aims, multivariable models were not conducted and statistical significance of the explorative comparisons between FarmCoSwiss, SHS, and SAPALDIA was not assessed.

### Variable definitions

An overview of selected variables (farm, lifestyle, and disease prevalences) presented in this publication is shown in the appendix (Table A1), including variable definitions, response options, categorizations, and recoding. This section provides a summary of some variables requiring further information.

*Farming system* was categorized as either organic or non-organic. The term non-organic is used here instead of the expression “conventional” since “conventional agriculture”, as it is widely understood in international literature, does not necessarily apply to the Swiss agricultural setting. Switzerland has implemented the concept of integrated pest management (IPM). In IPM, chemical control measures are only used if preventive and non-chemical measures cannot guarantee sufficient crop protection. Hence, differences in terms of sustainable food production between organic and non-organic farms in Switzerland may not be as pronounced as in other settings.

*Physical activity* was defined as being physically active for at least 30 min, leading to breathlessness and sweating. Answers were given in days per week (0–7 days). To assess *sitting time*, participants were asked how many hours per day they usually spend sitting. Information on both variables was assessed separately for work and rest days and for the warm and cold seasons. However, as the variables were highly correlated between seasons for work and rest days (sitting hours work day: spearman r = 0.69, *p* < 0.000; sitting hours rest day: spearman r = 0.78, *p* < 0.000; physical activity work day: spearman r = 0.81, *p* < 0.000; physical activity rest day: spearman r = 0.84, *p* < 0.000), they were averaged across seasons for this publication.

Few lifestyle variables were re-coded to match the variable categorizations in the SHS. This includes *sitting time* [h/d], which was categorized into “less than 4 h”, “4 to 5 h”, “6 to 7 h”, “8 to 9 h” and “10 h or more”. Answer options in the SHS for *alcohol consumption* consisted of “less than once a week”, “1 to 2 times a week”, “3 to 6 times a week”, or “every day”. For better comparison, the SHS categories “1 to 2 times a week” and “3 to 6 times a week” were re-coded as “several times a week”. Regarding *meat consumption*, FarmCoSwiss data was categorized into “1 to 3 days/week or less”, “4 to 6 days a week” and “daily”. Notably, FarmCoSwiss participants were asked how many days per week they consume red meat, while in the SHS, participants were asked for their general meat consumption.

### Ethical aspects

In accordance with the Ordinance on Human Research with the Exception of Clinical Trials (HRO) act, all legal requirements and ethical standards were fulfilled. Informed consent was obtained from all subjects and/or their legal guardian(s) before the start of the study. This research project was performed in accordance with the current version of the Declaration of Helsinki, as well as all national legal and regulatory requirements (Human Research Act (HRA) and Human Research Ordinance (HRO)). Participants were asked to fill in a questionnaire and no biological samples were taken. Collection of personal data entailed only minimal risks and burdens (labeled as A, low risk, HRO (Art. 7)). The Ethics Committee Northwestern and Central Switzerland (EKNZ) reviewed and approved this project (BASEC-No.: 2022-00549).

## Results

### Study participants

**Fig. 1:**
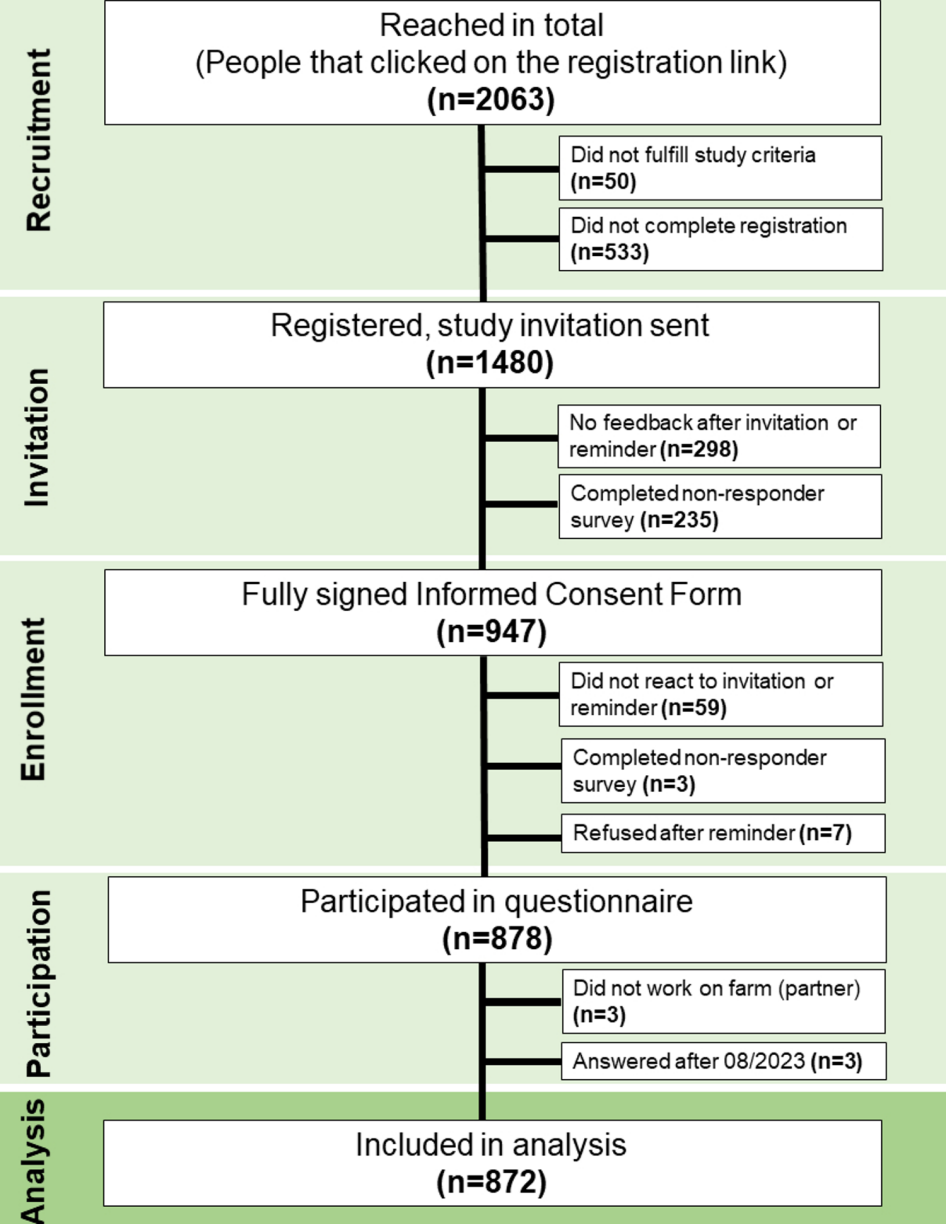
CONSORT flow chart of individuals eligible, self-registered, enrolled in the study, withdrawn from the study, and included in analysis in the FarmCoSwiss cohort.

The CONSORT flow chart gives an overview of the study participation (Fig. 1). A total of 2063 individuals were reached through our recruiting strategy. Of those, 1480 individuals were eligible, fully completed the registration form, and were formally invited via postal mail. Of all invited participants, 947 returned the signed ICF and were therefore registered for the study. A total of 59 individuals who signed the ICF did not respond to the questionnaire or subsequent reminder emails and calls, and thus did not participate in the study. Ten registered individuals withdrew participation after enrolment.

A total of 878 participants started to answer the questionnaire, and 866 fully completed it. As only three participants were partners who did not work in agriculture, they were excluded from this analysis. Since the baseline data collection was closed in August 2023, results from three individuals responding at a later stage were also excluded in the present analyses. The total sample size for analysis was therefore 872.

Of these, 159 participants completed the questionnaire on paper. The average duration for completing the online questionnaire was 36.9 min (median of 32.0 min). The actual duration may be somewhat shorter, as participants had the option of saving their responses and resuming the questionnaire at a later time point. All participants who took more than 120 min to complete the questionnaire (n = 123) were excluded from this duration calculation, since it is assumed that they took a long break.

### Non-responder

A total of 238 registered individuals indicated their intention to withdraw from the study by completing the short non-responder survey either after receiving the study information or signing the ICF. The non-responder questionnaire in German, French, and Italian can be found in the supplementary data (S7–S9). In short, the non-responder questionnaire consisted of 10 questions about reasons for non-participation, socio-demographic and farm-related characteristics and about the general health and quality of life, measured on a 5-point Likert scale. Table A2 in the appendix gives an overview of the non-responding individuals’ characteristics in comparison to FarmCoSwiss participants. In general, more men and individuals with a somewhat lower educational level and above the age of 65 were non-responders. Descriptively comparing the self-reported general health status in responders and non-responders, non-responders reported potentially poorer health. A multiple choice question was used to determine the reasons for non-participation. The most frequently selected reasons for non-participation were “no time” (46%), "too much effort" (40.1%), and "not interested or convinced of the purpose of the study" (27.8%). Other reasons were “doubts about data protection measures” (19.4%), “I refuse to participate in studies in general” (5.5%), “illness” (3.8%), “I do not fulfill the conditions for participation” (9.3%) or “other” (19.8%).

### Sociodemographic characteristics

**Fig. 2:**
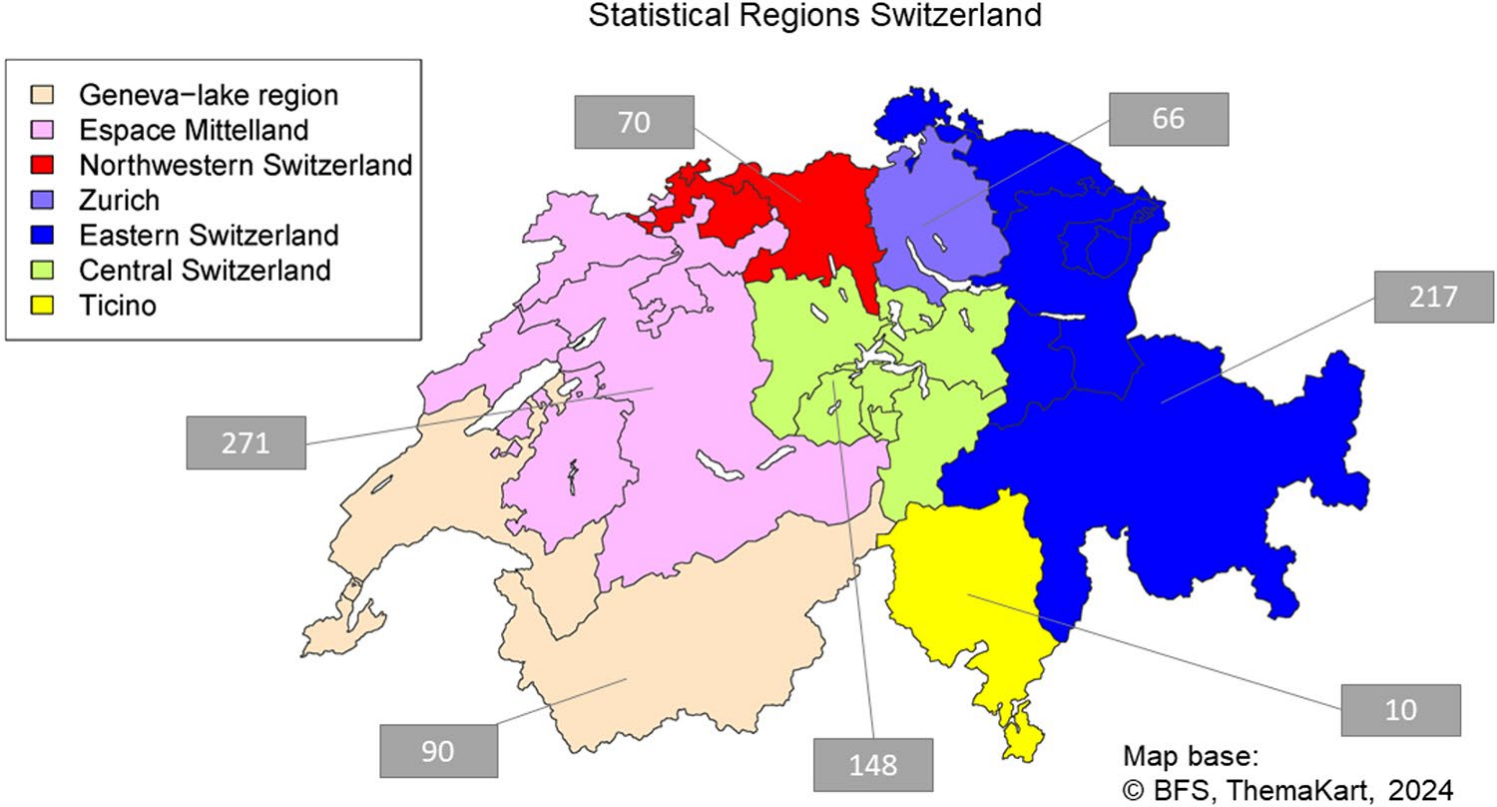
Seven statistical regions of Switzerland. Numbers in grey boxes represent the number of FarmCoSwiss participants in each region.

Table 1 describes basic socio-demographic characteristics of the 872 FarmCoSwiss participants. A total of 579 participants (65.9%) were male and only 33 participants (3.8%) had a low educational level, defined as attending only mandatory primary and secondary school (≤ 9 years). The majority of participants (81.2%) was in the age range of 35–64 years. Figure 2 depicts the geographical regions in Switzerland. Participation rate generally corresponded to region size, with most participants from Espace Mittelland (n = 271) and Eastern Switzerland (n = 217) regions.

**Table 1:** Socio-demographic characteristics of the FarmCoSwiss cohort at baseline (n = 872).

Socio-demographic characteristics	Participants (n)	Percentage (%)
**Sex**		
Male	575	65.9
Female	297	34.1
**Age**		
18–24	2	0.2
25–34	104	11.9
35–44	216	24.8
45–54	266	30.5
55–64	226	25.9
65–74	44	5.0
75 +	14	1.6
**Educational level**^**1**^		
Low	33	3.8
Middle	427	48.9
High	408	46.8
NA	4	0.5
**Region**^**2**^		
Geneva-lake region	90	10.3
Espace Mittelland	271	31.1
Northwestern Switzerland	70	8.0
Zurich	66	7.6
Eastern Switzerland	217	24.9
Central Switzerland	148	17.0
Ticino	10	1.1

^1^Education: low = mandatory primary and secondary education (≤ 9 years), middle = vocational training or high school (≤ 12 years), high = higher technical or vocational school or university (> 12 years).

^2^Analysis regions of the Swiss Federal Statistical Office^30^.

### Farm characteristics

Farm characteristics are displayed in Table 2. The vast majority of participants (89.7%) reported to work on farms that can be broadly classified as either livestock farming or mixed production, which includes the cultivation of crops in addition to livestock. A detailed overview of the types of animal farming and crop production can be found in the appendix (Figs. A1 and A2). Most farms (77.1%) were between 11 and 50 ha in size. Moreover, 77.4% of participants worked on non-organic farms. Roughly three quarters of participants (75.1%) were (co-)manager of their farm and 67.4% of participants owned the farm. Three quarters (75.2%) of participants worked full-time in agriculture.

**Table 2:** Farm characteristics of the FarmCoSwiss cohort at baseline (n = 872).

Farm characteristics		
**Production system**		
Animal husbandry^1^	782	89.7
Crop cultivation	82	9.4
NA	8	0.9
**Farming system**		
Organic	195	22.4
Non-organic	675	77.4
NA	2	0.2
**Farm owner**		
Participant	588	67.4
Family member/partner	243	27.9
Unrelated person or institution	37	4.2
NA	4	0.5
**Farm size**		
< 5 ha	29	3.3
5–10 ha	71	8.1
11–20 ha	243	27.9
21–50 ha	429	49.2
> 50 ha	100	11.5
**Work load**		
Full-time	656	75.2
Part-time	195	22.4
Other (e.g., hobby)	16	1.8
NA	5	0.6
**Job position**^**2**^		
(Co-)manager	655	75.1
Employee, family business (with income)	127	14.6
Employee, family business (without income)	110	12.6
Employed with management function (subordinate employees)	16	1.8
Employed without management function (no subordinate employees)	11	1.3
Retired with work activity	44	5.0
In training	3	0.3
Other	5	0.6
NA	7	0.8

^1^Animal husbandry encompasses mixed farms, defined as agricultural establishments with animal husbandry and crop production.

^2^Absolute shares exceed total participant number, since values are answers of a multiple choice item. Percentages are based on total participants.

To assess representativeness of the FarmCoSwiss study population, Table 3 displays selected variables in comparison to their distribution within the Swiss agricultural population based on data from the Swiss Federal Statistical Office of 2022^31^. The study sample consisted of 34.1% female participants, similar to the 36.7% women in Swiss agriculture in 2023^32^. The geographical distribution of farms in FarmCoSwiss was similar to the national distribution^33^. Organic farms (22.4%) were slightly overrepresented in our sample^34^. Farms of size < 5 ha made up 3.3% of the FarmCoSwiss sample, but 16.0% of all Swiss farms, and 49.2% of farms in our study were of size 21–50 ha compared to 37.8% with size 20– < 50 ha in Swiss agriculture^35^.

**Table 3:** Comparison of selected socio-demographic and farm characteristics between the FarmCoSwiss cohort at baseline (n = 872) and the Swiss farming population (n = 149,578) and Swiss farms (n = 48,344).

	Participants (n)	FarmCoSwiss population (%)	Swiss farming population 2022 (%)
**Sex**			
Male	575	65.9	63.3
Female	297	34.1	36.7

		FarmCoSwiss population (%)	Swiss farms 2022 (%)
**Region**			
Geneva-lake region	90	10.3	13.6
Espace Mittelland	271	31.1	32.2
Northwestern Switzerland	70	8.0	8.0
Zurich	66	7.6	6.4
Eastern Switzerland	217	24.9	21.4
Central Switzerland	148	17.0	16.4
Ticino	10	1.1	2.1
**Farming system**			
Organic	195	22.4	16.2
Non-organic	675	77.4	83.8
**Farm size**^**1**^			
< 5 ha	29	3.3	16.0
5–10 ha	71	8.1	12.3
11–20 ha	243	27.9	27.3
21–50 ha	429	49.2	37.8
> 50 ha	100	11.5	6.7

^1^The definitions of size categories vary slightly. The Swiss Federal Statistical Office categorizes farm size as follows: < 5 ha, 5– < 10 ha, 10– < 20 ha, 20– < 30 ha, 30– < 50 ha, 50 + ha^35^.

### Health-related quality of life

Mean MCS and PCS of the SF-12v2 were 47.6 (± 10.0) and 51.9 (± 7.6), respectively. With a mean of 53.1 (± 6.5), results suggest a slightly higher PCS in female participants compared to men (51.4, ± 8.0). The opposite trend was observed for the MCS, with a mean of 46.5 (± 10.0) for women and 48.2 (± 10.0) for men.

**Fig. 3:**
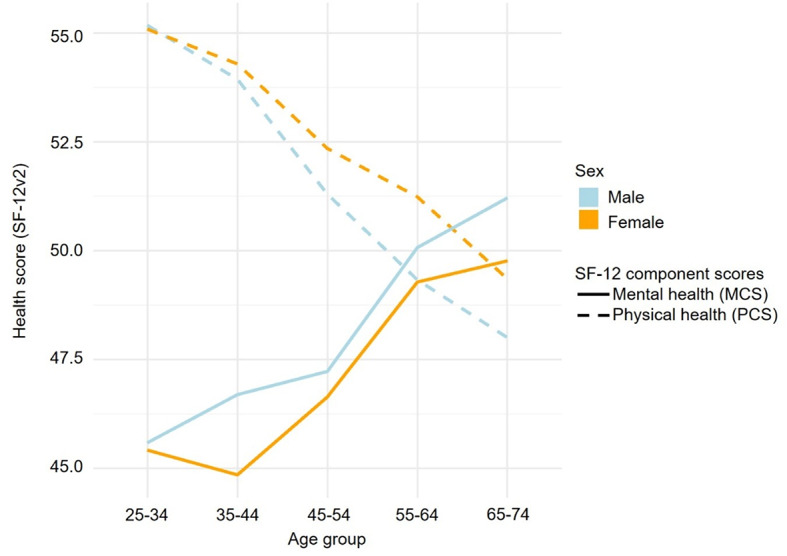
Distribution of the mental (MCS) and physical component (PCS) scores of the SF12v2 questionnaire in the FarmCoSwiss cohort, stratified by age and sex. Higher scores represent better health-related quality of life.

The distribution of the MCS and PCS according to sex and age is presented in Fig. 3. There was a general trend for increasing mental health and decreasing physical health scores with aging. At younger ages, findings suggest lower average mental health than physical health scores. Around 55–64 years of age, mental health scores exceeded physical health scores in men, whereas in women, this trend was observed at 65–74 years of age. In general, there was an indication for higher physical health, but lower mental health, scores in women compared to men.

### Lifestyle and BMI

Table 4 displays the distribution of the lifestyle variables sitting time, physical activity, diet, smoking status, alcohol consumption, and BMI, stratified by age and sex. On average, participants reported to sit more hours per day on a rest-day than on a day of work. Similarly, findings suggest that physical activity was on average higher across all sex- and age-groups on a work day compared to a rest day.

**Table 4:** Distribution of lifestyle variables in the FarmCoSwiss cohort at baseline (n = 872), overall and stratified by age and sex.

	Male, < 50 yrs	Male, ≥ 50 yrs	Female, < 50 yrs	Female, ≥ 50 yrs	Total
**Participants, n (%)**	270 (31.0)	305 (35.0)	173 (19.8)	124 (14.2)	872 (100)
	**mean (SD)**	**mean (SD)**	**mean (SD)**	**mean (SD)**	**mean (SD)**
**Sitting (hours/day)**					
Work day	3.4 (2.0)	3.7 (2.2)	3.3 (1.5)	4.1 (2.3)	3.6 (2.1)
NA	12	4	4	2	22
Rest day	3.9 (1.9)	4.4 (2.2)	4.3 (1.9)	4.9 (2.1)	4.3 (2.1)
NA	4	4	2	0	10
**Physical Activity (days/week)**					
Work day	3.8 (2.2)	3.3 (2.2)	3.3 (2.1)	2.8 (2.1)	3.4 (2.2)
NA	1	1	3	1	6
Rest day	1.1 (1.2)	1.3 (1.6)	1.9 (1.7)	1.8 (1.6)	1.4 (1.6)
NA	1	2	1	0	4
**Diet (days/week)**					
Meat	5.0 (1.7)	4.6 (1.8)	4.3 (2.0)	4.1 (1.9)	4.6 (1.9)
NA	1	2	2	0	5
Cooked vegetables	5.1 (1.6)	5.1 (1.7)	5.3 (1.7)	5.1 (1.6)	5.1 (1.7)
NA	1	1	2	2	6
Raw vegetables	5.2 (1.7)	5.4 (1.7)	5.6 (1.6)	6.0 (1.5)	5.5 (1.7)
NA	2	1	2	0	5
Fruit	4.1 (2.2)	4.5 (2.3)	5.1 (2.1)	5.4 (1.9)	4.6 (2.2)
NA	3	1	2	0	6
	n (%)	n (%)	n (%)	n (%)	n (%)
**Smoking**					
Never	147 (54.4)	189 (62.0)	119 (68.8)	104 (83.9)	559 (64.1)
Former	42 (15.6)	71 (23.3)	27 (15.6)	8 (6.5)	148 (17.0)
Smoker	79 (29.3)	42 (13.8)	26 (15.0)	11 (8.9)	158 (18.1)
NA	2 (0.7)	3 (1.0)	1 (0.6)	1 (0.8)	7 (0.8)
**Alcohol consumption**					
Never	12 (4.4)	26 (8.5)	20 (11.6)	23 (18.5)	81 (9.3)
Less than once/week	155 (57.4)	153 (50.2)	122 (70.5)	79 (63.7)	509 (58.4)
Several times/week	99 (36.7)	100 (32.7)	29 (16.8)	22 (17.7)	250 (28.7)
Daily	3 (1.1)	23 (7.5)	0 (0.0)	0 (0.0)	26 (3.0)
NA	1 (0.4)	3 (1.0)	2 (1.2)	0 (0.0)	6 (0.7)
**BMI**					
Underweight (< 18.5)	0 (0.0)	1 (0.3)	2 (1.2)	2 (1.6)	5 (0.6)
Healthy weight (18.5–24.9)	91 (33.7)	85 (27.9)	101 (58.4)	52 (41.9)	329 (37.7)
Overweight (25–29.9)	133 (49.3)	167 (54.8)	42 (24.3)	47 (37.9)	389 (44.6)
Obesity (≥ 30)	43 (15.9)	49 (16.1)	26 (15.0)	21 (16.9)	139 (15.9)
NA	3 (1.1)	3 (1.0)	2 (1.2)	2 (1.6)	10 (1.1)

An examination of dietary patterns across all age groups indicates that, on average, individuals consumed raw vegetables more frequently than fruit, meat, or cooked vegetables. Men under the age of 50 reported the highest consumption frequency of meat, with an average of 5.0 days per week, while women aged 50 and above reported the lowest consumption frequency, with an average of 4.1 days per week.

A majority of participants in each age and sex group stated to have never smoked. The rate of never smokers was similar between men younger than 50 years (54.4%) and men in the older age group (62.0%), but was reportedly higher in women ≥ 50 years (83.9%) than in younger women (68.8%). The highest share of current smokers was found in men under 50 (29.3%).

Regarding alcohol consumption, most study participants reported to drink less than once a week (58.4%). While 36.7% of men under 50 years reported to consume alcohol several times a week, the share in the same age group for women was 16.8%. A similar pattern was found for participants of age 50 and older.

Concerning BMI, the majority of participants were classified as overweight or obese (60.5%). In all age and sex groups, obesity rates were around 15%. Men under and above 50 years of age had overweight rates of 49.3% and 54.8%, respectively. With 24.3% for women under 50 years old and 37.9% for women of 50 years and older, overweight rates were reportedly lower in female participants.

### Stress and sleep quality

Table 5 describes participants’ self-reported stress level and sleep quality for both the cold and warm season. For both age and sex groups, comparative results suggest lower mean scores for perceived stress level in the cold season compared to the warm season. Stress levels were suggestively higher in women compared to men. A greater share of all participants reported very good sleep quality during the cold season compared to the warm season. Generally, most participants reported good or very good sleep quality in both seasons. However, women ≥ 50 years old more often stated to have mediocre or bad sleep quality in both seasons (cold: 43.6%, warm: 49.2%).

**Table 5:** Self-reported stress and sleep quality in the FarmCoSwiss cohort at baseline (n = 872), overall and stratified by age and sex.

	Male, < 50 yrs	Male, ≥ 50 yrs	Female, < 50 yrs	Female, ≥ 50 yrs	Total
**Participants, n (%)**	270 (31.0)	305 (35.0)	173 (19.8)	124 (14.2)	872 (100)
	**mean (SD)**	**mean (SD)**	**mean (SD)**	**mean (SD)**	**mean (SD)**
**Stress level cold season**^**1**^	3.1 (1.1)	2.6 (1.1)	3.3 (1.2)	3.1 (1.1)	3.0 (1.2)
**NA**	3	5	5	0	13
**Stress level warm season**^**1**^	3.9 (1.1)	3.3 (1.2)	4.1 (1.0)	3.7 (1.1)	3.7 (1.2)
**NA**	3	6	4	0	13
	n (%)	n (%)	n (%)	n (%)	
**Sleep quality cold season**					
Very good	73 (27.0)	64 (21.0)	41 (23.7)	26 (21.0)	204 (23.4)
Good	127 (46.3)	146 (47.9)	67 (38.7)	42 (33.9)	380 (43.6)
Mediocre	56 (20.7)	66 (21.6)	47 (27.2)	46 (37.1)	215 (24.7)
Bad	12 (4.4)	21 (6.9)	12 (6.9)	8 (6.5)	53 (6.1)
Very bad	1 (0.4)	3 (1.0)	2 (1.2)	2 (1.6)	8 (0.9)
NA	3 (1.1)	5 (1.6)	4 (2.3)	0 (0.0)	12 (1.4)
**Sleep quality warm season**					
Very good	52 (19.3)	56 (18.4)	31 (17.9)	19 (15.3)	158 (18.1)
Good	119 (44.1)	128 (42.0)	82 (47.4)	42 (33.9)	371 (42.5)
Mediocre	77 (28.5)	88 (28.9)	42 (24.3)	48 (38.7)	255 (29.2)
Bad	17 (6.3)	25 (8.2)	11 (6.4)	13 (10.5)	66 (7.6)
Very bad	2 (0.7)	3 (1.0)	3 (1.7)	2 (1.6)	10 (1.1)
NA	3 (1.1)	5 (1.6)	4 (2.3)	0 (0.0)	12 (1.4)

Results are presented for the warm and cold season separately. Stress was measured on a 6-point Likert scale, with higher values representing higher perceived stress levels.

^1^At home and at work (1 = no stress, 6 = extreme stress).

### Comparisons to Swiss general population samples

**Fig. 4:**
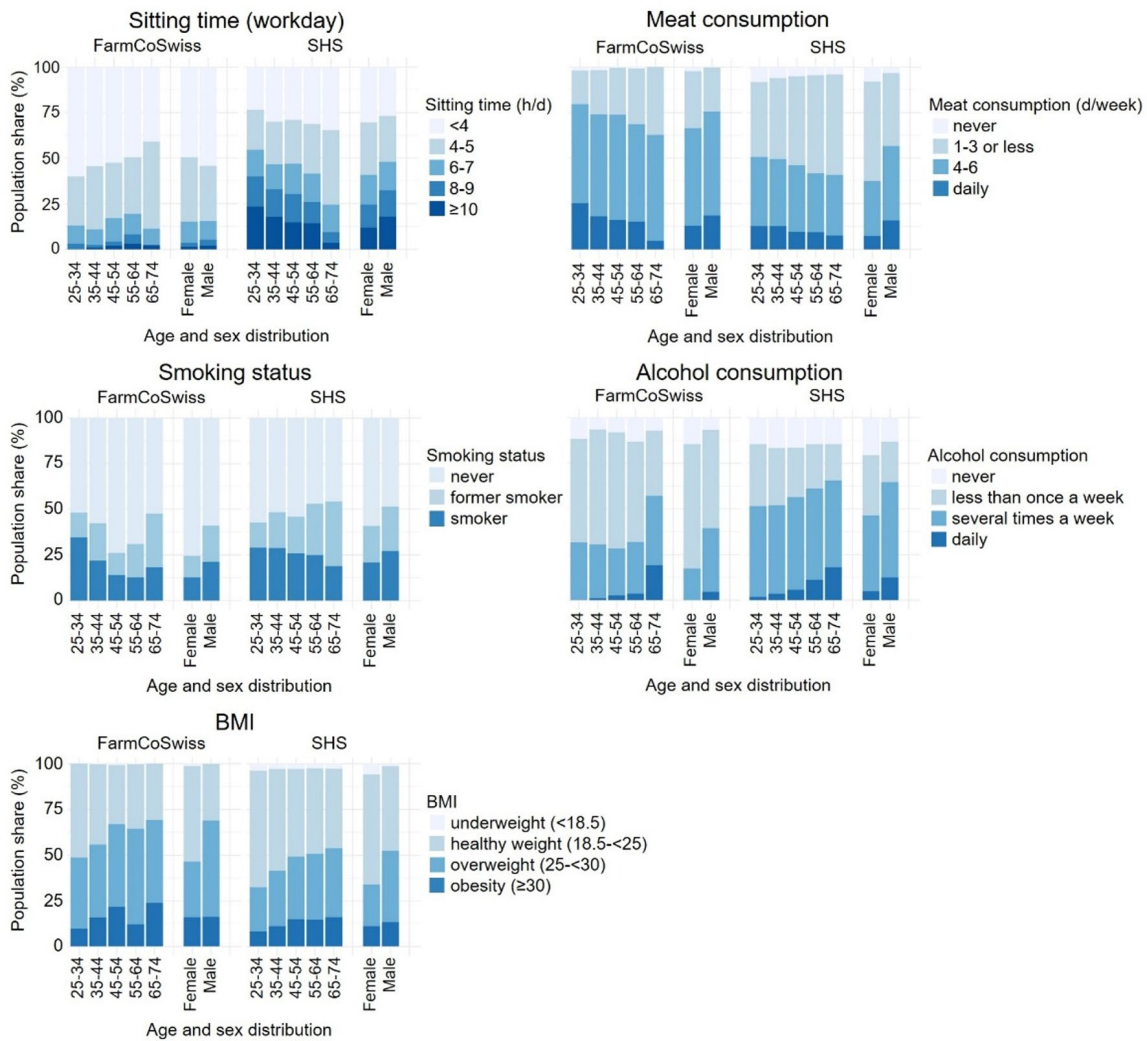
Distribution of selected lifestyle variables in the FarmCoSwiss baseline survey from 2022–2023 (n = 856) and the Swiss Health Survey from 2022 (n = 7,182,252), stratified by age and sex. From top to bottom, left to right: Frequency (%) distribution of hours spent sitting, meat consumption per week, smoking status, alcohol consumption, and BMI in the two study populations.

For a better interpretation of the baseline findings, the study population was compared to two Swiss general population samples: first, to data from the SHS 2022 for lifestyle-related variables and second, to the fifth follow-up of the SAPALDIA cohort (2020–2023) for disease lifetime prevalences. Figure 4 in chapter 3.8.1 gives an overview of the selected lifestyle variables (sitting time, meat consumption, smoking status, alcohol consumption, and BMI) descriptively compared with the general Swiss population (SHS).Tables 6 and 7 in chapter 3.8.2 present prevalences of self-reported diseases and accidents in the FarmCoSwiss and SAPALDIA cohorts.

**Table 6:** Prevalence of diagnosed diseases in the FarmCoSwiss cohort at baseline (n = 872) and in the SAPALDIA cohort (n = 2845), stratified by sex.

	FarmCoSwiss population (2022–2023)				SAPALDIA population (2020–2023)			
	Male ≥ 50 n = 305		Female ≥ 50 n = 124		Male ≥ 50 n = 1435		Female ≥ 50 n = 1359	
Diagnosed disease	Lifetime prevalence n (%^1^)	NA	Lifetime prevalence n (%^1^)	NA	Lifetime prevalence n (%^1^)	NA	Lifetime prevalence n (%^1^)	NA
Osteoarthritis or joint wear and tear	131 (43.0)	24	48 (38.7)	6	554 (38.6)	5	654 (48.1)	13
Back pain (for 3 months or longer, almost daily)	76 (24.9)	16	22 (17.7)	2	429 (29.9)	5	426 (31.3)	18
Cardiovascular diseases (heart attack, cardiac insufficiency, cardiac arrhythmia, etc.)	40 (13.1)	11	11 (8.9)	3	418 (29.1)	377	310 (22.8)	547
Allergies (hay fever, food allergy, eczema, insect venom allergy, etc.)	35 (11.5)	7	35 (28.2)	3	618 (43.1)	177	684 (50.3)	173
Depression or anxiety disorder^2^	27 (8.9)	25	22 (17.7)	8	202 (14.0)	7	299 (22.0)	9
Cancer	19 (6.2)	14	12 (9.7)	2	299 (20.8)	76	285 (21.0)	100
Asthma	15 (4.9)	19	12 (9.7)	0	170 (11.8)	10	187 (13.8)	11
Chronic bronchitis or chronic obstructive pulmonary disease (COPD)	12 (3.9)	15	1 (0.8)	2	30 (2.1)	6	29 (2.1)	11
Stroke	10 (3.3)	13	2 (1.6)	0	49 (3.4)	8	42 (3.1)	10
Diabetes or diabetes mellitus (incl. diabetes diagnosis during pregnancy)	7 (2.3)	11	5 (4.0)	1	148 (10.3)	7	66 (4.9)	12
Parkinson’s syndrome	1 (0.3)	11	0 (0.0)	2	5 (0.3)	6	6 (0.4)	15

For reasons of comparability, data is shown only for participants of age 50 and older (FarmCoSwiss: n = 429, SAPALDIA: n = 2794).

^1^Percentages were calculated exluding NAs.

^2^Includes panic attacks in SAPALDIA.

**Table 7:** Prevalence of accidents in the FarmCoSwiss cohort at baseline (n = 872) and in the Swiss Health Survey (n = 4,463,854 for occupational accidents, n = 7,182,252 for non-occupational accidents), stratified by sex.

	FarmCoSwiss population (2022–2023)				General Swiss population (SHS 2022)	
	Male 18 + n = 575		Female 18 + n = 297		Male 15 + n = 2,346,164^1^ n = 3,548,077^2^	Female 15 + n = 2,117,690^1^ n = 3,634,175^2^
	Lifetime prevalence n (%)	NA	Lifetime prevalence n (%)	NA	Prevalence n (%)	Prevalence n (%)
Accident with a visit to the doctor						
Occupational	271 (47.1)	1	84 (28.3)	0	183,001 (7.8)^3^	78,355 (3.7)^3^
Non-occupational	59 (10.3)	1	39 (13.1)	0	677,683 (19.1)^3^	537,858 (14.8)^3^

^1^Employed population ≥ 15 years 2022, used for the prevalence calculation of occupational accidents (BFS, 2024)^36^.

^2^Population ≥ 15 years in private households 2022, used for the prevalence calculation of non-occupational accidents (BFS, 2023)^37^.

^3^Federal Statistical Office data from 2022 (BFS, 2024)^29^.

#### Lifestyle variables

Roughly 50% of FarmCoSwiss participants stated to sit for less than 4 h a day. In the SHS, this proportion was only about 30% (Fig. 4). Furthermore, the proportion of FarmCoSwiss participants who reported sitting for 10 h or more a day was comparatively lower than in the SHS across all age categories and for both men and women. While comparative findings indicated a slight trend toward more hours spent sitting with older age in the FarmCoSwiss cohort, SHS data suggested the opposite trend in the general population.

While most FarmCoSwiss participants reported to eat meat 4 to 6 days a week (women: 53.6%, men: 57.2%), the percentage was comparatively lower in the SHS, with 30.0% of female and 40.9% of male participants (Fig. 4). Women less frequently stated to consume meat daily as compared to men, both in the farmers’ cohort (women: 12.9%, men: 18.3%) and the SHS (women: 7.4%, men: 15.8%). Findings further suggest lower meat consumption in older individuals in both studies.

FarmCoSwiss participants generally reported lower smoking rates compared to the SHS population (Fig. 4). While SHS data indicated slightly decreasing smoking rates with age, FarmCoSwiss participants between 55 and 64 reported the lowest smoking rates (12.6%). In both studies, the rate of current smokers was lower in women than in men (FarmCoSwiss women: 12.5%, men: 21.2%; SHS women: 20.8%, men: 27.1%).

Comparative results further indicated less frequent alcohol consumption in the FarmCoSwiss cohort as compared to the SHS. In almost all age groups, most FarmCoSwiss participants stated to drink alcohol less than once a week (Fig. 4). Only in the age group 65–74, more participants (38.1%) reported alcohol consumption several times a week or daily. In contrast, nearly 50% in most age groups of SHS stated to drink alcohol several times a week. Daily alcohol consumption was less frequent in women than in men, in both FarmCoSwiss (women: 0.0%, men: 4.6%) and SHS (women: 4.9%, men 12.4%).

While the self-reported prevalences of overweight and obesity increased with age in both FarmCoSwiss and SHS, there was a trend toward higher overweight and obesity rates in the FarmCoSwiss sample compared to the SHS (Fig. 4). In both studies, overweight prevalence was reportedly higher in men than in women (FarmCoSwiss women: 30.4%, men: 52.7%; SHS women: 22.8%, men: 39.1%).

#### Lifetime prevalences of self-reported diseases and accidents

For FarmCoSwiss participants aged ≥ 50 years, lifetime prevalences of 11 pre-selected diseases were descriptively compared to lifetime prevalences from the fifth SAPALDIA follow-up as shown in Table 6. Lifetime prevalence of occupational and non-occupational accidents in the FarmCoSwiss cohort was compared to the 1-year prevalence of accidents reported in the SHS (Table 7). All prevalences were stratified by sex.

In both cohorts, highest prevalences were reported for osteoarthritis (FarmCoSwiss: 41.7%, SAPALDIA: 43.2%), chronic back pain (FarmCoSwiss: 22.8%, SAPALDIA: 30.6%), cardiovascular diseases (FarmCoSwiss: 11.9%, SAPALDIA: 26.1%), and allergies (FarmCoSwiss: 16.3%, SAPALDIA: 46.6%). Prevalences of all diseases were higher in the SAPALDIA population than in the FarmCoSwiss population, with the exception of osteoarthritis in men (FarmCoSwiss: 43.0%, SAPALDIA: 38.6%) and COPD (FarmCoSwiss: 3.9%, SAPALDIA: 2.1%). Female FarmCoSwiss participants reported higher prevalences for allergies (men: 11.5%, women: 28.2%), depression or anxiety disorder (men: 8.9%, women: 17.7%), cancer (men: 6.2%, women: 9.7%), asthma (men: 4.9%, women: 9.7%), and diabetes (men: 2.3%, women: 4.0%). In SAPALDIA, women had higher prevalences of osteoarthritis (men: 38.6%, women: 48.1%), chronic back pain (men: 29.9%, women: 31.3%), allergies (men: 43.1%, women: 50.3%), depression or anxiety disorder (men: 14.0%, women: 22.0%), and asthma (men: 11.8%, women: 13.8%). Differences in disease prevalences between men and women for chronic back pain, allergies, cancer, asthma, COPD, stroke, and diabetes were generally larger in the FarmCoSwiss sample than in the SAPALDIA population.

The rate of occupational accidents was reportedly higher in the FarmCoSwiss cohort than in the SHS, and higher in men than in women (FarmCoSwiss men: 47.1%, women: 28.3%; SHS men: 7.8%, women: 3.7%). Non-occupational accidents were most prevalent among men in the general Swiss population (19.1%).

## Discussion

The first agricultural health cohort in Switzerland was successfully implemented between 2022 and 2023 and enrolled 872 farmers at baseline. The majority of participants (87%) agreed to be contacted for future follow-ups within the FarmCoSwiss cohort, highlighting their interest in and the need for further research on farmers’ health and wellbeing in Switzerland. Our analysis of selected lifestyle and health-related quality of life variables identified key health areas that should be prioritized in future agricultural health research.

### Participant characteristics and health-related quality of life

The majority of FarmCoSwiss participants were 45 years of age or older, and more than 30% of participants were above the age of 55. This demographic trend is evident not only in agriculture in Switzerland but also across Europe and globally, as well as in the general population. The 2020 Swiss agricultural report states that 30% of farm managers in Switzerland will reach the age limit of 65 by 2030^38^. This development has prompted the introduction of measures aimed at accelerating farm transfers. One such measure is the cessation of direct payments for individuals exceeding the age of 66^39^. Notably, the agricultural sector, with an average retirement age of 67.5, is the occupation group with the highest average retirement age in Switzerland^40^. Thus, many farmers decide to work without being eligible for direct payments anymore. In Europe, policies to support generational renewal in agriculture have been widely discussed^41^. This underscores the need for a careful consideration of the implications of an aging population on the agricultural sector. Studies conducted in low- and middle-income as well as in high-income countries have demonstrated a correlation between decreasing age and diminished work ability within the agricultural workforce^42,43^. In addition, agriculture-specific attitudes and views on farm succession and retiring might increase generational renewal difficulties in agriculture and cause transgenerational stress^44^. FarmCoSwiss provides a foundation for future research examining these patterns and their connection to farmers’ health and well-being within the Swiss context.

While farm transfers may increase stress in both older and younger farmers, we identified a trend toward better physical HRQoL in younger FarmCoSwiss participants, and better mental HRQoL in older farmers. The same trend was found in a representative, Swiss-wide study providing normative SF-36v2 data for the general Swiss population^45^. Given the high correlation between the SF-12 and SF-36 mental and physical component scores, our results indicate that physical and mental HRQoL trends may be similar in farmers and the Swiss general population^21^. Our results are also in line with European studies finding decreasing PCS and increasing MCS trends with age in agricultural communities and in the general population^46,47^.

Our descriptive results further suggest similar physical HRQoL of male FarmCoSwiss participants compared to men of the Swiss general population^45^. In women of the Swiss general population, physical HRQoL was slightly lower than in men. In contrast, our findings indicate an opposite trend toward better physical HRQoL in female than in male farmers, especially with older age. Studies conducted in European agricultural settings generally found higher MCS and PCS scores for men^46,47^. As only leisure time, but not occupational, physical activity has been found to be beneficial for cardiovascular health, our contrasting findings may be partially explained by a reportedly higher leisure time physical activity in female farmers as compared to male farmers^48^.

Concerning mental HRQoL, men reported higher scores than women in both populations^45^. European studies found agricultural workers among the occupational groups with the lowest mental and physical HRQoL and found higher MCS and PCS scores for men^46,49^. While these sex or gender differences could be attributed to different health self-assessments or interpretations of the survey questions, they could also point toward different stressors and risk factors among men and women within the agricultural setting that warrant further research^46^.

### Disease prevalences and lifestyle variables

The lower MCS observed in younger farmers and, particularly, in older women underline our findings regarding the prevalence of depression and anxiety disorders. While depression and anxiety rates in the farming population were lower than in the SAPALDIA cohort, epidemiological studies have identified higher rates of suicide and burnout among agricultural workers in Switzerland^16,50^. Since both study populations reported only diagnosed depression and anxiety, true prevalence may be higher. The lower prevalences observed in the FarmCoSwiss cohort may also suggest that mental health remains a taboo in farming communities, potentially delaying help-seeking behavior. However, further research is needed to explore undiagnosed mental disorders and symptoms in men and women, as well as barriers to accessing psychological support to better understand and improve farmers’ mental health in the Swiss context. Key risk factors for farmers’ mental health include stress, sleep quality, social isolation, institutional pressure, financial insecurity, and environmental changes^51–55^. Many of these risk factors were captured in the FarmCoSwiss baseline study and offer the opportunity to further investigate associations. However, since both risk and protective factors shape mental health, future studies should also explore preventive aspects of agricultural work in Switzerland and the relation between physical and mental health^54^.

Regarding physical health, descriptive comparisons revealed higher prevalences of the 11 examined diseases in SAPALDIA as compared to FarmCoSwiss, but also showed a particularly high prevalence of occupational accidents in the farming population. The body of scientific literature confirms the agricultural sector as one of the most dangerous professions in Europe and globally^3,56^. Despite agricultural accident prevention efforts, there were 4,243 registered accidents (12.6% of all insured farm workers in full-employment) in Swiss agriculture in 2021^57^. However, there is no central statistic on occupational accidents and farmers are not obligated to report accidents^58^. Hence, non-fatal accidents in particular are likely to be highly under-reported. Contrasting these consistent findings on elevated risks for injuries and accidents in agriculture, research regarding general morbidity and mortality in farming populations is conflicting. Our descriptive results suggest higher diseases prevalences in the general population. This aligns with other studies reporting lower overall morbidity and mortality for farmers in high-income countries compared to the general population^59–61^. However, studies comparing farming and working non-farming populations showed higher overall morbidity and mortality rates for farmers^62,63^. For specific illnesses and diseases, morbidity and mortality were found to differ between non-farming and farming populations. For example, musculoskeletal disorder and certain cancer sites were found to be more prevalent in farmers^60,62^. For cardiovascular and respiratory disorders, however, some studies report a lower risk for farmers^60,61^.

Regarding sex differences, our study suggests potentially lower prevalence rates for female farmers for six diseases, but higher prevalences for the remaining five illnesses (asthma, allergies, depression, diabetes, cancer) as compared to male farmers. Compared to women of the general population, female farmers reported lower disease prevalences in our sample. Global research on women’s health in agriculture is relatively scarce^64^. Some evidence indicates generally lower morbidity and mortality rates for female farmers as compared to male farmers^62,64^.

Often, these sex and occupational health differences are attributed to exposure and lifestyle variables. Our descriptive findings indicate higher occupational physical activity and lower tobacco and alcohol consumption rates among farmers as compared to the Swiss general population. Despite fewer reported sitting hours, self-reported overweight and obesity rates in the FarmCoSwiss cohort exceeded those of the general Swiss population. Both global agricultural studies and reports from the Swiss Federal Office for Agriculture confirm that farmers tend to have higher BMIs, despite the generally physically demanding work^15,65^. The shift from manual to mechanized work may partly explain these findings^66^. Long working hours and limited free time may also contribute to unhealthy eating and reduced vigorous physical activity^67^. Notably, occupational physical activity has been found to have detrimental effects on cardiovascular health and be inversely correlated with leisure time physical activity^48^. Moreover, research in Austria suggests a poorer diet among farmers^67^. Similarly, red meat consumption is supposedly higher in our cohort than in the Swiss general population.

Our results further suggest lower smoking rates and alcohol consumption in our cohort than in the general population. This finding is consistent with other studies reporting healthier lifestyles among farmers and lower prevalences of related health outcomes, such as lung cancer^5,68^. In contrast, some studies report the highest tobacco and alcohol consumption among agricultural workers as compared to other manual professions^59^. These findings highlight the need for country- and context-specific research on agricultural health. Therefore, future studies should investigate how physical activity and diet relate to overweight and obesity rates among men and women in Swiss agriculture. In addition, objective measurements, such as detailed dietary assessments and body composition measurements, can aid in overcoming self-report bias and other study limitations.

### Strengths and limitations

Maintaining a physically and mentally healthy agricultural workforce is crucial for labor rights, food security, and environmental conservation, all vital to future population health and wellbeing^1,69^. While global evidence on farmers’ health is valuable to identify major health trends and challenges, it is only of limited transferability to individual countries. Thus, as the first agricultural health cohort in Switzerland, the FarmCoSwiss cohort adds valuable evidence to epidemiological research on the health of agricultural workers in a small, high-income country. The proximity between (semi-)urban and rural infrastructures, the robust representation of farmers in the Swiss political system, and democratic instruments allowing the Swiss population to exercise influence on farmers’ occupational environment, render agricultural research in Switzerland particularly complex^70^. Consequently, the FarmCoSwiss cohort study presents a valuable opportunity to expand the existing body of literature on agricultural health and to contextualize findings within the Swiss-specific context.

Despite initial recruitment challenges, we exceeded the target sample size and included participants from across Switzerland and various farming contexts. The cohort enables the longitudinal study of health trends and allows for time-resolved analyses of prolonged exposure effects, strengthening causal links. Additionally, FarmCoSwiss offers the opportunity to explore new research areas, such as emerging infectious diseases related to climate change, ‘One Health’ approaches exploring human and animal health, or qualitative studies to better understand farmers’ use of health protection measures and strategies^71,72^. Regardless of these valuable opportunities gained through the establishment of the FarmCoSwiss cohort, some limitations need to be addressed.

First, it is important to note that the sample is not entirely representative of the agricultural population in Switzerland (e.g., regarding farm size). Due to the lack of access to a national registry, we could not recruit participants in a population-based manner and, therefore, selection bias is of concern. Although achieving representativeness during the establishment of a national cohort is not a priority, we made all feasible efforts to recruit a diverse study sample that is as comparable as possible to the Swiss farming population^25^. In addition, we were able to maximize retention rate, which is crucial for the setup of a new cohort. However, as is the case in many occupational cohort studies, we have to consider a potential healthy worker effect that needs to be considered in future analyses. Descriptive comparisons between study participants and non-responders reveal that self-reported general health was somewhat lower in non-responders. In addition, non-responders seem to be of higher age, male, and with a lower education status. Moreover, the majority of study participants were farm (co-)managers or family employees. In Switzerland, however, roughly 20% of the agricultural workforce are external (non-family) employees^38^. Hence, this group is clearly underrepresented in our population sample. While we do not necessarily observe a surprisingly low percentage of individuals with a low education in our cohort study, the high percentage of farm managers suggests a likely bias toward higher education. All these factors may have biased our results toward a healthier study population, both in terms of lifestyle and disease prevalences. Moreover, given that the most commonly cited reasons for non-participation were a lack of time, too much effort, or a lack of interest, it is essential to acknowledge a bias toward participants interested in health research and willing to allocate time for the study.

Second, we explicitly refrained from presenting inferential results, as the scope of the present study was to show explorative results of the FarmCoSwiss baseline survey and to inform and guide future research using FarmCoSwiss data. Therefore, comparisons between FarmCoSwiss, SHS, and SAPALDIA data need to be interpreted with sufficient caution. In addition, the youngest (18–24) and oldest (≥ 74) age groups were not comparable to the SHS due to very small sample sizes in the FarmCoSwiss study. Furthermore, questions in FarmCoSwiss and the comparative surveys did not always match entirely. For instance, FarmCoSwiss assessed red meat intake, whereas SHS assessed general meat intake; accidents were assessed as lifetime prevalence in FarmCoSwiss, but as 1-year prevalence in the SHS; mental disorders were assessed as anxiety or depression in FarmCoSwiss, but as depression, anxiety or panic attacks in SAPALDIA. Furthermore, SAPALDIA cohort participants have been followed up for more than 30 years. Thus, this cohort population is no longer a truly population-representative sample. Additionally, due to the older age in the SAPALDIA cohort, comparisons could only be made for FarmCoSwiss participants of age 50 and older. Notwithstanding the restriction to participants aged 50 and above in the comparative analyses, the SAPALDIA comparison group remains considerably older (67.7 ± 10.1) than the FarmCoSwiss group (58.3 ± 6.8), which could potentially explain the higher disease prevalences in SAPALDIA. Yet, the SAPALDIA cohort offers the best comparability concerning disease prevalences due to almost identical disease and prevalence definitions. In addition, the SAPALDIA population’s long-term follow-up for several decades implies healthy survivor bias. Thus, descriptive comparisons between the two cohort populations reasonably indicate potentially lower disease prevalences in the FarmCoSwiss study.

Third, while this study ensures linguistic and cultural accuracy through a rigorous translation process, formal validity and reliability testing were not conducted. However, the translation underwent thorough review by native speakers fluent in both the source and target languages. These individuals provided feedback to ensure conceptual equivalence and clarity. As subtle nuances or context-specific interpretations may still exist, the questionnaire’s comparability across languages may be affected.

Fourth, weighting coefficients for the SF12v2 MCS and PCS calculations for the Swiss general population were not available at the time of data analysis for the present study. Normative data for the SF36v2 in Switzerland from 2019 indicates that the Swiss population reports higher PCS and lower MCS than the U.S. population^45^. However, our findings regarding the observed trends of MCS and PCS with regard to sex and age were generally consistent with the findings of the SF36v2 study in Switzerland^45^.

Lastly, all measurements obtained and presented in the present study are self-reported and cross-sectional. Future follow-up studies may include objective measurements of, e.g., sleep quality and physical activity will add evidence to the temporal course of health outcomes and associated protective and risk factors^73^.

## Conclusion

This descriptive overview of the FarmCoSwiss baseline data suggests that in Swiss agriculture, physical and mental HRQoL may change with age and differ between men and women. Our findings further indicate that male farmers may experience poorer physical health more often than female farmers, and women in agriculture might face more mental health challenges. While prevalences of most investigated diseases were lower in our farming sample than in the general Swiss population, we identified high rates of occupational accidents and overweight and obesity in the FarmCoSwiss population that are of concern to public health. This calls for future research investigating sex- and gender-specific risk factors, health behavior, and acceptability and effectiveness of different health prevention efforts in Swiss agriculture. FarmCoSwiss can serve as a role model for setting up additional occupational cohorts in Switzerland to longitudinally examine workers’ health and wellbeing, as access to population health data in Switzerland remains challenging owing to the absence of comprehensive health and occupational registries.

## Electronic supplementary material

Below is the link to the electronic supplementary material.

Supplementary Material 1

Supplementary Material 2

## Data Availability

The data that support the findings of this study are not openly available due to reasons of sensitivity and are available from the corresponding author upon reasonable request, and under a data use agreement only. Data are located in controlled access data storage at the Swiss Tropical- and Public-Health Institute.
