# Supplementary material for: Methods and exploratory findings of the first Swiss agricultural health cohort FarmCoSwiss
Source: Sci Rep. 2025 Mar 28;15:10690. doi: 10.1038/s41598-025-94440-0 (PMC11953423; doi:10.1038/s41598-025-94440-0)
Supplement: Supplementary file 1 — Supplementary Material 1 [file 41598_2025_94440_MOESM1_ESM.docx]

# **7 Appendix**

**Table A1** FarmCoSwiss baseline questionnaire: definitions and categorizations of selected variables included in the present analyses

| **Variable name** | **Variable definition** | **Response options** | **Categorization** | **Re-coding*** |
| --- | --- | --- | --- | --- |
| **Production system** | production system employed on participant’s farm | arable farming, forestry, horticulture, grazing livestock (dairy), grazing livestock (meat), pig husbandry, poultry, special crops (e.g. wine, fruit, vegetables, berries), other (multiple choice) | Animal husbandry (with or without crops), crop production | - |
| **Farming system** | farming system employed on participant’s farm | conventional, integrated production, organic, transformation to organic, other (multiple choice) | organic or non-organic | - |
| **SF-12 physical and mental component scores (PCS, MCS)** | physical health score, mental health score | 12-item instrument with different Likert scales | scale scores aggregated into PCS and MCS | - |
| **Physical activity** | physical activity for 30+ minutes, leading to breathlessness and sweating | days per week (0-7 days),  separately for cold and warm season | days per week, averaged across cold and warm season | - |
| **Sitting time** | hours/day spent sitting, separately for work and rest day | h/d (text field) | h/d spent sitting | less than 4 hours, 4 to 5 hours, 6 to 7 hours, 8 to 9 hours, 10 hours or more |
| **Diet** | days per week with consumption of (i) red meat/sausage, (ii) cooked vegetables, (iii) raw vegetables/salad, and (iv) fruit | Likert scale: 1 (never), 2 (less than once a week) and 3 (once a week) to 9 (7 days a week) | Likert scale value (1-9) | meat consumption:  1 to 3 days/week or less,  4 to 6 days a week, daily |
| **Smoking status** | consumption of cigarettes, e-cigarettes and other tobacco products | never, not anymore, some days, daily | non-smokers, former smokers, smokers | - |
| **Alcohol consumption** | consumption of beer/wine/cider and hard liquor | Likert scale:1 (never), 2 (1-4 times a month or less), 3 (several times a week), to 4 (daily). | never, less than once a week, several times a week, daily | SHS data: 1 to 2 times a week and 3 to 6 times a week were re-coded as several times a week |
| **BMI** | calculated based on self-reported height and weight | height in cm (text field), weight in kg (text field) | underweight (<18.5), healthy weight (18.5-24.9),  overweight (25-29.9),  obesity (≥30) | - |
| **Stress level** | stress level at work or at home during past 12 months, for cold and warm season | Likert scale : 1 (no stress) to 6 (extreme stress), separately for cold and warm season | Likert scale value (1-6) | - |
| **Sleep quality** | sleep quality during past 12 months, for cold and warm season | Likert scale : 1 (very good) to 5 (very bad), separately for cold and warm season | Likert scale value (1-5) | - |
| **Lifetime prevalence**  **of 11 diseases and accidents** | medically diagnosed disease (ever, up until time of study participation) | yes/no | yes/no | - |

* Re-coding necessary for comparative analyses with SHS data.

**Table A2** Characteristics of individuals not participating in the FarmCoSwiss cohort at baseline but answering the non-responder questionnaire (n = 238; three of those completed the non-responder survey, i.e. withdrew from the study, after having signed the ICF).

|  | | **Non-responder**  **individuals (n)** | **Percentage (%)** | **FarmCoSwiss participants (%)** |
| --- | --- | --- | --- | --- |
| Sex | |  |  |  |
|  | Male | 179 | 75.2 | 63.3 |
|  | Female | 44 | 18.5 | 36.7 |
|  | NA | 15 | 6.3 | 0.0 |
| Age | |  |  |  |
|  | 18-34 | 23 | 9.7 | 12.2 |
|  | 35-44 | 44 | 18.5 | 24.8 |
|  | 45-54 | 54 | 22.7 | 30.5 |
|  | 55-64 | 63 | 26.5 | 25.9 |
|  | 65+ | 30 | 12.6 | 6.7 |
|  | NA | 24 | 10.1 | 0.0 |
| Educational level^1^ | |  |  |  |
|  | Low | 17 | 7.1 | 3.8 |
|  | Middle | 106 | 44.5 | 49.0 |
|  | High | 101 | 42.4 | 46.8 |
|  | NA | 14 | 5.9 | 0.5 |
| Farm owner | |  |  |  |
|  | Themselves | 164 | 68.9 | 67.4 |
|  | Family member/partner | 41 | 17.2 | 27.9 |
|  | Owner outside of family | 9 | 3.8 | 4.2 |
|  | NA | 24 | 10.1 | 0.5 |
| Farm orientation | |  |  |  |
|  | Crop production | 30 | 12.6 | 9.4 |
|  | Animal husbandry | 181 | 76.1 | 89.7 |
|  | NA | 27 | 11.3 |  |
| Farming system | | | |  |
|  | Organic | 42 | 17.6 | 22.4 |
|  | Non-organic | 164 | 68.9 | 77.4 |
|  | NA | 32 | 13.4 | 0.9 |
| General health | |  |  |  |
|  | Excellent | 14 | 5.9 | 6.4 |
|  | Very good | 58 | 24.4 | 30.5 |
|  | Good | 103 | 43.3 | 48.2 |
|  | Less good | 42 | 17.6 | 12.5 |
|  | Poor | 9 | 3.8 | 2.2 |
|  | NA | 12 | 5.0 | 0.2 |

^1^Education: Low = Mandatory primary and secondary education (≤9 years), Middle = Vocational training or high school (≤12 years), High = Higher technical or vocational school or university (>12 years).


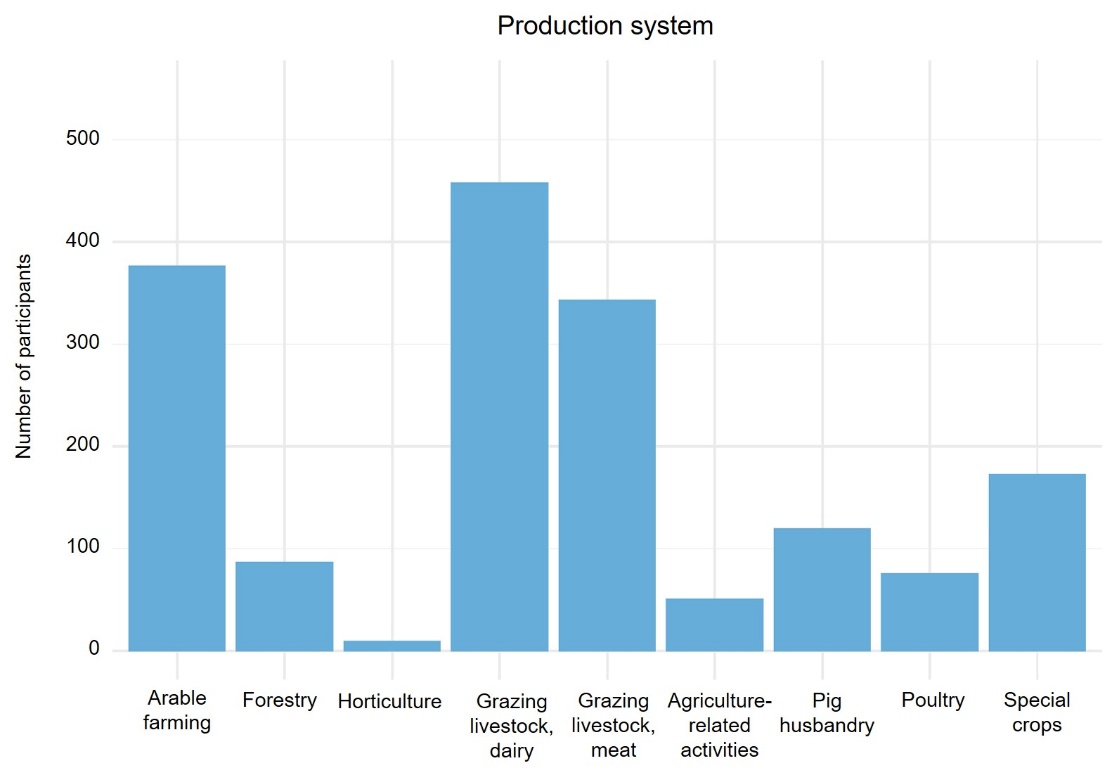


**Figure A1** Distribution of different production systems in the FarmCoSwiss (n = 872, NA = 8) cohort before categorization. Numbers may add up to more than the total sample size due to multiple choice answer options.


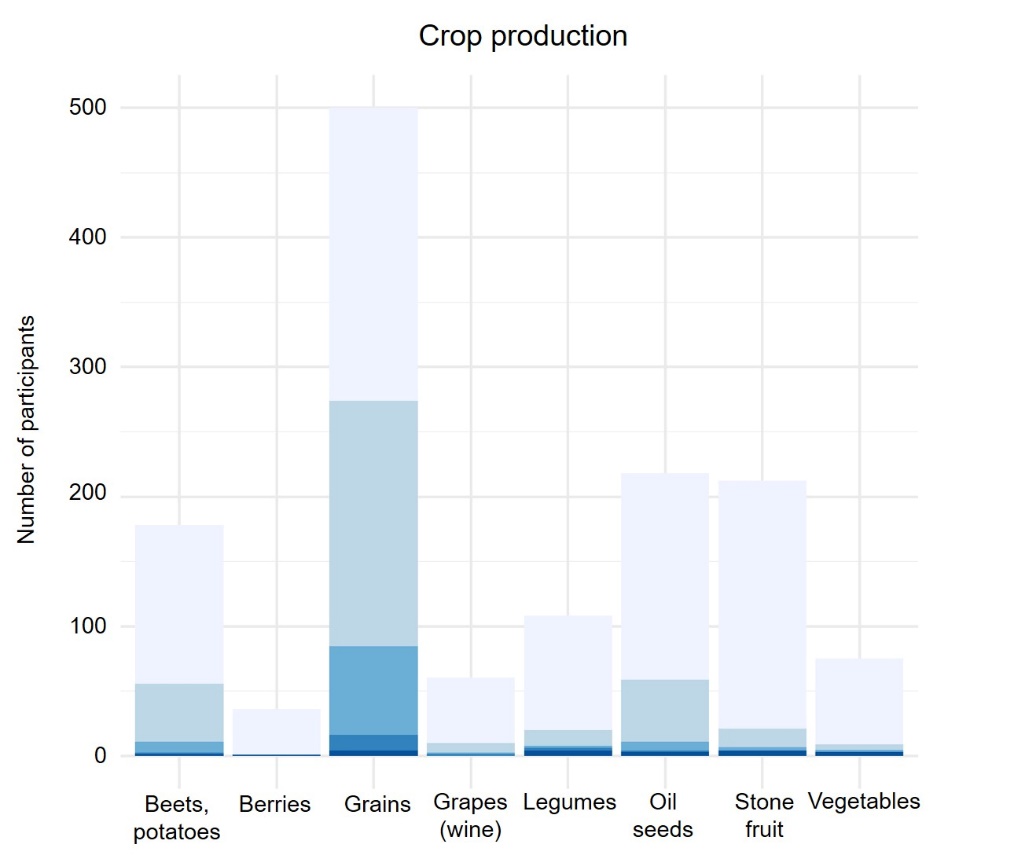


**Figure A2** Distribution of different crops in the FarmCoSwiss cohort of participants with crop production with or without animal husbandry (n = 492). Numbers may add up to more than the total sample size due to multiple choice answer options.
