# Supplementary material for: Methods and exploratory findings of the first Swiss agricultural health cohort FarmCoSwiss
Source: Sci Rep. 2025 Mar 28;15:10690. doi: 10.1038/s41598-025-94440-0 (PMC11953423; doi:10.1038/s41598-025-94440-0)
Supplement: Supplementary file 2 — Supplementary Material 2 [file 41598_2025_94440_MOESM2_ESM.pdf]

Methods and exploratory findings of the first Swiss agricultural health cohort *FarmCoSwiss*

Julia Doetzer[1,2,*], Priska Ammann[1,2,*,***] , Medea Imboden[1,2], Karin Ingold[3,4,5], Ayoung Jeong[1,2], Andrea Kaiser-Grolimund[1,2,6], Emmanuel Schaffner[1,2], Mirko S. Winkler[1,2], Samuel Fuhrimann[1,2,**], Nicole Probst-Hensch[1,2,**,***]

[1] Swiss Tropical and Public Health Institute, Allschwil, Switzerland

[2] University of Basel, Basel, Switzerland

[3] Department of Environmental Social Sciences, Eawag, Swiss Federal Institute of Aquatic Science and Technology, Überlandstrasse 133, 8600, Dübendorf, Switzerland

[4] Institute of Political Science, University of Bern, Fabrikstrasse 8, 3012, Bern, Switzerland

[5] Oeschger Centre for Climate Change Research, University of Bern, Hochschulstrasse 4, 3012, Bern, Switzerland

[6] Department of Anthropology, Durham University, Dawson Building, South Road, Durham, DH1 3LE, UK

* Shared first co-authorship

** Shared senior authorship

*** Corresponding authors

Supplement

Contents

S1 Study flyer in German 3
S2 Study flyer in French 4
S3 Study flyer in Italian 5
S4 Questionnaire in German 6
S5 Questionnaire in French 40
S6 Questionnaire in Italian 74
S7 Non-responder questionnaire in German 108
S8 Non-responder questionnaire in French 113
S9 Non-responder questionnaire in Italian 118

S1 Study flyer in German

Information und Kontakt:

www.swisstph.ch/farmcoswiss
farmcoswiss@swisstph.ch
061 284 89 29

FarmCoSwiss

Schweizerischer Nationalfonds

Das Projekt wird vom Schweizerischen Nationalfonds (SNF) Nr. CRSII5_193762 gefördert.

FarmCoSwiss

Swiss TPH
Swiss Tropical and Public Health Institute
Schweizerisches Tropen- und Public Health-Institut
Institut Tropical et de Santé Publique Suisse

Assoziiertes Institut der Universität Basel

WIE STEHT ES UM DIE GESUNDHEIT VON PERSONEN UND DEREN PARTNER/INNEN IN DER SCHWEIZER LANDWIRTSCHAFT?

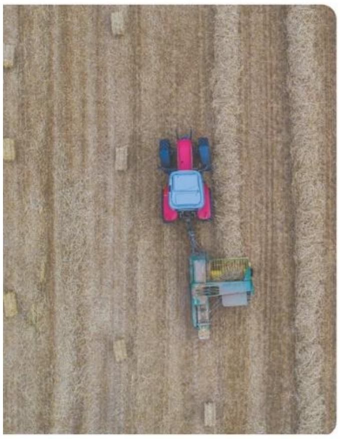

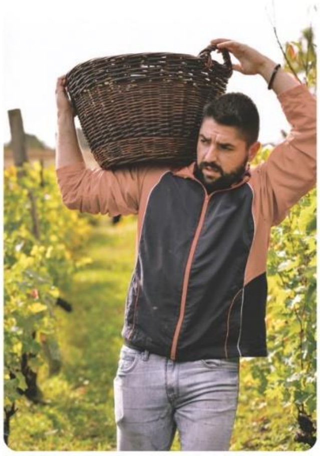

Swiss TPH, Kreuzstrasse 2, 4123 Allschwil, Schweiz. www.swisstph.ch

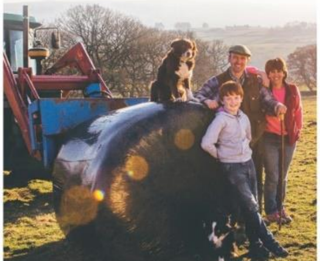

Eine landwirtschaftliche Studie für und mit Produzenten und deren Ehe- oder Lebenspartner/innen

Gesunde und qualitativ hochwertige Nahrungsmittel sind für alle Menschen unerlässlich.

Obwohl Landwirte und Landwirtinnen die Basis für unsere Ernährung schaffen, sind ihre Gesundheit und Lebensqualität in der Schweiz kaum untersucht. Auch das Wohlbefinden und die Gesundheit von Ehe- oder Lebenspartner/innen von Landwirten und Landwirtinnen ist kaum erforscht, unabhängig davon, ob die Partner/innen selbst in der Landwirtschaft tätig sind oder nicht.

Das **Schweizerische Tropen- und Public Health-Institut** untersucht daher unterschiedliche Aspekte der Gesundheit von der landwirtschaftlichen Bevölkerung in der Schweiz und hilft so, die wichtigsten Gesundheitsprobleme und risiken zu erkennen und zu reduzieren. Dazu arbeiten wir mit Personen aus landwirtschaftlichen Verbänden, Politik und Wirtschaft zusammen, um von Anfang an sicherzustellen, dass unsere Ergebnisse zu einer besseren Politikgestaltung beitragen können. Aussagekräftige Ergebnisse erhalten wir jedoch nur mit Ihrer Unterstützung.

Wir laden Sie daher ein, an unserer Gesundheitsstudie teilzunehmen.

Mit Ihrer Teilnahme tragen Sie aktiv zur Erforschung der Gesundheit und Lebensqualität von Personen und deren Partner/innen in der Schweizer Landwirtschaft bei.

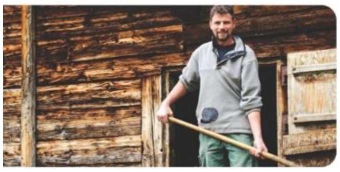

Gesundheitsfragebogen für Personen und deren Partner/innen in der Schweizer Landwirtschaft

Wenn Sie in der Schweizer Landwirtschaft tätig sind, laden wir Sie herzlich ein, bei der Gesundheitsumfrage mitzumachen.

Teilnahmebedingungen

- Sie und/oder Ihr/e Ehe- oder Lebenspartner/in sind derzeit in der Schweizer Landwirtschaft tätig.
- Sie haben Ihren festen Wohnsitz in der Schweiz.
- Sie sind 18 Jahre alt oder älter.

Was erwartet mich als Teilnehmer/in?

Teilnehmende werden einen Gesundheitsfragebogen ausfüllen. Der Fragebogen wird Ihnen per E-Mail oder Post zugeschickt. Das Ausfüllen dauert ungefähr 40 Minuten.

Warum soll ich mitmachen?

Unter allen Teilnehmenden, welche den Fragebogen ausfüllen, werden **100 Gutscheine à je 50 Schweizer Franken** für einen Einkauf bei LANDI (www.landi.ch) verlost. Als Teilnehmer/in leisten Sie zudem einen wichtigen Beitrag zur Erhaltung und Verbesserung der Gesundheit und Lebensqualität von Personen in der Schweizer Landwirtschaft.

Registrierung

Bei Interesse an einer Teilnahme können Sie sich auf unserer Webseite in wenigen Schritten registrieren:

www.swisstph.ch/farmcoswiss

Die Registrierung dient dazu, dass wir Ihnen Informationen zur Studie und ein Anmeldeformular per Post zusenden können.

Mit der Registrierung gehen Sie keine Verbindlichkeiten ein und nehmen noch nicht an der Umfrage Teil.

Einordnung der Studie

Der Gesundheitsfragebogen ist Teil des nationalen Forschungsprojektes TRAPEGO (www.trapego.ch), welches wünschenswerte, machbare und faire Lösungen im Bereich Landwirtschaft, Nachhaltigkeit und Pflanzenschutz zum Ziel hat. Innerhalb dieses Projekts werden mehrere Studien durchgeführt.

Dieser Gesundheitsfragebogen beschäftigt sich mit der **generellen Gesundheit der landwirtschaftlichen Bevölkerung in der Schweiz** und ist unabhängig vom Thema Pflanzenschutz entwickelt worden. Bei einer Teilnahme an diesem Fragebogen nehmen Sie nicht automatisch an anderen Studien von TRAPEGO Teil.

S2 Study flyer in French

Information et contact :

www.swisstph.ch/farmcoswiss_fr
farmcoswiss@swisstph.ch
061 284 89 29

FarmCoSwiss

Schweizerischer Nationalfonds

Le projet est soutenu par le Fonds national suisse (FNS) No. CRSII5_193762

FarmCoSwiss

Swiss TPH
Swiss Tropical and Public Health Institute
Schweizerisches Tropen- und Public Health-Institut
Institut Tropical et de Santé Publique Suisse

Associated Institute of the University of Basel

QU'EN EST-IL DE LA SANTÉ DES PERSONNES ET DE LEURS PARTENAIRES DANS L'AGRICULTURE SUISSE ?

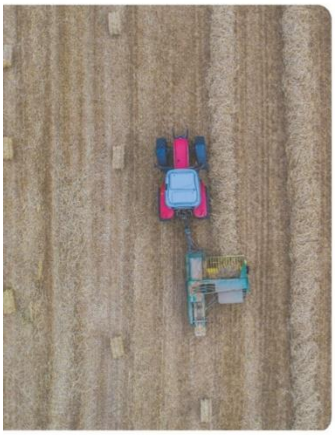

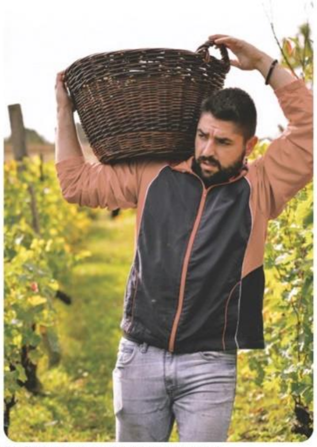

Swiss TPH, Kreuzstrasse 2, 4123 Allschwil, Suisse. www.swisstph.ch

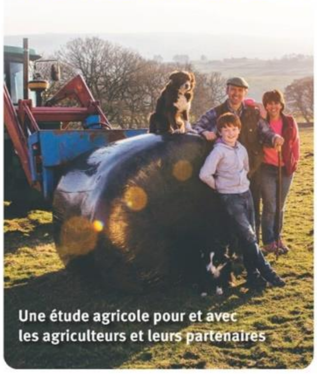

Une alimentation saine et de qualité est indispensable à tous les êtres humains.

Bien que les agriculteurs et les agricultrices créent la base de notre alimentation, leur santé et leur qualité de vie ne sont guère étudiées en Suisse. De même, le bien-être et la santé des partenaires d'agriculteurs n'ont guère été étudiés, que les partenaires soient eux-mêmes actifs dans l'agriculture ou non.

L'**Institut Tropical et de Santé Publique Suisse** étudie donc différents aspects de la santé de la population agricole en Suisse et aide ainsi à identifier et à réduire les principaux problèmes et risques sanitaires. Pour ce faire, nous coopérons avec des personnes issues d'associations agricoles, de la politique et de l'économie, afin de garantir dès le départ que nos résultats puissent contribuer à une meilleure élaboration des politiques. Toutefois, nous n'obtiendrons des résultats significatifs qu'avec votre soutien.

Nous vous invitons donc à participer à notre étude sur la santé.

En participant, vous contribuez activement à la recherche sur la santé et la qualité de vie des personnes et leurs partenaires dans l'agriculture suisse.

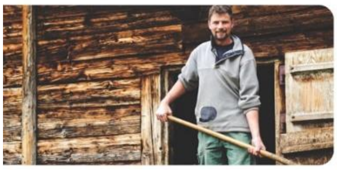

Questionnaire de santé pour les personnes et leurs partenaires dans l'agriculture suisse

Si vous et/ou votre partenaire travaillez dans l'agriculture suisse, nous vous invitons à participer à notre l'enquête sur la santé.

Conditions de participation

- Vous et/ou votre partenaire travaillez actuellement dans l'agriculture suisse.
- Vous résidez en Suisse de manière permanente ou pour une longue durée.
- Vous avez 18 ans ou plus.

Qu'est-ce qui m'attend en tant que participant(e) ?

Les participant(e)s rempliront un questionnaire de santé. Le questionnaire leur sera envoyé par e-mail ou par courrier. Le remplir prend environ 40 minutes.

Pourquoi devrais-je participer ?

100 bons de 50 francs suisses chacun pour un achat chez LANDI (www.landi.ch) seront tirés au sort parmi tous les participant(e)s qui auront rempli le questionnaire. En tant que participant(e), vous apportez en outre une contribution importante au maintien et à l'amélioration de la santé et de la qualité de vie des personnes dans l'agriculture suisse.

Inscription

Si vous êtes intéressé(e) à participer, vous pouvez vous inscrire en quelques étapes sur notre site web :

www.swisstph.ch/farmcoswiss_fr

L'enregistrement nous permet de vous envoyer des informations sur l'étude et un formulaire d'inscription par courrier.

En vous inscrivant, vous ne prenez aucun engagement et ne participez pas encore à l'enquête.

Classement de l'étude

Le questionnaire sur la santé fait partie du projet de recherche national TRAPEGO (www.trapego.ch), qui vise à trouver des solutions souhaitables, réalisables et équitables dans le domaine de l'agriculture, de la durabilité et de la protection des plantes. Plusieurs études sont menées dans le cadre de ce projet.

Ce questionnaire de santé porte sur la **santé générale de la population agricole en Suisse** et a été développé indépendamment du thème de la protection des plantes. En participant à ce questionnaire, vous ne participez pas automatiquement à d'autres études de TRAPEGO.

S3 Study flyer in Italian

Informazioni e contatti:

www.swisstph.ch/farmcoswiss_it
farmcoswiss@swisstph.ch
061 284 89 29

FarmCoSwiss

Schweizerischer Nationalfonds

Il progetto è finanziato dal Fondo Nazionale Svizzero per la Scienza (FNS) No. CRSII5_193762

FarmCoSwiss

Swiss TPH
Swiss Tropical and Public Health Institute
Schweizerisches Tropen- und Public Health-Institut
Institut Tropical et de Santé Publique Suisse
Associated Institute of the University of Basel

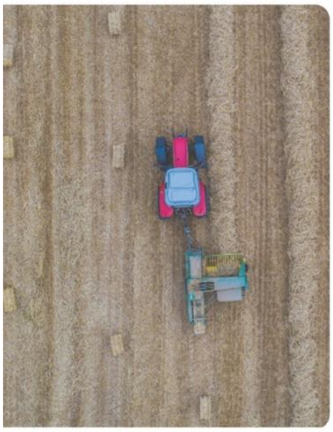

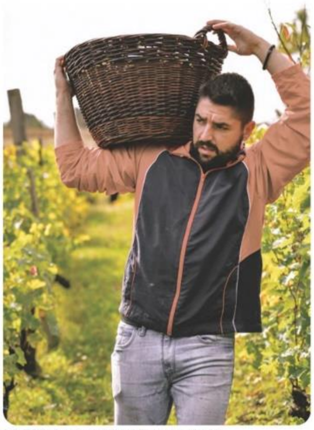

Swiss TPH, Kreuzstrasse 2, 4123 Allschwil, Svizzera. www.swisstph.ch

CHE DIRE DELLA **SALUTE** DELLE PERSONE E I LORO CONIUGI/PARTNER NELL' **AGRICOLTURA SVIZZERA?**

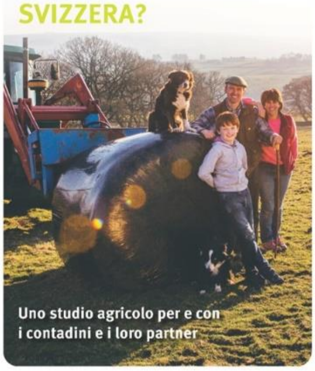

Il cibo sano è qualità essenziale per tutte le persone.

Anche se i contadini creano le basi della nostra alimentazione, la loro salute e la qualità delle loro vite sono poco studiate in Svizzera. Esistono anche poche ricerche sul benessere e la salute dei partner degli contadini, indipendentemente dal fatto che i partner stessi lavorino nell'agricoltura o meno.

L'**Istituto Svizzero per la salute pubblica e quella tropicale** studia quindi i diversi aspetti della salute delle persone nell'agricoltura svizzera, aiutando ad identificare e ridurre i più importanti problemi e rischi per la loro salute. Per fare ciò, lavoriamo con le associazioni agricole, la politica e l'economia, al fine di assicurare, fin dall'inizio, che le nostre scoperte possano contribuire a migliorare le loro vite. Tuttavia, siamo in grado di ottenere risultati significativi soltanto con il Suo prezioso contributo.

La invitiamo quindi a partecipare al nostro studio sulla salute dei contadini.

Facendo ciò, contribuirà attivamente alla ricerca sulla salute e la qualità delle vite delle persone e dei loro partner nell'agricoltura svizzera.

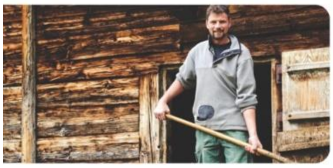

Questionario sulla salute per le persone e i loro partner nell'agricoltura svizzera

Se Lei e/o Suo/a partner lavora nell'agricoltura svizzera, La invitiamo cordialmente a partecipare al sondaggio sulla salute.

Condizioni di partecipazione

- Lei e/o il Suo/la Sua partner lavora attualmente nell'agricoltura svizzera.
- Vive in Svizzera in modo permanente o per un periodo prolungato.
- Ha 18 anni o più.

Cosa dovrò aspettarmi come partecipante?

I partecipanti saranno sottoposti a un questionario sulla salute. Il questionario le sarà inviato per e-mail o per posta. Ci vorranno circa 40 minuti per completarlo.

Perché dovrei partecipare?

Tutti i partecipanti che completeranno il questionario parteciperanno all'estrazione di **100 buoni di 50 franchi svizzeri,** ciascuno per un acquisto presso LANDI (www.landi.ch). Come partecipante, sta inoltre fornendo un importante contributo al mantenimento, al miglioramento della salute e della qualità di vita delle persone nell'agricoltura svizzera.

Registrazione

Se è interessato/a a partecipare, può registrarsi in pochi passi sul nostro sito web:

www.swisstph.ch/farmcoswiss_it

Lo scopo della registrazione è quello di permetterci di inviarle informazioni sullo studio e un modulo di registrazione per posta.

Registrandosi, non sta assumendo alcun obbligo e non sta ancora partecipando al sondaggio.

Classificazione dello studio

Il questionario sulla salute fa parte del progetto di ricerca nazionale TRAPEGO (www.trapego.ch), che mira a soluzioni desiderabili, fattibili ed eque nel campo dell'agricoltura, della sostenibilità e della protezione delle piante. Nell'ambito di questo progetto si stanno conducendo diversi studi.

Questo questionario sanitario riguarda la **salute generale della popolazione agricola in Svizzera** ed è stato sviluppato indipendentemente dal tema della protezione delle piante. Partecipando a questo questionario, non partecipa automaticamente ad altri studi TRAPEGO.

S4 Questionnaire in German

Swiss TPH

Swiss Tropical and Public Health Institute
Schweizerisches Tropen- und Public Health-Institut
Institut Tropical et de Santé Publique Suisse

Assoziiertes Institut der Universität Basel

FarmCoSwiss: Fragebogen zur Gesundheit von Personen und deren Partner/innen in der Schweizer Landwirtschaft

Sehr geehrter Teilnehmer, sehr geehrte Teilnehmerin

Das Schweizerische Tropen- und Public Health-Institut (Swiss TPH) untersucht mit diesem Fragebogen, wie es um die allgemeine **Gesundheit und das Wohlbefinden von in der Schweizer Landwirtschaft tätigen Personen und deren Ehe- oder Lebenspartner/in** (nachfolgend der Einfachheit halber "Partner/in" genannt) steht. Mit Ihrer Teilnahme leisten Sie einen wichtigen Beitrag zur Einschätzung und Verbesserung der Gesundheit von der landwirtschaftlichen Bevölkerung in der Schweiz. Bitte füllen Sie den Fragebogen ohne Unterbruch aus. Das Ausfüllen des Fragebogens dauert ungefähr 40 Minuten.

Wir nehmen die Einhaltung des Datenschutzes und die Wahrung der Vertraulichkeit sehr ernst. Alle erhobenen Angaben werden daher **streng vertraulich** behandelt, **verschlüsselt** ausgewertet und ausschliesslich für wissenschaftliche Zwecke verwendet.

Abhängig davon, ob Sie entweder

a) selbst in der Landwirtschaft tätig sind oder
b) ausserhalb der Landwirtschaft tätig sind, aber Ihr Partner/Ihre Partnerin in der Landwirtschaft arbeitet, werden Sie leicht andere Fragen im Fragebogen beantworten.

Fragen, die für gewisse Personen übersprungen werden können, sind deutlich mit einem Pfeil ➡ markiert.

Bei Fragen oder Unklarheiten können Sie das Studienteam unter 061 284 89 29 oder farmcoswiss@swisstph.ch erreichen.

Herzlichen Dank für Ihre Teilnahme!

Prof. Dr. Nicole Probst-Hensch

Papierfragebogen

Bitte füllen Sie den Fragebogen mit einem schwarzen oder blauen Kugelschreiber aus.
Senden Sie den fertig ausgefüllten Fragebogen im beigelegten vorfrankierten und -adressierten Briefumschlag bitte zurück an das Schweizerische Tropen- und Public Health-Institut.

Herzlichen Dank!

Einstiegsfragen

0	**Bitte geben Sie das Datum an, an welchem Sie den Fragebogen ausfüllen.**

☐☐ ☐☐ ☐☐☐☐

Tag Monat Jahr

0.1	**Sind Sie selbst auf einem landwirtschaftlichen Betrieb tätig?**<br>Unter "tätig sein" verstehen wir Personen, die selbstständig erwerbend, angestellt (Voll- oder Teilzeit) oder in Ausbildung sind, oder Saisonarbeit oder freiwillige Mithilfe auf einem Landwirtschaftsbetrieb leisten (z.B. als Familienanghörige oder Lebenspartner/in). Tätigkeiten auf dem Betrieb können sowohl landwirtschaftlicher als auch administrativer Art sein.<br><br>➡ Falls nein: Gehen Sie bitte direkt zu Frage 0.2.

☐ Ja ☐ Nein

0.1.1	**Falls Sie selbst auf einem landwirtschaftlichen Betrieb tätig sind: Nehmen andere Personen, die auf dem selben landwirtschaftlichen Betrieb wie Sie selbst tätig sind, ebenfalls an dieser Umfrage teil (ausser Ehe- oder Lebenspartner/in)? Falls ja: Wie viele Personen?**

☐ Ja ☐ Nein ☐ Weiss nicht

Falls ja: Anzahl Personen: ……………………

0.2	**Nimmt Ihr/e Partner/in ebenfalls an dieser Umfrage teil?**

☐ Ja ☐ Nein ☐ Weiss nicht ☐ Ich habe keine/n Partner/in.

0.2.2	**Falls Ihr/e Partner/in ebenfalls an der Umfrage teilnimmt: Bitte geben Sie den Vor- und Nachnamen sowie das Geburtsdatum Ihres Partners/Ihrer Partnerin an.** Diese Informationen benötigen wir, um Sie und Ihre/n Partner/in einander zuordnen zu können. ➡ Falls nein, weiss nicht oder kein/e Partner/in: Gehen Sie bitte direkt zum Kapitel "Kontaktangaben".

Vorname: ……………………………………………………………………………………………

Nachname: ……………………………………………………………………………………………

Geburtsdatum: ☐☐ ☐☐ ☐☐☐☐

Tag Monat Jahr

In diesem Fragebogen beziehen sich einige Fragen auf den landwirtschaftlichen Betrieb, auf dem Sie arbeiten. Bitte beantworten Sie diese Fragen für den Betrieb, auf dem Sie in den <u>letzten 12 Monaten die meiste Zeit</u> gearbeitet haben. Falls Sie selbst nicht in der Landwirtschaft tätig sind, beantworten Sie die Fragen bitte für den Betrieb, auf dem Ihr/e Partner/in in den letzten 12 Monaten die meiste Zeit gearbeitet hat. Alle Angaben werden streng vertraulich behandelt.

Kontaktangaben

1.1	**Bitte geben Sie Ihren Vor- und Nachnamen an.**

Vorname: ……………………………………………………………………………………

Nachname: ……………………………………………………………………………………

1.2	**Haben sich Ihre Wohn- und/oder Betriebsadresse, welche Sie bei der Registrierung angegeben haben, seither geändert?**

☐ Ja

Neue Wohnadresse: …………………………………………………………..

Ab wann ist diese neue Wohnadresse gültig? (TT/MM/JJJJ) …………………………………………………………..

Neue Betriebsadresse: …………………………………………………………..

Ab wann ist diese neue Betriebsadresse gültig? (TT/MM/JJJJ) …………………………………………………………..

☐ Nein

1.3	**Haben sich Ihre Telefonnummer und/oder E-Mail-Adresse, welche Sie bei der Registrierung angegeben haben, seither geändert?**

☐ Ja

Neue Telefonnummer: …………………………………………………………..

Neue E-Mail-Adresse: …………………………………………………………..

☐ Nein

Ausbildung

2.1	**Was ist Ihr höchster Bildungsabschluss?** Bitte nur eine Antwort ankreuzen.

☐ Obligatorische Primar- und Oberstufe

☐ Lehre/Berufsschule/Handelsschule

☐ Kantonsschule/Gymnasium

☐ Höhere Fach- oder Berufsschule

☐ Universität/Hochschule

☐ Anderes, bitte präzisieren: …………………………………………………………......

2.2	**Was ist Ihre landwirtschaftliche Grundausbildung?** Mehrere Antworten sind möglich.

- ☐ Keine landwirtschaftliche Grundausbildung
- ☐ Agrarpraktiker/in (EBA)
- ☐ Landwirt/in
- ☐ Geflügelfachmann/-frau
- ☐ Gemüsegärtner/in
- ☐ Obstfachmann/-frau
- ☐ Winzer/in
- ☐ Weintechnologe/in
- ☐ Andere, bitte präzisieren: ………………………………………………………….

2.3	**Was ist Ihre höhere landwirtschaftliche Berufsbildung?** Mehrere Antworten sind möglich.

- ☐ Keine höhere landwirtschaftliche Berufsbildung
- ☐ Bäuerin/bäuerlicher Haushaltleiter
- ☐ Landwirtschaftliche Berufsprüfung (Betriebsleiterschule)
- ☐ Höhere Fachprüfung/Meisterprüfung (Betriebsleiterschule 2)
- ☐ Höhere Fachschule: Agrokaufmann/-frau
- ☐ Höhere Fachschule: Agrotechniker/in
- ☐ Höhere Fachschule: Weinbautechniker/in
- ☐ Andere, bitte präzisieren: ………………………………………………………….

2.4	**Wie viele Jahre Ihres Lebens haben Sie insgesamt auf einem landwirtschaftlichen Betrieb gearbeitet?** Bitte nur eine Antwort ankreuzen.

Nie	1-5 Jahre	6-10 Jahre	11-20 Jahre	21-30 Jahre	Mehr als 30 Jahre
☐	☐	☐	☐	☐	☐

2.5	**Wie viele Jahre Ihres Lebens haben Sie insgesamt auf einem landwirtschaftlichen Betrieb gelebt?** Bitte nur eine Antwort ankreuzen.

Nie	1- 5 Jahre	6-10 Jahre	11-20 Jahre	21-30 Jahre	Mehr als 30 Jahre
☐	☐	☐	☐	☐	☐

2.6	**Sind Sie auf einem landwirtschaftlichen Betrieb aufgewachsen?**<br>Bitte nur eine Antwort ankreuzen.	
Ja	Nein	Anderes, bitte präzisieren:
☐	☐	☐ ………………………..……..…………………………

Erwerbstätigkeit

3.1		**In welcher beruflichen Stellung sind Sie auf dem landwirtschaftlichen Betrieb tätig?**<br>Mehrere Antworten sind möglich.<br>➡ Falls Sie nicht auf einem landwirtschaftlichen Betrieb tätig sind, gehen Sie bitte direkt zu Frage 3.1.1.
a	☐	(Mit-)Bewirtschafter/in (Betriebsleitung)
b	☐	Mitarbeit in Familienbetrieb (mit AHV-pflichtigem Einkommen)
c	☐	Mitarbeit in Familienbetrieb (ohne AHV-pflichtiges Einkommen)
d	☐	Angestellt mit Cheffunktion (unterstellte Mitarbeiter)
e	☐	Angestellt ohne Cheffunktion (keine unterstellten Mitarbeiter)
f	☐	Pensioniert mit Arbeitstätigkeit
g	☐	In Ausbildung
h	☐	Andere, bitte präzisieren: …………………………….………………………

3.1.1	**Falls Sie nicht auf einem landwirtschaftlichen Betrieb tätig sind: In welcher beruflichen Stellung sind Sie zurzeit tätig?** Mehrere Antworten sind möglich.<br>➡ Falls Sie auf einem landwirtschaftlichen Betrieb tätig sind, gehen Sie bitte direkt zu Frage 3.2.	
a	☐	Selbstständig erwerbend
b	☐	Angestellt mit Cheffunktion (unterstellte Mitarbeiter)
c	☐	Angestellt ohne Cheffunktion (keine unterstellten Mitarbeiter)
d	☐	Pensioniert
e	☐	In Ausbildung
f	☐	Hausfrau/Hausmann
g	☐	Invalid
h	☐	Auf Stellensuche
i	☐	Andere, bitte präzisieren: …………………………………………………………

3.2	**Arbeiten Sie haupt- oder nebenberuflich auf einem landwirtschaftlichen Betrieb?** Hauptberuflich ist eine Arbeit dann, wenn sie von der **wirtschaftlichen Bedeutung** und dem **zeitlichen Aufwand** her die übrigen Erwerbstätigkeiten deutlich übersteigt.<br>Bitte nur eine Antwort ankreuzen.<br>➡ Falls Sie nicht auf einem landwirtschaftlichen Betrieb tätig sind, gehen Sie bitte direkt zu Frage 3.2.1.
	☐ Hauptberuflich
	☐ Nebenberuflich
	☐ Anderes, bitte präzisieren: …………………………………………………………

3.2.1	**Falls Sie nicht auf einem landwirtschaftlichen Betrieb tätig sind: In welchem Pensum führen Sie Ihre Erwerbstätigkeit aus?** Bitte nur eine Antwort ankreuzen.<br>➡ Falls Sie auf einem landwirtschaftlichen Betrieb tätig sind, gehen Sie bitte direkt zu Frage 3.4
	☐ Vollzeit
	☐ Teilzeit
	☐ Anderes, bitte präzisieren: …………………………………………………………

3.3	**Falls Sie nicht auf einem landwirtschaftlichen Betrieb tätig sind: In welcher Wirtschaftsbranche sind Sie zurzeit tätig?** Mehrere Antworten sind möglich.<br>➡ Falls Sie auf einem landwirtschaftlichen Betrieb tätig sind, gehen Sie bitte direkt zum Frage 3.4.

- ☐ Verarbeitendes Gewerbe (z.B. Herstellung von Waren)/Energieversorgung
- ☐ Baugewerbe
- ☐ Handel und Reperaturgewerbe/Verkehr und Lagerei/Gastgewerbe
- ☐ Information und Kommunikation
- ☐ Kredit- und Versicherungsgewerbe
- ☐ Immobilien/Sonstige wirtschaftliche Dienstleistungen
- ☐ Freiberufliche Arbeit/Administrative, technische, wissenschaftliche Dienstleistungen
- ☐ Öffentliche Verwaltung/Erziehung und Unterricht/Gesundheits- und Sozialwesen
- ☐ Kunst/Unterhaltung/Sonstige Dienstleistungen
- ☐ Andere, bitte präzisieren: ……………………………………………………………

3.4	**Arbeiten Sie zusätzlich in einem Beruf ausserhalb der Landwirtschaft?**<br>Bitte nur eine Antwort ankeuzen.<br>➡ Falls Sie nicht auf einem landwirtschaftlichen Betrieb tätig sind, gehen Sie bitte direkt zu zum Kapitel "Allgemeine Gesundheit"

- ☐ Nein
- ☐ Ja, ganzjährig und weniger als 50% der Arbeitszeit
- ☐ Ja, ganzjährig und 50% oder mehr der Arbeitszeit
- ☐ Ja, nur in der **kälteren** Jahreszeit und **weniger als 50%** der Arbeitszeit
- ☐ Ja, nur in der **kälteren** Jahreszeit und **50% oder mehr** der Arbeitszeit
- ☐ Ja, nur in der **wärmeren** Jahreszeit und **weniger als 50%** der Arbeitszeit
- ☐ Ja, nur in der **wärmeren** Jahreszeit und **50% oder mehr** der Arbeitszeit
- ☐ Anderes, bitte präzisieren: ……………………………………………………………

Falls ja: Bitte nennen Sie die Berufsbezeichnung Ihres Berufs ausserhalb der Landwirtschaft.

Berufsbezeichnung: ………………………………………………………………………………

➡ **Falls Sie selbst nicht auf einem landwirtschaftlichen Betrieb tätig sind, überspringen Sie bitte alle Fragen im Kapitel "Hofmanagement und Haushaltsstruktur" und gehen Sie direkt zum Kapitel "Allgemeine Gesundheit".**

Hofmanagement und Haushaltsstruktur

4.1 Wer besitzt den landwirtschaftlichen Betrieb?
Mehrere Antworten sind möglich.

- ☐ Sie selbst
- ☐ Ihr/e (Ehe-)Partner/in
- ☐ Ihr Vater/Ihre Mutter
- ☐ Ihr Bruder/Ihre Schwester
- ☐ Anderes Familienmitglied
- ☐ Nicht-verwandte Person
- ☐ Anderes, bitte präzisieren: ....

4.2 Was ist die betriebswirtschaftliche Ausrichtung des Betriebs?
Mehrere Antworten sind möglich.

- ☐ Weideviehbetrieb - Milchwirtschaft
- ☐ Weideviehbetrieb - Fleischproduktion
- ☐ Veredelungsbetrieb - Schweinehaltung
- ☐ Veredelungsbetrieb - Geflügelhaltung
- ☐ Ackerbaubetrieb
- ☐ Spezialkulturbetrieb (z.B. Obst, Gemüse, Beeren oder Reben)
- ☐ Gartenbaubetrieb
- ☐ Forstwirtschaftsbetrieb
- ☐ Andere, bitte präzisieren: ....

4.3	**Welche Kulturen werden auf dem Betrieb angebaut bzw. bewirtschaftet und wie viele Hektaren pro Kultur ungefähr? Falls auf dem Betrieb kein Pflanzenbau betrieben wird, überspringen Sie bitte die Frage.** Mehrere Antworten sind möglich.

	**Hektaren**				
	Weniger als 5	5-10	11-20	21-50	Mehr als 50
☐ Getreide	☐	☐	☐	☐	☐
☐ Hülsenfrüchte	☐	☐	☐	☐	☐
☐ Ölsaaten	☐	☐	☐	☐	☐
☐ Zuckerrüben/ Futterrüben/Kartoffeln	☐	☐	☐	☐	☐
☐ Gemüse	☐	☐	☐	☐	☐
☐ Obst/Steinobst	☐	☐	☐	☐	☐
☐ Beeren	☐	☐	☐	☐	☐
☐ Reben	☐	☐	☐	☐	☐
☐ Wald	☐	☐	☐	☐	☐
☐ Andere, bitte präzisieren:	☐	☐	☐	☐	☐
……………………………..					

4.4	**Wie gross ist der Betrieb insgesamt ungefähr?** Bitte nur eine Antwort ankreuzen.

- ☐ Kleiner als 5 Hektaren
- ☐ 5-10 Hektaren
- ☐ 11-20 Hektaren
- ☐ 21-50 Hektaren
- ☐ Grösser als 50 Hektaren

4.5	**Welche Produktionsform wird auf dem Betrieb angewendet?** Mehrere Antworten sind möglich.

- ☐ Konventionell
- ☐ Integrierte Produktion (IP)
- ☐ Biologisch
- ☐ Aktuell Umstellung auf biologische Produktion
- ☐ Anderes, bitte präzisieren: ……………………………………………………………………

4.6	**Seit wie vielen Jahren wird diese Produktionsform ungefähr angewendet bzw. auf biologisch umgestellt?** Mehrere Antworten sind möglich.

- ☐ Konventionell: ______________________ (in ganzen Jahren)
- ☐ Integrierte Produktion: ______________________ (in ganzen Jahren)
- ☐ Biologisch: ______________________ (in ganzen Jahren)
- ☐ Umstellung auf biologische Produktion: ______________________ (in ganzen Jahren)
- ☐ Anderes: ……………………………… ______________________ (in ganzen Jahren)

4.7	**Was sind die drei wichtigsten Haupteinnahmequellen des Betriebs? Nennen Sie bitte 3 Produkte oder Dienstleistungen, welche die grössten Einnahmen für den Betrieb generieren.** Antworten können in beliebiger Reihenfolge genannt werden.

1 ……………………………………………………………………………………………

2 ……………………………………………………………………………………………

3 ……………………………………………………………………………………………

4.8	**Wie viele Personen leben permanent oder längerfristig in Ihrem Haushalt, Sie selbst eingeschlossen?**

Anzahl Erwachsene (Sie selbst eingeschlossen):	☐☐
Anzahl Personen unter 18 Jahren:	☐☐

4.9	**Wie viele Personen arbeiten auf dem Betrieb, Sie selbst eingeschlossen?** Dies beinhaltet Teil- und Vollzeitmitarbeitende, Saisonmitarbeitende, sowie z.B. Familienmitglieder, die auf dem Betrieb ohne Entgeld mitarbeiten.
**Kältere Jahreszeit** Anzahl Erwachsene (Sie selbst eingeschlossen):	☐☐☐
**Wärmere Jahreszeit** Anzahl Erwachsene (Sie selbst eingeschlossen):	☐☐☐

Allgemeine Gesundheit

5.1 Wie würden Sie Ihren Gesundheitszustand im Allgemeinen beschreiben?
Bitte nur eine Antwort ankreuzen.

☐ Ausgezeichnet ☐ Sehr gut ☐ Gut ☐ Weniger gut ☐ Schlecht

5.2 Die folgenden Fragen beschreiben Tätigkeiten, die Sie vielleicht an einem normalen Tag ausüben. Geben Sie bitte für jede dieser Tätigkeiten an, <u>ob Ihr Gesundheitszustand Sie derzeit bei der Ausübung dieser Tätigkeiten einschränkt.</u>
Bitte für jede Tätigkeit (a-b) nur eine Antwort ankreuzen. Falls die Tätigkeitsbeispiele für Sie nicht relevant sind, beantworten Sie die Frage für eine Tätigkeit mit einer ähnlichen körperlichen Intensität.

		Ja, stark eingeschränkt	Ja, etwas eingeschränkt	Nein, überhaupt nicht eingeschränkt
a	Mittelschwere Tätigkeiten, z.B. einen Tisch verschieben, staubsaugen oder kegeln	☐	☐	☐
b	Mehrere Treppenabsätze steigen	☐	☐	☐

5.3 Wie oft hatten Sie in den vergangenen 4 Wochen aufgrund Ihrer <u>körperlichen</u> Gesundheit folgende Schwierigkeiten bei der Arbeit oder anderen alltäglichen Tätigkeiten im Beruf bzw. zu Hause?
Bitte für jede Aussage (a-b) nur eine Antwort ankreuzen.

		Immer	Meistens	Manchmal	Selten	Nie
a	Ich habe weniger geschafft, als ich wollte.	☐	☐	☐	☐	☐
b	Ich konnte nur bestimmte Dinge tun.	☐	☐	☐	☐	☐

5.4 Wie oft hatten Sie in den vergangenen 4 Wochen aufgrund <u>seelischer Probleme</u> folgende Schwierigkeiten bei der Arbeit oder anderen alltäglichen Tätigkeiten im

	**Beruf bzw. zu Hause (z. B. weil Sie sich niedergeschlagen oder ängstlich fühlten)?** Bitte für jede Aussage (a-b) nur eine Antwort ankreuzen.

		Immer	Meistens	Manchmal	Selten	Nie
a	Ich habe weniger geschafft, als ich wollte.	☐	☐	☐	☐	☐
b	Ich konnte nicht so sorgfältig wie üblich arbeiten.	☐	☐	☐	☐	☐

5.5	**Inwieweit haben Schmerzen Sie in den vergangenen 4 Wochen bei der Ausübung Ihrer Alltagstätigkeiten zu Hause und im Beruf behindert?** Bitte nur eine Antwort ankreuzen.

☐ Überhaupt nicht ☐ Etwas ☐ Mässig ☐ Ziemlich ☐ Sehr

5.6	**In diesen Fragen geht es darum, wie Sie sich fühlen und wie es Ihnen in den vergangenen 4 Wochen gegangen ist. Bitte wählen Sie für jede Frage die Antwort aus, die Ihrem Befinden am ehesten entspricht.** Bitte für jede Aussage (a-c) nur eine Antwort ankreuzen.

**Wie oft waren Sie in den vergangenen 4 Wochen…**		Immer	Meistens	Manchmal	Selten	Nie
a	ruhig und gelassen?	☐	☐	☐	☐	☐
b	voller Energie?	☐	☐	☐	☐	☐
c	entmutigt und traurig?	☐	☐	☐	☐	☐

5.7	**Wie häufig haben Ihre körperliche Gesundheit oder seelischen Probleme in den vergangenen 4 Wochen Ihre Kontakte zu anderen Menschen (Besuche bei Freunden, Verwandten usw.) beeinträchtigt?** Bitte nur eine Antwort ankreuzen.

☐ Immer ☐ Meistens ☐ Manchmal ☐ Selten ☐ Nie

Bewegung, Ernährung, Alkohol und Tabak

6.1	**Wie viel Stunden pro Tag verbringen Sie ungefähr mit Sitzen?**		
a	An einem Arbeitstag in der kälteren Jahreszeit	☐☐	Stunden pro Tag
b	An einem Ruhetag in der kälteren Jahreszeit	☐☐	Stunden pro Tag
c	An einem Arbeitstag in der wärmeren Jahreszeit	☐☐	Stunden pro Tag
c	An einem Ruhetag in der wärmeren Jahreszeit	☐☐	Stunden pro Tag

6.2 **An wie vielen Tagen pro Woche sind Sie in Ihrem landwirtschaftlichen Beruf insgesamt 30 Minuten oder länger körperlich so aktiv, dass Sie ausser Atem geraten oder schwitzen?** Bitte nur eine Antwort ankreuzen.

➡ Falls Sie nicht auf einem landwirtschaftlichen Betrieb tätig sind, gehen Sie bitte direkt zu Frage 6.2.1.

	Tage pro Woche							
	0	1	2	3	4	5	6	7
a Kältere Jahreszeit	☐	☐	☐	☐	☐	☐	☐	☐
b Wärmere Jahreszeit	☐	☐	☐	☐	☐	☐	☐	☐

6.2.1	**Falls Sie nicht auf einem landwirtschaftlichen Betrieb tätig sind: An wie vielen Tagen pro Woche sind Sie <u>in Ihrem Beruf</u> insgesamt 30 Minuten oder länger körperlich so aktiv, dass Sie ausser Atem geraten oder schwitzen?** Bitte nur eine Antwort ankreuzen.<br>➡ Falls Sie auf einem landwirtschaftlichen Betrieb tätig sind, gehen Sie bitte direkt zu Frage 6.3.

	**Tage pro Woche**							
a Kältere Jahreszeit	0 ☐	1 ☐	2 ☐	3 ☐	4 ☐	5 ☐	6 ☐	7 ☐
	**Tage pro Woche**							
b Wärmere Jahreszeit	0 ☐	1 ☐	2 ☐	3 ☐	4 ☐	5 ☐	6 ☐	7 ☐

☐ Ich bin nicht erwerbstätig.

6.3	**An wie vielen Tagen pro Woche sind Sie <u>in der Freizeit</u> insgesamt 30 Minuten oder länger körperlich so aktiv, dass Sie ausser Atem geraten oder schwitzen?**<br>Bitte nur eine Antwort ankreuzen.

	**Tage pro Woche**							
a Kältere Jahreszeit	0 ☐	1 ☐	2 ☐	3 ☐	4 ☐	5 ☐	6 ☐	7 ☐
	**Tage pro Woche**							
b Wärmere Jahreszeit	0 ☐	1 ☐	2 ☐	3 ☐	4 ☐	5 ☐	6 ☐	7 ☐

6.4	**An wie vielen Tagen pro Woche essen Sie im Allgemeinen…?**<br>Bitte für jedes Produkt (a-d) nur eine Antwort ankreuzen.

		**Tage pro Woche**								
	Nie	Seltener als 1 Tag pro Woche	1	2	3	4	5	6	7	Weiss nicht
a Rotes Fleisch oder Wurst	☐	☐	☐	☐	☐	☐	☐	☐	☐	☐
b Gekochtes Gemüse	☐	☐	☐	☐	☐	☐	☐	☐	☐	☐
c Rohes Gemüse oder Salat	☐	☐	☐	☐	☐	☐	☐	☐	☐	☐
d Früchte	☐	☐	☐	☐	☐	☐	☐	☐	☐	☐

6.5	**Wie viele Portionen … nehmen Sie durchschnittlich pro Tag zu sich, wenn Sie Gemüse oder Früchte essen?** Eine Portion entpsricht etwa einer Hand voll/einer grossen Tomate. Bitte für jedes Produkt (a-c) nur eine Antwort ankreuzen.

		Weniger als 1 Portion	1 Portion	2-4 Portionen	5 Portionen oder mehr	Weiss nicht
a	Rohes Gemüse oder Salat	☐	☐	☐	☐	☐
b	Gekochtes Gemüse	☐	☐	☐	☐	☐
c	Früchte	☐	☐	☐	☐	☐

6.6	**Wie oft nehmen Sie folgende alkoholische Getränke zu sich?** Bitte für jedes Getränk (a-b) nur eine Antwort ankreuzen.

		Nie	1-4-mal im Monat oder weniger	Mehrmals pro Woche	Täglich
a	Bier/Wein/Sekt/Vergorener Most	☐	☐	☐	☐
b	Schnaps/Likör	☐	☐	☐	☐

6.7	**Konsumieren Sie eines der folgenden Tabakprodukte?** Bitte für jede Aussage (a-c) nur eine Antwort ankreuzen.

		Nie geraucht	Früher, jetzt nicht mehr	Ja, an manchen Tagen	Ja, täglich
a	Zigaretten	☐	☐	☐	☐
b	E-Zigaretten	☐	☐	☐	☐
c	Andere Tabakprodukte	☐	☐	☐	☐

6.8	**Wie schwer sind Sie ungefähr? Für Frauen, wenn Sie zur Zeit schwanger sind: Wie schwer waren Sie am Anfang der Schwangerschaft ungefähr?** Bitte geben Sie an, ob das Gewicht von Ihnen geschätzt ist oder ob Sie sich gewogen haben.

☐☐☐ kg ☐ geschätzt ☐ gewogen

6.9	**Wie gross sind Sie ungefähr?**
	☐☐☐ cm

➡ **Falls Sie selbst nicht auf einem landwirtschaftlichen Betrieb tätig sind, überspringen Sie bitte alle Fragen im Kapitel "Ihre Einschätzung von Risiken im Berufsalltag" und gehen Sie direkt zum Kapitel "Lebensqualität, Schlaf und Stress".**

Ihre Einschätzung von Risiken im Berufsalltag

In der landwirtschaftlichen Gesundheitsforschung wird grundsätzlich zwischen 5 Risikobereichen unterschieden:

- Physisch (z.B. längeres Sitzen, manuelle Arbeiten)
- Chemisch (z.B. Düngemittel, Pflanzenschutzmittel, Gase und andere Gefahrenstoffe)
- Biologisch (z.B. übertragbare Krankheiten, Pollen, Staub)
- Psychosozial (z.B. soziale Isolation, Konflikte/Streit, Stress/Druck)
- Umwelt-bezogen (z.B. Stürme, Hitze/Kälte, Erdrutsche)

Gerne möchten wir von Ihnen wissen:
a) **wie oft** Sie durchschnittlich **in den letzten 12 Monaten** einzelnen Risiken aus den fünf Bereichen in Ihrem **landwirtschaftlichen Berufsalltag** ausgesetzt waren und
b) als **wie gesundheitsschädlich** Sie die einzelnen Risiken einschätzen, **unabhängig davon, wie oft Sie persönlich ausgesetzt waren.**

Pro Bereich sind jeweils vier Tätigkeiten (Risiken) aufgelistet. In der letzten freien Zeile können Sie selbst noch ein Risiko, welches Ihrer Meinung nach noch unbedingt erwähnt werden sollte, aufschreiben und die dazugehörigen Felder ankreuzen.

"Nie" bedeutet, dass auf dem Betrieb solche Tätigkeiten ausgeführt werden, aber nicht von Ihnen, oder dass andere Personen solchen Risiken ausgesetzt sind, aber nicht Sie.

"Nicht relevant" bedeutet, dass auf dem landwirtschaftlichen Betrieb niemand solche Tätigkeiten ausführt/solchen Risiken ausgesetzt ist.

7.1	**Physische Risiken:**<br>**Wie oft übten Sie durchschnittlich in den letzten 12 Monaten folgende Tätigkeiten (a-d) in Ihrem landwirtschaftlichen Alltag aus?**<br>**Unabhängig davon, wie oft Sie diese ausübten: Für wie gesundheitsschädlich halten Sie die einzelnen Tätigkeiten?**

		Häufigkeit						Gesundheitsschädlichkeit			
		Nie	Selten	Gelegentlich	Oft	Immer	Nicht relevant	Nicht gesundheits-schädlich	Etwas	Ziemlich	Sehr gesundheits-schädlich
a	Schwere körperliche Arbeiten (z.B. Tragen von Lasten, manuelles Mähen)	☐	☐	☐	☐	☐	☐	☐	☐	☐	☐
b	Bedienung von Fahrzeugen/Maschinen	☐	☐	☐	☐	☐	☐	☐	☐	☐	☐
c	Arbeiten in der Höhe oder auf schwierigem Gelände (z.B. auf einer Leiter/einem Dach oder an Hängen)	☐	☐	☐	☐	☐	☐	☐	☐	☐	☐
d	Längeres Sitzen (4 oder mehr Stunden am Stück)	☐	☐	☐	☐	☐	☐	☐	☐	☐	☐
e	…………………………....	☐	☐	☐	☐	☐	☐	☐	☐	☐	☐

7.2	**Chemische Risiken:**<br>**Wie oft übten Sie durchschnittlich in den letzten 12 Monaten folgende Tätigkeiten (a-d) in Ihrem landwirtschaftlichen Alltag aus?**<br>**Unabhängig davon, wie oft Sie diese ausübten: Für wie gesundheitsschädlich halten Sie die einzelnen Tätigkeiten?**

		Häufigkeit						Gesundheitsschädlichkeit			
		Nie	Selten	Gelegentlich	Oft	Immer	Nicht relevant	Nicht gesundheits-schädlich	Etwas	Ziemlich	Sehr gesundheits-schädlich
a	Ausbringen von Düngemitteln	☐	☐	☐	☐	☐	☐	☐	☐	☐	☐
b	Mischen und/oder Ausbringen von Pflanzenschutzmitteln (z.B. Herbizide, Fungizide oder Insektizide)	☐	☐	☐	☐	☐	☐	☐	☐	☐	☐
c	Tätigkeiten, bei denen Sie mit Gasen oder Rauch in Kontakt kommen (z.B. von Gülle)	☐	☐	☐	☐	☐	☐	☐	☐	☐	☐
d	Anwendung von ätzenden Reinigungsmitteln (z.B. Landmaschinen-reiniger)	☐	☐	☐	☐	☐	☐	☐	☐	☐	☐
e	....	☐	☐	☐	☐	☐	☐	☐	☐	☐	☐

7.3	**Biologische Risiken:**<br>**Wie oft übten Sie durchschnittlich in den letzten 12 Monaten folgende Tätigkeiten (a-d) in Ihrem landwirtschaftlichen Alltag aus?**<br>**Unabhängig davon, wie oft Sie diese ausübten: Für wie gesundheitsschädlich halten Sie die einzelnen Tätigkeiten?**

	**Arbeiten, bei denen Sie…**	Häufigkeit						Gesundheitsschädlichkeit			
		Nie	Selten	Gelegentlich	Oft	Immer	Nicht relevant	Nicht gesundheits-schädlich	Etwas	Ziemlich	Sehr gesundheits-schädlich
a	mit Pollen/Sporen in Kontakt kommen (z.B. Gräserpollen oder Pilzsporen)	☐	☐	☐	☐	☐	☐	☐	☐	☐	☐
b	mit Staub in Kontakt kommen (z.B. Holzstaub oder Heustaub)	☐	☐	☐	☐	☐	☐	☐	☐	☐	☐
c	mit Tieren oder Tierexkreten in Kontakt kommen (z.B. Kontakt ohne Zaun/Anbindung der Tiere oder durch Mist/Blut)	☐	☐	☐	☐	☐	☐	☐	☐	☐	☐
d	möglicherweise von Insekten gestochen oder gebissen werden (z.B. Bienen oder Zecken)	☐	☐	☐	☐	☐	☐	☐	☐	☐	☐
e	…………………………	☐	☐	☐	☐	☐	☐	☐	☐	☐	☐

7.4	**Psychosoziale Risiken:**<br>**Wie oft waren Sie durchschnittlich in den letzten 12 Monaten folgenden psychosozialen Risiken (a-d) in Ihrem landwirtschaftlichen Alltag ausgesetzt?**<br>**Unabhängig davon, wie oft Sie persönlich ausgesetzt waren: Für wie gesundheitsschädlich halten Sie die einzelnen Risiken?**

		Häufigkeit						Gesundheitsschädlichkeit			
		Nie	Selten	Gelegentlich	Oft	Immer	Nicht relevant	Nicht gesundheits-schädlich	Etwas	Ziemlich	Sehr gesundheits-schädlich
a	Konflikte (z.B. mit Mitarbeitenden, in der Familie oder Nachbarschaft)	☐	☐	☐	☐	☐	☐	☐	☐	☐	☐
b	Schlafprobleme	☐	☐	☐	☐	☐	☐	☐	☐	☐	☐
c	Stress/Druck	☐	☐	☐	☐	☐	☐	☐	☐	☐	☐
d	Einsamkeit	☐	☐	☐	☐	☐	☐	☐	☐	☐	☐
e	....	☐	☐	☐	☐	☐	☐	☐	☐	☐	☐

7.5	**Umwelt-bezogene Risiken:**<br>**Wie oft waren Sie durchschnittlich in den letzten 12 Monaten folgenden Umwelt-bezogenen Risiken (a-d) in Ihrem landwirtschaftlichen Alltag ausgesetzt?**<br>**Unabhängig davon, wie oft Sie persönlich ausgesetzt waren: Für wie gesundheitsschädlich halten Sie die einzelnen Risiken?**

		Häufigkeit						Gesundheitsschädlichkeit			
		Nie	Selten	Gelegentlich	Oft	Immer	Nicht relevant	Nicht gesundheits-schädlich	Etwas	Ziemlich	Sehr gesundheits-schädlich
a	Arbeiten draussen an der prallen Sonne	☐	☐	☐	☐	☐	☐	☐	☐	☐	☐
b	Stürme (z.B. mit Hagel, Blitz oder viel Niederschlag)	☐	☐	☐	☐	☐	☐	☐	☐	☐	☐
c	Erdrutsche/Lawinen	☐	☐	☐	☐	☐	☐	☐	☐	☐	☐
d	Lärm	☐	☐	☐	☐	☐	☐	☐	☐	☐	☐
e	....	☐	☐	☐	☐	☐	☐	☐	☐	☐	☐

Lebensqualität, Schlaf und Stress

8.1	**Sie finden unten 12 Fragen und Aussagen (a-l). Bitte beantworten Sie jede Frage auf der Skala von 0 bis 10.** Bitte für jede Frage/Aussage (a-l) nur eine Antwort ankreuzen.											
		0 = Überhaupt nicht zufrieden										*10 = Vollständig zufrieden*
a	Ganz allgemein gefragt – wie zufrieden sind Sie mit Ihrem Leben?	0 ☐	1 ☐	2 ☐	3 ☐	4 ☐	5 ☐	6 ☐	7 ☐	8 ☐	9 ☐	10 ☐
		0 = Überhaupt nicht glücklich										*10 = Vollständig glücklich*
b	Ganz allgemein gefragt – wie glücklich oder unglücklich fühlen Sie sich?	0 ☐	1 ☐	2 ☐	3 ☐	4 ☐	5 ☐	6 ☐	7 ☐	8 ☐	9 ☐	10 ☐
		0 = Schlecht										*10 = Exzellent*
c	Ganz allgemein gefragt – wie beurteilen Sie Ihre körperliche Gesundheit?	0 ☐	1 ☐	2 ☐	3 ☐	4 ☐	5 ☐	6 ☐	7 ☐	8 ☐	9 ☐	10 ☐
		0 = Schlecht										*10 = Exzellent*
d	Ganz allgemein gefragt – wie beurteilen Sie Ihre psychische Gesundheit?	0 ☐	1 ☐	2 ☐	3 ☐	4 ☐	5 ☐	6 ☐	7 ☐	8 ☐	9 ☐	10 ☐
		0 = Überhaupt nicht lohnend										*10 = Absolut lohnend*
e	Ganz allgemein gefragt – in welchem Ausmass haben Sie das Gefühl, dass das was Sie im Leben tun, sinnvoll und lohnend ist?	0 ☐	1 ☐	2 ☐	3 ☐	4 ☐	5 ☐	6 ☐	7 ☐	8 ☐	9 ☐	10 ☐
		0 = Überhaupt nicht einverstanden										*10 = Absolut einverstanden*
f	Wie sehr sind Sie mit der folgenden Aussage einverstanden: «Ich weiss, was der Sinn meines Lebens ist»?	0 ☐	1 ☐	2 ☐	3 ☐	4 ☐	5 ☐	6 ☐	7 ☐	8 ☐	9 ☐	10 ☐

		0 = Trifft nicht auf mich zu										*10 = Trifft absolut auf mich zu*
g	Inwieweit trifft die folgende Aussage auf Sie zu: «Ich handle immer so, dass auf jeden Fall das Gute gefördert wird, selbst in schwierigen und herausfordernden Situationen»?	0 ☐	1 ☐	2 ☐	3 ☐	4 ☐	5 ☐	6 ☐	7 ☐	8 ☐	9 ☐	10 ☐
		0 = Trifft nicht auf mich zu										*10 = Trifft absolut auf mich zu*
h	Inwieweit trifft die folgende Aussage auf Sie zu: «Ich kann heute auf einen Teil Glück verzichten, wenn durch diesen Verzicht mein Glück zu einem späteren Zeitpunkt umso grösser ist»?	0 ☐	1 ☐	2 ☐	3 ☐	4 ☐	5 ☐	6 ☐	7 ☐	8 ☐	9 ☐	10 ☐
		0 = Überhaupt nicht einverstanden										*10 = Absolut einverstanden*
i	Inwieweit sind Sie mit der folgenden Aussage einverstanden: «Ich bin zufrieden mit den Freundschaften und Beziehungen, die ich habe»?	0 ☐	1 ☐	2 ☐	3 ☐	4 ☐	5 ☐	6 ☐	7 ☐	8 ☐	9 ☐	10 ☐
		0 = Überhaupt nicht einverstanden										*10 = Absolut einverstanden*
j	Inwieweit sind Sie mit der folgenden Aussage einverstanden: «Meine Beziehungen sind so zufriedenstellend wie ich mir das wünsche»?	0 ☐	1 ☐	2 ☐	3 ☐	4 ☐	5 ☐	6 ☐	7 ☐	8 ☐	9 ☐	10 ☐
		0 = Ich mache mir immer Sorgen										*10 = Ich mache mir nie Sorgen*
k	Wie häufig machen Sie sich Sorgen, dass Sie sich die normalen monatlichen Lebenskosten nicht leisten können?	0 ☐	1 ☐	2 ☐	3 ☐	4 ☐	5 ☐	6 ☐	7 ☐	8 ☐	9 ☐	10 ☐
		0 = Ich mache mir immer Sorgen										*10 = Ich mache mir nie Sorgen*
l	Wie oft machen Sie sich Sorgen in Bezug auf Sicherheit, Ernährung oder Wohnen?	0 ☐	1 ☐	2 ☐	3 ☐	4 ☐	5 ☐	6 ☐	7 ☐	8 ☐	9 ☐	10 ☐

8.2	**Wie viele Stunden pro Nacht schliefen Sie durchschnittlich in den letzten 12 Monaten? Das muss nicht mit der Anzahl der Stunden, die Sie im Bett gelegen haben, übereinstimmen.** Bitte in ganzen Stunden angeben.

a Kältere Jahreszeit	☐☐	pro Nacht
b Wärmere Jahreszeit	☐☐	pro Nacht

8.3	**Wie würden Sie insgesamt die Qualität Ihres Schlafes in den letzten 12 Monaten beurteilen?** Bitte nur eine Antwort ankreuzen.

	Sehr gut	Gut	Mittelmässig	Schlecht	Sehr schlecht
a Kältere Jahreszeit	☐	☐	☐	☐	☐
b Wärmere Jahreszeit	☐	☐	☐	☐	☐

8.4	**Wie häufig ist es Ihnen in den letzten 12 Monaten passiert, dass Sie…** Bitte für jede Frage (a-d) nur eine Antwort ankreuzen.

	Nie	Selten	Manchmal	Häufig
a Schlecht einschlafen?	☐	☐	☐	☐
b Einen unruhigen Schlaf haben?	☐	☐	☐	☐
c Nachts mehrmals erwachen?	☐	☐	☐	☐
d Morgens zu früh erwachen?	☐	☐	☐	☐

8.5	**Auf einer Skala von 1 bis 6, wie würden Sie Ihre Fähigkeit mit Stress umzugehen beurteilen? (1 = Ich kann Stress von mir abschütteln, 6 = Stress nagt an mir)** Bitte nur eine Antwort ankreuzen.

Kann Stress abschütteln 1	2	3	4	5	Stress nagt an mir 6
☐	☐	☐	☐	☐	☐

8.6	**In den letzten 12 Monaten, wie hoch würden Sie das Ausmass an Stress in Ihrem Leben beurteilen (zu Hause und bei der Arbeit)?** **(1 = kein Stress, 6 = extremer Stress)** Bitte nur eine Antwort ankreuzen.					
	Kein Stress 1	2	3	4	5	Extremer Stress 6
a Kältere Jahreszeit	☐	☐	☐	☐	☐	☐
b Wärmere Jahreszeit	☐	☐	☐	☐	☐	☐

8.7	**Wie haben sich folgende Aspekte Ihrer Gesundheit gesamthaft während der Coronavirus-Pandemie (seit März 2020) verändert?** Bitte für jede Aussage (a-e) nur eine Antwort ankreuzen.				
	Viel schlechter geworden	Eher schlechter geworden	Überhaupt nicht verändert	Eher besser geworden	Viel besser geworden
a Körperliche Gesundheit	☐	☐	☐	☐	☐
b Seelische Gesundheit	☐	☐	☐	☐	☐
c Lebensqualität	☐	☐	☐	☐	☐
d Schlafqualität	☐	☐	☐	☐	☐
e Gesunder Lebensstil (Ernährung, körperliche Betätigung, Alkohol-/Tabakkonsum)	☐	☐	☐	☐	☐

Arbeitszufriedenheit

9.1 In den letzten 12 Monaten, wie zufrieden waren Sie insgesamt mit Ihrer Arbeit auf dem landwirtschaftlichen Betrieb?
Bitte nur eine Antwort ankreuzen.
➡ Falls Sie nicht auf einem landwirtschaftlichen Betrieb tätig sind, gehen Sie bitte direkt zu Frage 9.1.1.

- ☐ Sehr zufrieden
- ☐ Ziemlich zufrieden
- ☐ Teils-teils
- ☐ Ziemlich unzufrieden
- ☐ Sehr unzufrieden

9.1.1	**Falls Sie nicht auf einem landwirtschaftlichen Betrieb tätig sind: In den letzten 12 Monaten, wie zufrieden waren Sie insgesamt mit Ihrer Arbeit?** Bitte nur eine Antwort ankreuzen.

- ☐ Sehr zufrieden
- ☐ Ziemlich zufrieden
- ☐ Teils-teils
- ☐ Ziemlich unzufrieden
- ☐ Sehr unzufrieden

9.2	**Unabhängig davon, ob es sich dabei um bezahlte oder unbezahlte Arbeit (z.B. Haushalt) handelte: Wie oft arbeiteten Sie in den letzten 12 Monaten…**

**Kältere Jahreszeit**	Nie	Selten	Manchmal	Häufig
a Mehr als 5 Tage pro Woche?	☐	☐	☐	☐
b Mehr als 10 Stunden pro Tag?	☐	☐	☐	☐
**Wärmere Jahreszeit**				
c Mehr als 5 Tage pro Woche?	☐	☐	☐	☐
d Mehr als 10 Stunden pro Tag?	☐	☐	☐	☐

9.3	**Welche der folgenden Dinge waren für Sie die <u>3 wichtigsten Gründe</u>, Ihren landwirtschaftlichen Beruf zu wählen?** Nennen Sie bitte 3 Dinge.<br>➡ Falls Sie nicht auf einem landwirtschaftlichen Betrieb tätig sind, gehen Sie bitte direkt zum Kapitel "Erkrankungen und Unfälle".

- ☐ Finanzielle Sicherheit
- ☐ Freiheit, die Arbeit selbstständig zu gestalten
- ☐ Sinnhaftigkeit des Berufs
- ☐ Soziale Beziehungen zu Mitarbeitenden, Bekannten, Familie und/oder Nachbarn
- ☐ Flexible Arbeitszeiten
- ☐ Gesellschaftliche Anerkennung
- ☐ Körperliche Arbeit/Arbeit draussen in der Natur
- ☐ Arbeitsplatzsicherheit
- ☐ Anderes, bitte präzisieren: ……………………………………………………………………
- ☐ Weiss nicht

9.4	**Angenommen, Sie entscheiden sich, Ihren landwirtschaftlichen Beruf aufzugeben: Welche der folgenden Dinge wären für Sie die <u>3 wahrscheinlichsten Gründe</u>, Ihren landwirtschaftlichen Beruf aufzugeben?** Nennen Sie bitte 3 Dinge.

- ☐ Finanzieller Druck
- ☐ Zu wenig Freizeit/Ferien
- ☐ Fehlende gesellschaftliche Anerkennung
- ☐ Einsamkeit
- ☐ Streitigkeiten im Betrieb, der Familie oder Nachbarschaft
- ☐ Politischer oder gesellschaftlicher Druck
- ☐ Gesundheitliche Probleme
- ☐ Unregelmässige/lange Arbeitszeiten
- ☐ Probleme, eine Nachfolge für den Betrieb zu finden
- ☐ Anderes, bitte präzisieren: ……………………………………………………………………
- ☐ Weiss nicht

Erkrankungen und Unfälle

Alle Angaben werden streng vertraulich behandelt.

10.1 **Hat Ihnen ein Arzt je gesagt, dass Sie eine der folgenden Krankheiten haben?**
Bitte für jede Krankheit (a-l) nur eine Antwort ankreuzen. Falls Sie eine Arztdiagnose für eine Krankheit haben, beantworten Sie bitte zusätzlich die Frage in den grau eingefärbten Feldern.

			In welchem Jahr wurde das ungefähr zum ersten Mal diagnostiziert?	Nehmen Sie regelmässig vom Arzt verschriebene Medikamente für diese Krankheit/Beschwerden?
a	Rückenschmerzen über 3 Monate oder länger und zwar fast täglich	☐ Ja →<br>☐ Nein<br>☐ Weiss nicht<br>☐ Keine Angabe	☐☐☐☐	☐ Ja ☐ Nein
b	Arthrose bzw. Gelenkverschleiss	☐ Ja →<br>☐ Nein<br>☐ Weiss nicht<br>☐ Keine Angabe	☐☐☐☐	☐ Ja ☐ Nein
c	Asthma	☐ Ja →<br>☐ Nein<br>☐ Weiss nicht<br>☐ Keine Angabe	☐☐☐☐	☐ Ja ☐ Nein

d	Chronische Bronchitis oder chronisch obstruktive Lungenerkrankung (COPD)	☐ Ja → ☐ Nein ☐ Weiss nicht ☐ Keine Angabe	☐☐☐☐	☐ Ja ☐ Nein
e	Allergien (Heuschnupfen, Nahrungsmittelallergie, Ekzem, Insektengiftallergie etc.)	☐ Ja → ☐ Nein ☐ Weiss nicht ☐ Keine Angabe	☐☐☐☐	☐ Ja ☐ Nein
f	Herzkreislauferkrankungen (Herzinfarkt, Herzschwäche, Herzrhythmusstörungen etc.)	☐ Ja → ☐ Nein ☐ Weiss nicht ☐ Keine Angabe	☐☐☐☐	☐ Ja ☐ Nein
g	Zuckerkrankheit bzw. Diabetes mellitus (inkl. Diabetesdiagnose während der Schwangerschaft)	☐ Ja → ☐ Nein ☐ Weiss nicht ☐ Keine Angabe	☐☐☐☐	☐ Ja ☐ Nein
h	Krebs (Wenn ja: Welche Krebsart? ………………………………….....................)	☐ Ja → ☐ Nein ☐ Weiss nicht ☐ Keine Angabe	☐☐☐☐	☐ Ja ☐ Nein

i	Schlaganfall	☐ Ja →<br>☐ Nein<br>☐ Weiss nicht<br>☐ Keine Angabe	☐☐☐☐	☐ Ja ☐ Nein
j	Parkinson-Syndrom, auch Schüttellähmung genannt	☐ Ja →<br>☐ Nein<br>☐ Weiss nicht<br>☐ Keine Angabe	☐☐☐☐	☐ Ja ☐ Nein
k	Depression oder Angststörung	☐ Ja →<br>☐ Nein<br>☐ Weiss nicht<br>☐ Keine Angabe	☐☐☐☐	☐ Ja ☐ Nein
l	Unfall/Verletzung/Sturz mit Arztbesuch (Wenn ja: Geschah dies bei der Arbeit oder in der Freizeit?<br>☐ Arbeit ☐ Freizeit)	☐ Ja →<br>☐ Nein<br>☐ Weiss nicht<br>☐ Keine Angabe	☐☐☐☐	☐ Ja ☐ Nein

10.2	**Wie häufig waren Sie in den letzten 12 Monaten an folgenden Orten?** Eine Überweisung von einem Spital in ein anderes zählt als ein Mal. Falls Sie in den letzten 12 Monaten an keinem dieser Orte waren, schreiben Sie bitte die Zahl 0 hin.
a	☐ Beim Arzt/Hausarzt für eine ambulante Behandlung<br>☐☐ Mal ☐ Weiss nicht
b	☐ Im Spital/in einer Spezialklinik **(ohne Übernachtung)**<br>☐☐ Mal ☐ Weiss nicht
c	☐ Im Spital/in einer Spezialklinik **(mit Übernachtung)**<br>☐☐ Mal ☐ Weiss nicht
d	☐ In einer medizinischen Notfallaufnahme<br>☐☐ Mal ☐ Weiss nicht
e	☐ Anderes, bitte präzisieren: ....<br>☐☐ Mal

Abschliessende Frage

11.1	**Dürfen wir Sie für eine zukünftige Gesundheitsbefragung oder –studie (z.B. einen Folge-Gesundheitsfragebogen) erneut kontaktieren?**
☐ Ja	☐ Nein

Ihre Meinung ist gefragt

12.1	**Falls Sie uns noch etwas mitteilen wollen, finden Sie hier Platz für Anregungen, Wünsche, Kommentare oder Kritik. Ihre Meinung ist uns wichtig.**

Herzlichen Dank für Ihre Antworten und Rückmeldungen!
Wir schätzen Ihre Teilnahme sehr.
Ihr Swiss TPH Team

S5 Questionnaire in French

Swiss TPH
Swiss Tropical and Public Health Institute
Schweizerisches Tropen- und Public Health-Institut
Institut Tropical et de Santé Publique Suisse

Assoziiertes Institut der Universität Basel

FarmCoSwiss: Questionnaire sur la santé des personnes dans l'agriculture suisse et de leurs partenaires

Cher participant, chère participante

L'Institut Tropical et de Santé Publique Suisse (Swiss TPH) étudie avec ce questionnaire l'état de **santé** général **et le bien-être des personnes travaillant dans l'agriculture suisse et de leur conjoint(e) ou partenaire** (ci-après dénommé(e) "partenaire" pour simplifier). Par votre participation, vous apportez une contribution importante à l'évaluation et à l'amélioration de la santé de la population agricole en Suisse. Veuillez remplir le questionnaire sans interruption. Cela vous prendra environ 40 minutes.

Nous prenons très au sérieux le respect de la protection des données et de la confidentialité. Toutes les données recueillies sont donc traitées de manière **strictement confidentielle**, analysées **sous forme cryptée** et utilisées exclusivement à des fins scientifiques.

Selon que

a) vous travaillez vous-même dans l'agriculture ou
b) vous travaillez en dehors de l'agriculture, mais que votre partenaire travaille dans l'agriculture,

vous répondrez à d'autres questions du questionnaire.

Les questions qui peuvent être sautées pour certaines personnes sont clairement indiquées par une flèche. ➡

Si vous avez des questions ou des doutes, vous pouvez contacter l'équipe de l'étude au 061 284 89 29 ou en écrivant à l'adresse e-mail suivante: farmcoswiss@swisstph.ch.

Merci beaucoup pour votre participation!

Prof. Nicole Probst-Hensch

Questionnaire papier

Veuillez remplir le questionnaire avec un stylo à bille noir ou bleu.
Veuillez renvoyer le questionnaire dûment rempli dans l'enveloppe pré-affranchie et pré-adressée ci-jointe à l'Institut Tropical et de Santé Publique Suisse.

Merci beaucoup!

Questions initiales

0	**Veuillez indiquer la date à laquelle vous remplissez le questionnaire.**

☐☐ ☐☐ ☐☐☐☐

Jour Mois Année

0.1	**Travaillez-vous sur une exploitation agricole?**<br>Par "travailler", nous entendons les personnes qui exercent une activité indépendante, qui sont employées (à temps plein ou à temps partiel) ou en formation, ou qui effectuent un travail saisonnier ou une aide bénévole sur une exploitation agricole (p. ex. en tant que membre de la famille ou partenaire). Les activités sur l'exploitation agicole peuvent être de nature agricole ou administrative.<br><br>➡ Si non: Veuillez passer directement à la question 0.2.

☐ Oui ☐ Non

0.1.1	**Si vous travaillez vous-même sur une exploitation agricole: Est-ce que d'autres personnes qui travaillent sur la même exploitation agricole que vous participent également à cette enquête (à l'exception de votre partenaire)?**<br>**Si oui: Combien de personnes?**

☐ Oui ☐ Non ☐ Ne sais pas

Si oui: Nombre de personnes: ………………….

0.2	**Votre partenaire participe-t-il/elle aussi à cette enquête?**

☐ Oui ☐ Non ☐ Ne sais pas ☐ Je n'ai pas de partenaire.

0.2.1	**Si votre partenaire participe également à l'enquête: Veuillez indiquer le prénom, le nom de famille et la date de naissance de votre partenaire.**<br>Nous avons besoin de ces informations pour pouvoir vous mettre en relation avec votre partenaire.<br><br>➡ Si non, ne sais pas ou pas de partenaire: Veuillez passer directement au chapitre "Informations de contact".

Prénom: ……………………………………………………………………………………………

Nom de famille: ……………………………………………………………………………………………

Date de naissance: ☐☐ ☐☐ ☐☐☐☐
Jour Mois Année

Dans ce questionnaire, certaines questions se rapportent à l'exploitation agricole sur laquelle vous travaillez. Veuillez répondre à ces questions pour l'exploitation sur laquelle vous avez travaillé <u>la plupart du temps</u> au cours des <u>12 derniers mois</u>. Si vous ne travaillez pas vous-même dans l'agriculture, veuillez répondre à ces questions pour l'exploitation dans laquelle votre partenaire a travaillé le plus longtemps au cours des 12 derniers mois.Toutes les données seront traitées de manière strictement confidentielle.

Informations de contact

1.1	**Veuillez indiquer votre prénom et votre nom de famille.**

Prénom: ……………………………………………………………………………………

Nom de famille: ……………………………………………………………………………………

1.2	**L'adresse de votre résidence et/ou de l'exploitation que vous avez indiquée lors de votre inscription à l'étude ont-elles changé depuis?**

☐ Oui

Nouvelle adresse de résidence: ....

A partir de quand cette nouvelle adresse de résidence est-elle valable? (JJ/MM/AAAA) ....

Nouvelle adresse d'exploitation: ....

À partir de quand cette nouvelle adresse d'exploitation est-elle valable? (JJ/MM/AAAA) ....

☐ Non

1.3	**Le numéro de téléphone ou l'adresse e-mail que vous avez indiqués lors de votre inscription ont-ils changé depuis?**

☐ Oui

Nouveau numéro de téléphone: ....

Nouvelle adresse e-mail: ....

☐ Non

Informations professionnelles

2.1	**Quel est votre niveau d'études le plus élevé?** Veuillez ne cocher qu'une seule réponse.

☐ Enseignement primaire et secondaire obligatoire

☐ Apprentissage/École professionnelle/École de commerce

☐ École cantonale/gymnase

☐ École technique ou professionnelle supérieure

☐ Université/Haute école

☐ Autre, veuillez préciser: ....

2.2	**Quelle est votre formation agricole de base?** Plusieurs réponses sont possibles.

- ☐ Pas de formation agricole de base
- ☐ Agropracticien/ne (AFP)
- ☐ Agriculteur/trice
- ☐ Spécialiste de la volaille
- ☐ Maraîcher/chère
- ☐ Expert(e) en fruits
- ☐ Viticulteur/trice
- ☐ Technicien//ne vitivinicole (ES)
- ☐ Autres, veuillez préciser: ....

2.3	**Quelle est votre formation professionnelle agricole supérieure ?** Plusieurs réponses sont possibles.

- ☐ Pas de formation professionnelle agricole supérieure
- ☐ Paysanne/responsable de ménage agricole
- ☐ Examen professionnel agricole (école de chef/fe d'exploitation)
- ☐ Examen professionnel supérieur/examen de maîtrise (école de chef/fe d'exploitation 2)
- ☐ École supérieure: agrocommerçant.e
- ☐ École professionnelle supérieure: agrotechnicien/ne
- ☐ École professionnelle supérieure: technicien/ne viticole
- ☐ Autres, veuillez préciser: ....

2.4	**Combien d'années au total de votre vie avez-vous <u>travaillé</u> sur une exploitation agricole?** Veuillez ne cocher qu'une seule réponse.

Jamais	1-5 ans	6-10 ans	11-20 ans	21-30 ans	Plus de 30 ans
☐	☐	☐	☐	☐	☐

2.5	**Combien d'années au total de votre vie avez-vous <u>vécu</u> sur une exploitation agricole?** Veuillez ne cocher qu'une seule réponse.

Jamais	1-5 ans	6-10 ans	11-20 ans	21-30 ans	Plus de 30 ans
☐	☐	☐	☐	☐	☐

2.6	**Avez-vous grandi sur une exploitation agricole?** Veuillez ne cocher qu'une seule réponse.		
Oui	Non	Autre, veuillez préciser:	
☐	☐	☐	....

Activité professionnelle

3.1	**Quelle position professionnelle occupez-vous sur l'exploitation agricole?** Plusieurs réponses sont possibles.<br>➡ Si vous ne travaillez pas sur une exploitation agricole, veuillez passer directement à la question 3.1.1.	
a	☐	(Co)gérant.e (direction de l'entreprise)
b	☐	Collaboration dans une entreprise familiale (avec revenu soumis à l'AVS)
c	☐	Collaboration dans une entreprise familiale (sans revenu soumis à l'AVS)
d	☐	Employé.e avec fonction de chef/fe (collaborateurs/trices subordonné.e.s)
e	☐	Employé.e sans fonction de chef/fe (pas de collaborateurs/trices subordonné.e.s)
f	☐	Retraité.e avec activité professionnelle
g	☐	En formation
h	☐	Autre, veuillez préciser: ....

3.1.1	**Si vous ne travaillez pas sur une exploitation agricole: Quelle est votre situation professionnelle actuelle? Plusieurs réponses sont possibles.**<br>➡ Si vous travaillez sur une exploitation agricole, veuillez passer directement à la question 3.2.	
a	☐	Indépendant.e
b	☐	Employé.e avec fonction de chef/fe (collaborateurs/trices subordonné.e.s)
c	☐	Employé.e sans fonction de chef/fe (pas de collaborateurs/trices subordonné.e.s)
d	☐	Retraité.e
e	☐	En formation
f	☐	Femme/homme au foyer
g	☐	Invalid.e
h	☐	À la recherche d'un emploi
i	☐	Autre, veuillez préciser: …………………………………………………………………..

3.2	**Travaillez-vous dans l'agriculture à titre principal ou secondaire?**<br>Un travail est considéré comme une activité principale lorsqu'il dépasse nettement les autres activités lucratives en termes d'**importance économique** et de **temps consacré**. Veuillez ne cocher qu'une seule réponse.<br>➡ Si vous ne travaillez pas sur une exploitation agricole, veuillez passer directement à la question 3.2.1.
	☐ Profession principale
	☐ Activité secondaire
	☐ Autre, veuillez préciser: ....

3.2.1	**Si vous ne travaillez pas sur une exploitation agricole: Quel est votre charge de travail?** Veuillez ne cocher qu'une seule réponse.<br>➡ Si vous travaillez sur une exploitation agricole, veuillez passer directement à la question 3.4.
	☐ Temps plein
	☐ Temps partiel
	☐ Autre, veuillez préciser: ………………………………………………………………

3.3	**Si vous ne travaillez pas dans une exploitation agricole: Dans quel secteur économique travaillez-vous actuellement?** Plusieurs réponses sont possibles.<br>➡ Si vous travaillez sur une exploitation agricole, veuillez passer directement à la question 3.4.

- a ☐ Industrie manufacturière (p. ex. production de biens)/Approvisionnement en énergie
- b ☐ Construction
- c ☐ Commerce et réparation/Transports etentreposage/Restauration
- d ☐ Information et communication
- e ☐ Activités financières et d'assurance
- f ☐ Immobilier/Autres services économiques
- g ☐ Travail indépendant/Services administratifs,techniques ou scientifiques
- h ☐ Administration publique/Éducation/Santé etaffaires sociales
- i ☐ Art/Divertissement/Autres services
- j ☐ Autre, veuillez préciser: ....

3.4	**Travaillez-vous également dans une profession autre que l'agriculture?**<br>Veuillez ne cocher qu'une seule réponse.<br>➡ Si vous ne travaillez pas sur une exploitation agricole, veuillez passer directement au chapitre "Santé générale" .

- ☐ Non
- ☐ Oui, toute l'année et moins de 50% du temps de travail
- ☐ Oui, toute l'année et 50% ou plus du temps de travail
- ☐ Oui, uniquement pendant la saison **froide** et **moins de 50%** du temps de travail
- ☐ Oui, uniquement pendant la saison **froide** et **50% ou plus** du temps de travail
- ☐ Oui, uniquement pendant la saison **chaude** et **moins de 50%** du temps de travail
- ☐ Oui, uniquement pendant la saison **chaude** et **50% ou plus** du temps de travail
- ☐ Autre, veuillez préciser: ....

Si oui: Veuillez indiquer le titre de votre profession en dehors de l'agriculture.

Titre de la profession: ....

➡ **Si vous ne travaillez pas vous-même sur une exploitation agricole, veuillez sauter toutes les questions du chapitre "Gestion de la ferme et structure budgétaire" et passer directement au chapitre "Santé générale".**

Gestion de la ferme et structure du ménage

4.1 Qui est propriétaire de l'exploitation agricole?
Plusieurs réponses sont possibles.

☐ Vous-même	☐ Votre partenaire	☐ Votre père/mère
☐ Votre frère/sœur	☐ Autre membre de la famille	☐ Personne non apparentée
☐ Autre, veuillez préciser: …………………………………………………………………………		

4.2 Quelle est l'orientation économique de l'exploitation agricole?
Plusieurs réponses sont possibles.

- ☐ Elevage de bétail sur pâturage - élevage laitier
- ☐ Elevage de bétail sur pâturage - production de viande
- ☐ Exploitation axée sur la transformation - élevage de porcs
- ☐ Exploitation axée sur la transformation - élevage de volailles
- ☐ Exploitation agricole
- ☐ Exploitation de cultures spéciales (p. ex. fruits, légumes, baies ou vignes)
- ☐ Entreprise horticole
- ☐ Exploitation forestière
- ☐ Autre, veuillez préciser: ………………………………………………………………

4.3	**Quelles cultures sont présentes et/ou exploitées sur le domaine et combien d'hectares environ par culture y a-t-il? Si aucune culture n'est pratiquée sur l'exploitation, veuillez sauter cette question.** Plusieurs réponses sont possibles.

		**Hectares**				
		Moins de 5	5-10	11-20	21-50	Plus de 50
☐	Céréales	☐	☐	☐	☐	☐
☐	Légumineuses	☐	☐	☐	☐	☐
☐	Oléagineux	☐	☐	☐	☐	☐
☐	Betterave à sucre/ Betteraves fourragères/pommes de terre	☐	☐	☐	☐	☐
☐	Légumes	☐	☐	☐	☐	☐
☐	Fruits/fruits à noyau	☐	☐	☐	☐	☐
☐	Baies	☐	☐	☐	☐	☐
☐	Vignes	☐	☐	☐	☐	☐
☐	Forêt	☐	☐	☐	☐	☐
☐	Autres, veuillez préciser:	☐	☐	☐	☐	☐

....

4.4	**Quelle est la taille approximative de l'exploitation?** Veuillez ne cocher qu'une seule réponse.

- ☐ Moins de 5 hectares
- ☐ 5-10 hectares
- ☐ 11-20 hectares
- ☐ 21-50 hectares
- ☐ Plus de 50 hectares

4.5	**Quelle forme de production est pratiquée sur l'exploitation?** Plusieurs réponses sont possibles.

- ☐ Conventionnelle
- ☐ Production intégrée (PI)
- ☐ Biologique
- ☐ Actuellement en reconversion vers la production biologique
- ☐ Autre, veuillez précisez: ....

4.6	**Depuis combien d'années environ ce mode de production est-il pratiqué, resp. depuis combien de temps êtes-vous en reconversion vers la production biologique?** Plusieurs réponses sont possibles.

☐ Conventionnelle: ____________________ (en années entières)

☐ Production intégrée: ____________________ (en années entières)

☐ Biologique: ____________________ (en années entières)

☐ Conversion vers la production biologique: ____________________ (en années entières)

☐ Autre: ____________________ (en années entières)

4.7	**Quelles sont les trois principales sources de revenus de l'exploitation? Veuillez citer 3 produits ou services qui génèrent les recettes les plus importantes pour l'exploitation.** Les réponses peuvent être données dans n'importe quel ordre.

1 ....

2 ....

3 ....

4.8	**Combien de personnes vivent en permanence ou pour une longue durée dans votre ménage, y compris vous-même ?**

Nombre d'adultes (y compris vous-même):	☐☐
Nombre de personnes de moins de 18 ans:	☐☐

4.9	**Combien de personnes travaillent sur l'exploitation, y compris vous-même ?**<br>Cela comprend les collaborateurs/trices à temps partiel et à temps plein, les collaborateurs/trices saisonniers/ières, ainsi que, p.ex., les membres de la famille qui travaillent sur l'exploitation sans être rémunéré.e.s.

**Saison froide**<br>Nombre d'adultes (y compris vous-même) :	☐☐☐
**Saison chaude**<br>Nombre d'adultes (y compris vous-même) :	☐☐☐

Santé générale

5.1	**Dans l'ensemble, pensez-vous que votre santé est:**<br>Veuillez ne cocher qu'une seule réponse.

☐ Excellente ☐ Très bonne ☐ Bonne ☐ Médiocre ☐ Mauvaise

5.2	**Voici une liste d'activités que vous pouvez avoir à faire dans votre vie de tous les jours. Pour chacune d'entre elles indiquez si vous êtes limité.e en raison de votre état de santé actuel.**<br>Veuillez ne cocher qu'une seule réponse par activité (a-b). Si les exemples d'activités ne vous concernent pas, répondez à la question pour une activité d'une intensité physique similaire.

		Oui, beaucoup limité.e	Oui, un peu limité.e	Non, pas du tout limité.e
a	Efforts physiques modérés, tels que déplacer une table, passer l'aspirateur ou jouer aux boules	☐	☐	☐
b	Monter plusieurs étages par l'escalier	☐	☐	☐

5.3	**Au cours des 4 dernières semaines, à quelle fréquence avez-vous rencontré les difficultés suivantes au travail ou dans d'autres activités quotidiennes au travail ou à la maison en raison de votre état de santé physique?**<br>Veuillez ne cocher qu'une seule réponse pour chaque affirmation (a-b).

		En permanence	Très souvent	Quelquefois	Rarement	Jamais
a	J'ai accompli moins de choses que j'aurais souhaité.	☐	☐	☐	☐	☐
b	J'ai dû arrêter de faire certaines choses.	☐	☐	☐	☐	☐

5.4	**Au cours des 4 dernières semaines, à quelle fréquence avez-vous rencontré les difficultés suivantes au travail ou dans d'autres activités quotidiennes au travail ou à la maison en raison de votre <u>état de santé émotionnel</u> (comme vous sentir triste, nerveux.se ou déprimé.e)?**<br>Veuillez ne cocher qu'une seule réponse par affirmation (a-b).

		En permanence	Très souvent	Quelquefois	Rarement	Jamais
a	J'ai accompli moins de choses que j'aurais souhaité.	☐	☐	☐	☐	☐
b	J'ai fait ce que j'avais à faire avec moins de soin et d'attention que d'habitude.	☐	☐	☐	☐	☐

5.5	**Au cours des 4 dernières semaines, dans quelle mesure vos douleurs physiques vous ont-elles limité.e dans votre travail ou vos activités domestiques?**<br>Veuillez ne cocher qu'une seule réponse.

☐ Pas du tout ☐ Un petit peu ☐ Moyennement ☐ Beaucoup ☐ Enormément

5.6	**Les questions qui suivent portent sur comment vous vous êtes senti.e au cours de ces 4 dernières semaines. Pour chaque question, veuillez indiquer la réponse qui vous semble la plus appropriée.**<br>Veuillez ne cocher qu'une seule réponse par affirmation (a-c).

	**Au cours de ces 4 dernières semaines, y a-t-il eu des moments où vous vous êtes senti.e**	En permanence	Très souvent	Quelquefois	Rarement	Jamais
a	calme et détendu.e?	☐	☐	☐	☐	☐
b	débordant.e d'énergie?	☐	☐	☐	☐	☐
c	triste et déprimé.e?	☐	☐	☐	☐	☐

5.7	**Au cours de ces 4 dernières semaines, y a-t-il eu des moments où votre état de santé, physique ou émotionnel, vous a gêné.e dans votre vie sociale et vos relations avec les autres, votre famille, vos amis, vos connaissances?**<br>Veuillez ne cocher qu'une seule réponse.

☐ En permanence ☐ Très souvent ☐ Quelquefois ☐ Rarement ☐ Jamais

Activité physique, alimentation, alcool et tabac

6.1	Combien d'heures par jour environ passez-vous assis.e?		
a	Lors d'une journée de travail durant la saison froide	☐☐	Heures par jour
b	Lors d'un jour de repos durant la saison froide	☐☐	Heures par jour
c	Lors d'une journée de travail durant la saison chaude	☐☐	Heures par jour
d	Lors d'un jour de repos durant la saison chaude	☐☐	Heures par jour

6.2 Combien de jours par semaine, dans le cadre de votre profession agricole, êtes-vous physiquement actif/ve pendant 30 minutes ou plus au total, au point d'être essoufflé.e ou de transpirer? Veuillez ne cocher qu'une seule réponse.

➡ Si vous ne travaillez pas sur une exploitation agricole, veuillez passer directement à la question 6.2.1.

	Jours par semaine							
a Saison froide	0 ☐	1 ☐	2 ☐	3 ☐	4 ☐	5 ☐	6 ☐	7 ☐
b Saison chaude	0 ☐	1 ☐	2 ☐	3 ☐	4 ☐	5 ☐	6 ☐	7 ☐

6.2.1 **Si vous ne travaillez pas sur une exploitation agricole: Combien de jours par semaine, <u>dans le cadre de votre profession</u>, êtes-vous physiquement actif/ve pendant 30 minutes ou plus au total, au point d'être essoufflé.e ou de transpirer?** Veuillez ne cocher qu'une seule réponse.

➡ Si vous travaillez sur une exploitation agricole, veuillez passer directement à la question 6.3.

	Jours par semaine							
	0	1	2	3	4	5	6	7
a Saison froide	☐	☐	☐	☐	☐	☐	☐	☐
b Saison chaude	☐	☐	☐	☐	☐	☐	☐	☐

☐ Je n'exercise pas d'activité professionnelle.

6.3 **Combien de jours par semaine, pendant <u>vos loisirs,</u> pratiquez-vous une activité physique d'une durée totale de 30 minutes ou plus, au point d'être essoufflé.e ou de transpirer?** Veuillez ne cocher qu'une seule réponse.

	Jours par semaine							
	0	1	2	3	4	5	6	7
a Saison froide	☐	☐	☐	☐	☐	☐	☐	☐
b Saison chaude	☐	☐	☐	☐	☐	☐	☐	☐

6.4 **En général, combien de jours par semaine mangez-vous... ?**
Veuillez ne cocher qu'une seule réponse pour chaque produit (a-d).

		Jours par semaine									
		Jamais	Moins d'une jour par semaine	1	2	3	4	5	6	7	Ne sais pas
a	Viande rouge ou saucisse	☐	☐	☐	☐	☐	☐	☐	☐	☐	☐
b	Légumes cuits	☐	☐	☐	☐	☐	☐	☐	☐	☐	☐
c	Légumes crus ou salade	☐	☐	☐	☐	☐	☐	☐	☐	☐	☐
d	Fruits	☐	☐	☐	☐	☐	☐	☐	☐	☐	☐

6.5	**Combien de portions ... consommez-vous en moyenne par jour?** Une portion correspond à environ une poignée/une grosse tomate. Veuillez ne cocher qu'une seule réponse pour chaque produit (a-c).					
		Moins d'une portion	1 portion	2-4 portions	5 portions ou plus	Ne sais pas
a	Légumes crus ou salade	☐	☐	☐	☐	☐
b	Légumes cuits	☐	☐	☐	☐	☐
c	Fruits	☐	☐	☐	☐	☐

6.6	**À quelle fréquence consommez-vous les boissons alcoolisées suivantes ?** Veuillez ne cocher qu'une seule réponse pour chaque boisson (a-b).				
		Jam ais	1 à 4 fois par mois ou moins	Plusieurs fois par semaine	Tous les jours
a	Bière/vin/vin mousseux/moût fermenté	☐	☐	☐	☐
b	Eau de vie/liqueur	☐	☐	☐	☐

6.7	**Consommez-vous l'un des produits du tabac suivants ?** Veuillez ne cocher qu'une seule réponse pour chaque produit (a-c).				
		Jamais fumé	Avant, plus maintenant	Oui, certains jours	Oui, tous les jours
a	Cigarettes	☐	☐	☐	☐
b	Cigarette électronique	☐	☐	☐	☐
c	Autres produits du tabac	☐	☐	☐	☐

6.8	**Quel est votre poids approximatif ? En cas de grossesse : quel était votre poids approximatif au début de la grossesse ?** Veuillez indiquer si le poids est une estimation de votre part ou si vous vous êtes pesée.
	☐☐☐ kg ☐ estimé ☐ pesé

6.9	**Quelle est votre taille approximative ?**
	☐☐☐ cm

➡**Si vous ne travaillez pas vous-même sur une exploitation agricole, veuillez sauter toutes les questions du chapitre "Votre évaluation des risques dans votre travail quotidien" et passer directement au chapitre "Qualité de vie, sommeil et stress".**

Votre évaluation des risques dans votre travail quotidien

Dans la recherche sur la santé dans le secteur agricole, on distingue en principe 5 domaines de risques:

- Physiques (p. ex. position assise prolongée, travail manuel)
- Chimiques (p. ex. engrais, produits phytosanitaires, gaz et autres substances dangereuses)
- Biologiques (p. ex. maladies transmissibles, pollen, poussière)
- Psychosociaux (p. ex. isolement social, conflits/litiges, stress/pression)
- Liés à l'environnement (p. ex. tempêtes, chaleur/froid, glissements de terrain)

Nous souhaiterions savoir
a) **la fréquence** moyenne **à laquelle** vous avez été exposé.e, **au cours des 12 derniers mois, à des** risques individuels relevant de ces cinq domaines dans votre **activité professionnelle agricole quotidienne** et
b) **à quel point** vous estimez que chaque risque **est dangereux pour la santé**, **indépendamment de la fréquence à laquelle vous avez été personnellement exposé.e.**

Quatre activités (risques) sont listées par domaine. Sur la dernière ligne libre, vous pouvez encore écrire vous-même un risque qui, selon vous, devrait encore absolument être mentionné et cocher les cases correspondantes.

"Jamais" signifie que de telles activités sont effectuées sur l'exploitation, mais pas par vous, ou que d'autres personnes sont exposées à de tels risques, mais pas vous.

"Non pertinent" signifie que personne n'effectue de telles activités/n'est exposé à de tels risques dans l'exploitation agricole.

7.1	**Risques physiques:**<br>**Au cours des 12 derniers mois, à quelle fréquence en moyenne avez-vous exercé les activités suivantes (a-d) dans votre travail agricole quotidien?**<br>**Indépendamment de la fréquence à laquelle vous les avez exercées, quel est le degré de nocivité de chacune d'entre elles à vos yeux?**

		Fréquence						Nocivité pour la santé			
		Jamais	Rarement	Occasionnelle-ment	Souvent	Toujours	Non pertinent	Pas nocif pour la santé	Légèrement	Assez	Très nocif pour la santé
a	Travaux physiques lourds (p. ex. porter des charges, tondre manuellement)	☐	☐	☐	☐	☐	☐	☐	☐	☐	☐
b	Conduite de grands véhicules/machines	☐	☐	☐	☐	☐	☐	☐	☐	☐	☐
c	Travailler en hauteur ou sur des terrains difficiles (p. ex. sur une échelle/un toit ou sur des pentes)	☐	☐	☐	☐	☐	☐	☐	☐	☐	☐
d	Rester assis.e pendant une longue durée (4 heures ou plus d'affilée)	☐	☐	☐	☐	☐	☐	☐	☐	☐	☐
e	....	☐	☐	☐	☐	☐	☐	☐	☐	☐	☐

7.2	**Risques chimiques:**<br>**Au cours des 12 derniers mois, à quelle fréquence en moyenne avez-vous exercé les activités suivantes (a-d) dans votre travail agricole quotidien?**<br>**Indépendamment de la fréquence à laquelle vous les avez exercées, quel est le degré de nocivité de chacune d'entre elles à vos yeux?**

		Fréquence						Nocivité pour la santé			
		Jamais	Rarement	Occasionnelle-ment	Souvent	Toujours	Non pertinent	Pas nocif pour la santé	Légèrement	Assez	Très nocif pour la santé
a	Épandage d'engrais	☐	☐	☐	☐	☐	☐	☐	☐	☐	☐
b	Mélange et/ou application de produits phytosanitaires (p. ex. herbicides, fongicides ou insecticides)	☐	☐	☐	☐	☐	☐	☐	☐	☐	☐
c	Activités impliquant un contact avec des gaz ou des fumées (p. ex. de lisier)	☐	☐	☐	☐	☐	☐	☐	☐	☐	☐
d	Utilisation de produits de nettoyage corrosifs (p. ex. nettoyant pour machines agricoles)	☐	☐	☐	☐	☐	☐	☐	☐	☐	☐
e	....	☐	☐	☐	☐	☐	☐	☐	☐	☐	☐

7.3	**Risques biologiques:**<br>**Au cours des 12 derniers mois, à quelle fréquence en moyenne avez-vous exercé les activités suivantes (a-d) dans votre travail agricole quotidien?**<br>**Indépendamment de la fréquence à laquelle vous les avez exercées, quel est le degré de nocivité de chacune d'entre elles à vos yeux?**

**Des travaux où vous...**		Fréquence						Nocivité pour la santé			
		Jamais	Rarement	Occasionnelle-ment	Souvent	Toujours	Non pertinent	Pas de nocif pour la santé	Légèrement	Assez	Très nocif pour la santé
a	entrez en contact avec du pollen/des spores (p. ex. pollen de graminées ou spores de champignons)	☐	☐	☐	☐	☐	☐	☐	☐	☐	☐
b	entrez en contact avec de la poussière (p. ex. poussière de bois ou de foin)	☐	☐	☐	☐	☐	☐	☐	☐	☐	☐
c	entrez en contact avec des animaux ou des excréments d'animaux (p. ex. contact sans clôture/attache des animaux ou par du fumier/sang)	☐	☐	☐	☐	☐	☐	☐	☐	☐	☐
d	pouvez être piqué.e.s ou mordu.e.s par des insectes (p. ex. abeilles ou tiques)	☐	☐	☐	☐	☐	☐	☐	☐	☐	☐
e	....	☐	☐	☐	☐	☐	☐	☐	☐	☐	☐

7.4	**Risques psychosociaux:**<br>**Au cours des 12 derniers mois, à quelle fréquence en moyenne avez-vous été exposé.e aux risques psychosociaux suivants (a-d) dans votre travail agricole quotidien?**<br>**Indépendamment de la fréquence à laquelle vous avez été personnellement exposé.e: Dans quelle mesure considérez-vous que ces différents risques sont dangereux pour la santé?**

		Fréquence						Nocivité pour la santé			
		Jamais	Rarement	Occasionnelle-ment	Souvent	Toujours	Non pertinent	Pas nocif pour la santé	Légèrement	Assez	Très nocif pour la santé
a	Conflits (p. ex. avec des collaborateurs/trices, dans la famille ou le voisinage)	☐	☐	☐	☐	☐	☐	☐	☐	☐	☐
b	Problèmes de sommeil	☐	☐	☐	☐	☐	☐	☐	☐	☐	☐
c	Stress/pression	☐	☐	☐	☐	☐	☐	☐	☐	☐	☐
d	Solitude	☐	☐	☐	☐	☐	☐	☐	☐	☐	☐
e	....	☐	☐	☐	☐	☐	☐	☐	☐	☐	☐

7.5	**Risques liés à l'environnement:**<br>**Au cours des 12 derniers mois, à quelle fréquence en moyenne avez-vous été exposé.e aux risques environnementaux suivants (a-d) dans votre activité agricole quotidienne ?**<br>**Indépendamment de la fréquence à laquelle vous avez été personnellement exposé.e: Dans quelle mesure considérez-vous que les différents risques sont dangereux pour la santé ?**

		Fréquence						Nocivité pour la santé			
		Jamais	Rarement	Occasionnelle-ment	Souvent	Toujours	Non pertinent	Pas de nocif pour la santé	Légèrement	Assez	Très nocif pour la santé
a	Travailler à l'extérieur en plein soleil	☐	☐	☐	☐	☐	☐	☐	☐	☐	☐
b	Tempêtes (p. ex. grêle, foudre ou précipitations abondantes)	☐	☐	☐	☐	☐	☐	☐	☐	☐	☐
c	Glissements de terrain/avalanches	☐	☐	☐	☐	☐	☐	☐	☐	☐	☐
d	Bruit	☐	☐	☐	☐	☐	☐	☐	☐	☐	☐
e	....	☐	☐	☐	☐	☐	☐	☐	☐	☐	☐

Qualité de vie, sommeil et stress

8.1	**Vous trouverez ci-dessous 12 questions et affirmations (a-l). Veuillez répondre à chaque question sur une échelle de 0 à 10.** Veuillez ne cocher qu'une seule réponse pour chaque question/affirmation (a-l).

		0 = Pas du tout satisfait.e										*10 = Entièrement satisfait.e*
a	D'une manière générale, à quel point êtes-vous satisfait.e de votre vie?	0 ☐	1 ☐	2 ☐	3 ☐	4 ☐	5 ☐	6 ☐	7 ☐	8 ☐	9 ☐	10 ☐
		0 = Pas heureux/se du tout										*10 = Très heureux/se*
b	D'une manière générale, à quel point vous sentez-vous heureux/se ou malheureux/se?	0 ☐	1 ☐	2 ☐	3 ☐	4 ☐	5 ☐	6 ☐	7 ☐	8 ☐	9 ☐	10 ☐
		0 = Mauvaise										*10 = Excellente*
c	D'une manière générale, comment évaluez-vous votre santé physique?	0 ☐	1 ☐	2 ☐	3 ☐	4 ☐	5 ☐	6 ☐	7 ☐	8 ☐	9 ☐	10 ☐
		0 = Mauvaise										*10 = Excellente*
d	D'une manière générale, comment évaluez-vous votre santé mentale?	0 ☐	1 ☐	2 ☐	3 ☐	4 ☐	5 ☐	6 ☐	7 ☐	8 ☐	9 ☐	10 ☐
		0 = Pas du tout gratifiant										*10 = Très gratifiant*
e	D'une manière générale, dans quelle mesure avez-vous le sentiment que ce que vous faites dans la vie est utile et gratifiant?	0 ☐	1 ☐	2 ☐	3 ☐	4 ☐	5 ☐	6 ☐	7 ☐	8 ☐	9 ☐	10 ☐
		0 = Pas du tout d'accord										*10 = Complètement d'accord*
f	Dans quelle mesure êtes-vous d'accord avec l'affirmation suivante: "Je sais quel est le sens de ma vie"?	0 ☐	1 ☐	2 ☐	3 ☐	4 ☐	5 ☐	6 ☐	7 ☐	8 ☐	9 ☐	10 ☐

		0	1	2	3	4	5	6	7	8	9	10
		0 = Ne s'applique pas à moi										*10 = S'applique complètement à moi*
g	Dans quelle mesure l'affirmation suivante s'applique-t-elle à vous: "J'agis toujours de manière à promouvoir le bien dans tous les cas, même dans des situations difficiles et stimulantes"?	☐	☐	☐	☐	☐	☐	☐	☐	☐	☐	☐
		0 = Ne s'applique pas à moi										*10 = S'applique complètement à moi*
h	Dans quelle mesure l'affirmation suivante s'applique-t-elle à vous: "Je peux renoncer à une partie de mon bonheur aujourd'hui si, grâce à ce renoncement, mon bonheur sera d'autant plus grand plus tard"?	☐	☐	☐	☐	☐	☐	☐	☐	☐	☐	☐
		0 = Pas du tout d'accord										*10 = Complètement d'accord*
i	Dans quelle mesure êtes-vous d'accord avec l'affirmation suivante: "Je suis satisfait.e des amitiés et des relations que j'ai"?	☐	☐	☐	☐	☐	☐	☐	☐	☐	☐	☐
		0 = Pas du tout d'accord										*10 = Complètement d'accord*
j	Dans quelle mesure êtes-vous d'accord avec l'affirmation suivante: "Mes relations sont aussi satisfaisantes que je le souhaiterais"?	☐	☐	☐	☐	☐	☐	☐	☐	☐	☐	☐
		0 = Je m'inquiète toujours										*10 = Je ne m'inquiète jamais*
k	À quelle fréquence vous inquiétez-vous de ne pas pouvoir faire face aux dépenses mensuelles normales de la vie?	☐	☐	☐	☐	☐	☐	☐	☐	☐	☐	☐
		0 = Je m'inquiète toujours										*10 = Je ne m'inquiète jamais*
l	À quelle fréquence vous faites-vous du souci en matière de sécurité, d'alimentation ou de logement?	☐	☐	☐	☐	☐	☐	☐	☐	☐	☐	☐

8.2	**Combien d'heures par nuit avez-vous dormi en moyenne au cours des 12 derniers mois ? Cela ne doit pas correspondre au nombre d'heures que vous avez passées au lit.** Veuillez indiquer les heures entières.

a Saison froide	☐☐	par nuit
b Saison chaude	☐☐	par nuit

8.3	**Dans l'ensemble, comment évalueriez-vous la qualité de votre sommeil au cours des 12 derniers mois ?** Veuillez ne cocher qu'une seule réponse.

	Très bonne	Bonne	Moyenne	Mauvaise	Très mauvaise
a Saison froide	☐	☐	☐	☐	☐
b Saison chaude	☐	☐	☐	☐	☐

8.4	**Au cours des 12 derniers mois, combien de fois vous est-il arrivé de...** Veuillez ne cocher qu'une seule réponse pour chaque question (a-d).

	Jamais	Rarement	Quelquefois	Souvent
a Avoir du mal à vous endormir?	☐	☐	☐	☐
b Avoir un sommeil agité?	☐	☐	☐	☐
c Vous réveiller plusieurs fois durant la nuit?	☐	☐	☐	☐
d Vous réveiller trop tôt le matin ?	☐	☐	☐	☐

8.5	**Sur une échelle de 1 à 6, comment évalueriez-vous votre capacité à gérer le stress ? (1 = je peux me débarrasser du stress, 6 = le stress me ronge)** Veuillez ne cocher qu'une seule réponse.

Je peux me débarrasser du stress 1	2	3	4	5	Le stress me ronge 6
☐	☐	☐	☐	☐	☐

8.6	**Au cours des 12 derniers mois, comment évalueriez-vous votre niveau de stress dans votre vie (à la maison et au travail) ?** **(1 = pas de stress, 6 = stress extrême)** Veuillez ne cocher qu'une seule réponse.					
	Pas de stress 1	2	3	4	5	Stress extrême 6
a Saison froide	☐	☐	☐	☐	☐	☐
b Saison chaude	☐	☐	☐	☐	☐	☐

8.7	**Comment les aspects suivants de votre santé ont-ils globalement évolué pendant la pandémie de coronavirus (depuis mars 2020)?** Veuillez ne cocher qu'une seule réponse pour chaque aspect (a-e).				
	Bien pire	Plutôt pire	Pas du tout changé	Plutôt mieux	Bien mieux
a Santé physique	☐	☐	☐	☐	☐
b Santé mentale	☐	☐	☐	☐	☐
c Qualité de vie	☐	☐	☐	☐	☐
d Qualité du sommeil	☐	☐	☐	☐	☐
e Mode de vie (alimentation, activité physique, consommation d'alcool/de tabac)	☐	☐	☐	☐	☐

Satisfaction au travail

9.1

Au cours des 12 derniers mois, dans quelle mesure avez-vous été globalement satisfait.e de votre travail sur l'exploitation agricole? Veuillez ne cocher qu'une seule réponse.

➡ Si vous ne travaillez pas dans une exploitation agricole, veuillez passer directement à la question 9.1.1.

- ☐ Très satisfait.e
- ☐ Plutôt satisfait.e
- ☐ En partie satisfait.e
- ☐ Plutôt insatisfait.e
- ☐ Très insatisfait.e

9.1.1	**Si vous ne travaillez pas dans une exploitation agricole: Au cours des 12 derniers mois, dans quelle mesure avez-vous été globalement satisfait.e de votre travail?** Veuillez ne cocher qu'une seule réponse.

- ☐ Très satisfait.e
- ☐ Plutôt satisfait.e
- ☐ En partie satisfait.e
- ☐ Plutôt insatisfait.e
- ☐ Très insatisfait.e

9.2	**Indépendamment du fait qu'il s'agissait d'un travail rémunéré ou non (p. ex. travail ménager), à quelle fréquence avez-vous travaillé au cours des 12 derniers mois...**

**Saison froide**	Jamais	Rarement	Quelquefois	Souvent
a Plus de 5 jours par semaine ?	☐	☐	☐	☐
b Plus de 10 heures par jour ?	☐	☐	☐	☐
**Saison chaude**				
c Plus de 5 jours par semaine ?	☐	☐	☐	☐
d Plus de 10 heures par jour ?	☐	☐	☐	☐

9.3	**Parmi les éléments suivants, quelles sont les <u>3 principales raisons</u> qui vous ont poussé à choisir votre métier d'agriculteur/trice?** Veuillez citer trois éléments parmi les propositions suiviantes.<br>➡ Si vous ne travaillez pas dans une exploitation agricole, veuillez passer directement au chapitre "Maladies et accidents".

- ☐ Sécurité financière
- ☐ Liberté d'organiser son travail de manière autonome
- ☐ Sens de la profession
- ☐ Relations sociales avec les collaborateurs/trices, les connaissances, la famille et/ou les voisin.e.s
- ☐ Horaires de travail flexibles
- ☐ Reconnaissance sociale
- ☐ Travail physique/travail en plein air dans la nature
- ☐ Sécurité de l'emploi
- ☐ Autres, précisez: ....
- ☐ Ne sais pas

9.4	**Supposons que vous décidiez d'abandonner votre métier d'agriculteur/trice: Parmi les éléments suivants, quelles seraient pour vous les <u>3 raisons les plus probables</u> d'abandonner votre métier d'agriculteur/trice?** Veuillez citer 3 eléments.

- ☐ Pression financière
- ☐ Trop peu de temps libre/vacances
- ☐ Manque de reconnaissance sociale
- ☐ Solitude
- ☐ Litiges au sein de l'entreprise, de la famille ou du voisinage
- ☐ Pression politique ou sociale
- ☐ Problèmes de santé
- ☐ Horaires de travail irréguliers/longs
- ☐ Difficultés à trouver une personne pour reprendre l'exploitation
- ☐ Autres, précisez: ....
- ☐ Ne sais pas

Maladies et accidents

Toutes les informations seront traitées de manière strictement confidentielle.

10.1	**Un médecin vous a-t-il déjà dit que vous souffriez de l'une des maladies suivantes ?** Veuillez ne cocher qu'une seule réponse pour chaque maladie (a-l). Si vous avez un diagnostic médical pour une maladie, veuillez également répondre à la question dans les cases grisées.			
			En quelle année environ cela a-t-il été diagnostiqué pour la première fois ?	Prenez-vous régulièrement des médicaments prescrits par votre médecin pour cette maladie/ce problème?
a	Maux de dos pendant 3 mois ou plus, et ce presque tous les jours	☐ Oui → ☐ Non ☐ Ne sais pas ☐ Aucune réponse	☐☐☐☐	☐ Oui ☐ Non
b	Arthrose ou usure des articulations	☐ Oui → ☐ Non ☐ Ne sais pas ☐ Aucune réponse	☐☐☐☐	☐ Oui ☐ Non
c	Asthme	☐ Oui → ☐ Non ☐ Ne sais pas ☐ Aucune réponse	☐☐☐☐	☐ Oui ☐ Non

d	Bronchite chronique ou broncho-pneumopathie chronique obstructive (BPCO)	☐ Oui →<br>☐ Non<br>☐ Ne sais pas<br>☐ Aucune réponse	☐☐☐☐	☐ Oui ☐ Non
e	Allergies (rhume des foins, allergie alimentaire, eczéma, allergie aux venins d'insectes, etc.)	☐ Oui →<br>☐ Non<br>☐ Ne sais pas<br>☐ Aucune réponse	☐☐☐☐	☐ Oui ☐ Non
f	Maladies cardiovasculaires (infarctus du myocarde, insuffisance cardiaque, troubles du rythme cardiaque, etc.)	☐ Oui →<br>☐ Non<br>☐ Ne sais pas<br>☐ Aucune réponse	☐☐☐☐	☐ Oui ☐ Non
g	Diabète ou diabète sucré<br>(y compris le diagnostic du diabète pendant la grossesse)	☐ Oui →<br>☐ Non<br>☐ Ne sais pas<br>☐ Aucune réponse	☐☐☐☐	☐ Oui ☐ Non
h	Cancer (Si oui: quel type de cancer?<br>..)	☐ Oui →<br>☐ Non<br>☐ Ne sais pas<br>☐ Aucune réponse	☐☐☐☐	☐ Oui ☐ Non

i	Attaque cérébrale	☐ Oui → ☐ Non ☐ Ne sais pas ☐ Aucune réponse	☐☐☐☐	☐ Oui ☐ Non
j	Syndrome de Parkinson	☐ Oui → ☐ Non ☐ Ne sais pas ☐ Aucune réponse	☐☐☐☐	☐ Oui ☐ Non
k	Dépression ou troubles anxieux	☐ Oui → ☐ Non ☐ Ne sais pas ☐ Aucune réponse	☐☐☐☐	☐ Oui ☐ Non
l	Accident/blessure/chute avec visite chez le médecin (Si oui: cela s'est-il passé au travail ou pendant les loisirs? ☐ travail ☐ loisirs)	☐ Oui → ☐ Non ☐ Ne sais pas ☐ Aucune réponse	☐☐☐☐	☐ Oui ☐ Non

10.2	Au cours des 12 derniers mois, combien de fois êtes-vous allé.e dans les lieux suivants? **Un transfert d'un hôpital à un autre compte comme une fois. Si vous n'êtes pas allé.e dans l'un de ces lieux au cours des 12 derniers mois, veuillez inscrire le chiffre 0.**
a	☐ Chez le médecin/médecin de famille pour un traitement ambulatoire<br>☐☐ Fois ☐ Ne sais pas
b	☐ A l'hôpital/dans une clinique spécialisée **(sans nuitée)**<br>☐☐ Fois ☐ Ne sais pas
c	☐ A l'hôpital/dans une clinique spécialisée **(avec nuitée)**<br>☐☐ Fois ☐ Ne sais pas
d	☐ Dans un service d'urgences médicales<br>☐☐ Fois ☐ Ne sais pas
e	☐ Autre, veuillez précisez: ....<br>☐☐ Fois

Question finale

11.1	**Pouvons-nous vous recontacter pour une future enquête ou étude sur la santé (par ex. un questionnaire de santé de suivi)?**
	☐ Oui ☐ Non

Votre avis nous intéresse

12.1	Si vous avez encore quelque chose à nous dire, vous trouverez ici un espace pour vos suggestions, souhaits, commentaires ou critiques. Votre opinion nous est précieuse.

Merci beaucoup pour vos réponses et vos réactions!
Nous vous sommes très reconnaissant.e.s pour votre participation.
Votre équipe Swiss TPH

S6 Questionnaire in Italian

Swiss TPH
Swiss Tropical and Public Health Institute
Schweizerisches Tropen- und Public Health-Institut
Institut Tropical et de Santé Publique Suisse

Assoziiertes Institut der Universität Basel

FarmCoSwiss: Questionario sulla salute delle persone attive nell'agricoltura svizzera e dei loro partner

Caro partecipante, cara partecipante

L'Istituto svizzero di salute pubblica e tropicale (Swiss TPH) utilizza questo questionario per indagare sulla **salute e il benessere** generale **delle persone attive nell'agricoltura svizzera e del loro partner o coniuge (di seguito indicato per semplicità come "partner")**. Partecipando, Lei dà un contributo importante alla valutazione e al miglioramento della salute della popolazione agricola in Svizzera. Si prega di completare il questionario senza interruzioni. La compilazione del questionario richiede circa 40 minuti.

Prendiamo molto sul serio il rispetto della protezione dei dati e della riservatezza. Tutte le informazioni raccolte sono quindi trattate in modo **strettamente confidenziale**, analizzate in **forma criptata** e utilizzate esclusivamente per scopi scientifici.

A seconda che

a) si lavora nel settore agricolo o
b) si lavora al di fuori dell'agricoltura, ma il Suo partner lavora nell'agricoltura, risponderà ad altre domande del questionario.

Le domande che possono essere saltate per alcune persone sono chiaramente indicate da una freccia. ➡

Se ha domande o se qualcosa non è chiaro, può contattare il team che si occupa dello studio al numero 061 284 89 29 o scrivendo un' email a farmcoswiss@swisstph.ch.

Grazie mille per la Sua partecipazione!

Prof. Dr Nicole Probst-Hensch

Questionario cartaceo

Si prega di compilare il questionario con una penna biro nera o blu.
Si prega di inserire il questionario compilato nella busta preaffrancata e preindirizzata allegata e di inviarlo all'Istituto Svizzero di salute pubblica e tropicale.

Grazie mille!

Domande introduttive

0	**Si prega di indicare la data in cui si sta completando il questionario.**

☐☐ ☐☐ ☐☐☐☐

Giorno Mese Anno

0.1	**Lavora in una azienda agricola?** Per "lavorare" si intende le persone che svolgono un'attività autonoma, un lavoro dipendente (a tempo pieno o parziale) o una formazione, oppure che svolgono un lavoro stagionale o un aiuto volontario in un'azienda agricola (per esempio come familiari o partner). Le attività in l'azienda agricola possono essere di natura agricola o amministrativa.<br>➡ Se no: Passi direttamente alla domanda 0.2.

☐ Sì ☐ No

0.1.1	**Se Lei lavora in un'azienda agricola: Partecipano al questionario anche altre persone che lavorano nella Sua azienda agricola (tranne il Suo partner)? Se sì: Quante persone?**

☐ Sì ☐ No ☐ Non so

Se sì: Numero di persone: …………………..

0.2	**Anche il Suo partner partecipa a questo questionario?**

☐ Sì ☐ No ☐ Non so ☐ Non ho un partner.

0.2.1	**Se anche il Suo partner partecipa al questionario: Indichi il nome, il cognome e la data di nascita del Suo partner.**<br>Abbiamo bisogno di queste informazioni per poter abbinare Lei con il Suo partner.<br><br>➡ Se il Suo partner non partecipa, Lei non sa o Lei non ha un partner: Passi direttamente al capitolo "Informazioni di contatto.

Nome: ……………………………………………………………………………………………

Cognome: ……………………………………………………………………………………………

Data di nascita: ☐☐ ☐☐ ☐☐☐☐

Giorno Mese Anno

In questo questionario, alcune si riferiscono all'azienda in cui lavora. Per favore, risponda a queste domande per l'azienda agricola in cui ha lavorato per la <u>maggior parte del tempo negli ultimi 12 mesi.</u> Se non lavora nel settore agricolo, risponda a queste domande per l'azienda in cui il Suo partner ha lavorato la maggior parte del tempo negli ultimi 12 mesi. Tutte le informazioni saranno tenute strettamente confidenziali.

Informazioni di contatto

1.1	**Inserisca il Suo nome e cognome.**

Nome: ………………………………………………………………………………………

Cognome: ………………………………………………………………………………………

1.2	**Il Suo indirizzo di casa e/o dell'azienda agricola che ha fornito al momento della registrazione sono cambiati da allora?**

☐ Si

Nuovo indirizzo di casa: ……………………………………………………………..

Da quando è valido questo nuovo indirizzo di casa? (GG/MM/AAAA) ……………………………………………………………..

Nuovo indirizzo dell'azienda agricola: ……………………………………………………………..

Da quando è valido questo nuovo indirizzo dell'azienda agricola? (GG/MM/AAAA) ……………………………………………………………..

☐ No

1.3	**Il Suo numero di telefono e/o il Suo indirizzo e-mail che ha fornito al momento della registrazione sono cambiati da allora?**

☐ Sì

Nuovo numero di telefono: ....

Nuovo indirizzo e-mail: ....

☐ No

Dettagli professionali

2.1	**Qual è il Suo titolo di studio più alto?**<br>Si prega di barrare una sola risposta.

☐ Scuola dell'obbligo (scuole elementari e scuole medie)

☐ Apprendistato/Scuola professionale/Scuola di commercio

☐ Liceo

☐ Scuola tecnica superiore o professionale

☐ Università/Scuola universitaria

☐ Altro, si prega di specificare: ....

2.2	**Qual è la Sua formazione agricola di base?** Sono possibili più risposte.

- ☐ Nessuna formazione agricola di base
- ☐ Tirocinante agricolo (EBA)
- ☐ Agricoltore/Agricoltrice
- ☐ Avicoltore/Avicoltrice
- ☐ Orticoltore/Orticoltrice
- ☐ Frutticoltore/Frutticoltrice
- ☐ Viticoltore/Viticoltrice
- ☐ Cantiniere/Cantiniera
- ☐ Altro, si prega di specificare: ....

2.3	**Qual è la Sua formazione professionale superiore in agricoltura?** Sono possibili più risposte.

- ☐ Nessuna formazione professionale superiore in agricoltura
- ☐ Contadina/Responsabile dell'economia domestica rurale
- ☐ Esame professionale agrario (scuola per capo d'azienda)
- ☐ Esame professionale superiore/Maestria (scuola per capo d'azienda 2)
- ☐ Scuola specializzata superiore: Commerciante agrario/a
- ☐ Scuole specializzata superiore: Agrotecnico/a
- ☐ Scuola specializzata superiore: Tecnico/a vitivinicolo/a
- ☐ Altro, si prega di specificare: ....

2.4	**Quanti anni della Sua vita in totale ha <u>lavorato</u> in un'azienda agricola?** Si prega di barrare una sola risposta.

Mai	1-5 anni	6-10 anni	11-20 anni	21-30 anni	Più di 30 anni
☐	☐	☐	☐	☐	☐

2.5	**Quanti anni della Sua vita in totale ha <u>vissuto</u> in un'azienda agricola?** Si prega di barrare una sola risposta.

Mai	1- 5 anni	6-10 anni	11-20 anni	21-30 anni	Più di 30 anni
☐	☐	☐	☐	☐	☐

2.6	**È cresciuto/a in un'azienda agricola?** Si prega di barrare una sola risposta.	
Sì	No	Altro, si prega di specificare:
☐	☐	☐ ....

Attività lavorativa

3.1	**In quale posizione professionale lavora in azienda?** Sono possibili più risposte.<br>➡ Se non lavora in un'azienda agricola, passi direttamente alla domanda 3.1.1.
a	☐ (Co-)manager (gestione operativa)
b	☐ Lavoro in un'azienda a conduzione familiare (con reddito soggetto all'AVS)
c	☐ Lavoro in un'azienda a conduzione familiare (senza reddito soggetto all'AVS)
d	☐ Impiegato/a con una funzione di gestione (dipendente subordinato/a)
e	☐ Impiegato/a senza una funzione di gestione (nessun dipendente subordinato/a)
f	☐ In pensione con attività lavorativa
g	☐ In formazione
h	☐ Altro, si prega di specificare: ....

3.1.1	**Se non lavora in un'azienda agricola: Qual è la Sua attuale situazione lavorativa?**<br>Sono possibili più risposte.<br>➡ Se lavora in un'azienda agricola, passare direttamente alla domanda 3.2.	
a	☐	Lavoratore indipendente
b	☐	Impiegato/a con una funzione di gestione (dipendente subordinato/a)
c	☐	Impiegato/a senza una funzione di gestione (nessun dipendente subordinato/a)
d	☐	In pensione
e	☐	In formazione
f	☐	Donna/Uomo di casa
g	☐	Invalido/a
h	☐	Alla ricerca di un lavoro
i	☐	Altro, si prega di specificare: …………………………………………………………………..

3.2	**Lavora in un'azienda agricola a tempo pieno o part-time?**<br>Un lavoro è considerato un'occupazione principale se supera chiaramente le altre attività lavorative in termini di **importanza economica** e di **tempo dedicato.** Si prega di barrare una sola risposta.<br>➡ Se non lavora in un'azienda agricola, passi direttamente alla domanda 3.2.1.
	☐ A tempo pieno
	☐ Part-time
	☐ Altro, si prega di specificare: ....

3.2.1	**Se non si lavora in un'azienda agricola: Lavora a tempo pieno o part-time?**<br>Si prega di barrare una sola risposta.<br>➡ Se lavora in un'azienda agricola, passi direttamente alla domanda 3.4.
	☐ A tempo pieno
	☐ Part-time
	☐ Altro, si prega di specificare: ………………………………………………………………

3.3	**Se non lavora in un'azienda agricola: In quale settore economico lavora attualmente?** Sono possibili più risposte.<br>➡ Se lavora in un'azienda agricola, passi direttamente alla domanda 3.4.

- a ☐ Produzione (ad es. produzione di beni)/Fornitura di energia
- b ☐ Costruzione
- c ☐ Commercio e riparazioni/Trasporto e magazzinaggio/Ospitalità
- d ☐ Informazione e comunicazione
- e ☐ Settore del credito e delle assicurazioni
- f ☐ Immobili/Altri servizi economici
- g ☐ Lavoro indipendente/Servizi amministrativi, tecnici o scientifici
- h ☐ Pubblica amministrazione/Educazione/Sanità e lavoro sociale
- i ☐ Arte/Intrattenimento/Altri servizi
- j ☐ Altro, si prega di specificare: ……………………………………………………………

3.4	**Svolge anche una professione al di fuori dell'agricoltura**?<br>➡ Se non lavora in un'azienda agricola, passi direttamente al capitolo "Salute generale" .

- ☐ No
- ☐ Sì, tutto l'anno e meno del 50% dell’orario lavorativo
- ☐ Sì, tutto l'anno e il 50% o più dell’orario lavorativo
- ☐ Sì, solo nella stagione **più fredda** e **meno del 50%** dell’orario lavorativo
- ☐ Sì, solo nella stagione **più fredda** e per **il 50% o più** dell’orario lavorativo
- ☐ Sì, solo nella stagione **più calda** e **meno del 50%** dell’orario lavorativo
- ☐ Sì, solo nella stagione **più calda** e per **il 50% o più** dell’orario lavorativo
- ☐ Altro, si prega di specificare: ....

Se sì, per favore indichi il titolo della Sua professione al di fuori dell'agricoltura.

Titolo di lavoro: ……………………………………………………………………………...

➡ **Se non lavora in un'azienda agricola, salti tutte le domande del capitolo "Gestione dell'azienda agricola e struttura della famiglia" e passi direttamente al capitolo "Salute generale".**

Gestione dell'azienda agricola e struttura della famiglia

4.1 Chi è il/la proprietario/a dell'azienda agricola?
Sono possibili più risposte.

- ☐ Lei stesso/a
- ☐ Suo/Sua partner
- ☐ Suo/Sua padre/madre
- ☐ Suo/Sua fratello/sorella
- ☐ Altro membro della famiglia
- ☐ Persona non parente
- ☐ Altro, si prega di specificare: ....

4.2 Qual è l'orientamento commerciale dell'azienda?
Sono possibili più risposte.

- ☐ Allevamento di bestiame da pascolo - allevamento di bestiame da latte
- ☐ Allevamento di bestiame da pascolo - produzione di carne
- ☐ Allevamento di perfezionamento - allevamento di maiali
- ☐ Allevamento di perfezionamento - allevamento di pollame
- ☐ Azienda dedicata alla campicoltura
- ☐ Azienda agricola con colture speciali (per esempio frutta, verdura, bacche o viti)
- ☐ Attività di orticoltura
- ☐ Azienda dedicata alla selvicoltura
- ☐ Altro, si prega di specificare: ....

4.3	**Quali colture sono coltivate o gestite nell'azienda e approssimativamente quanti ettari per coltura? Se non c'è produzione agricola nell'azienda, si prega di saltare questa domanda.** Sono possibili più risposte.

	**Ettari**				
	Meno di 5	5-10	11-20	21-50	Più di 50
☐ Cereali	☐	☐	☐	☐	☐
☐ Legumi	☐	☐	☐	☐	☐
☐ Semi oleosi	☐	☐	☐	☐	☐
☐ Barbabietole da zucchero/ Barbabietola da foraggio/Patate	☐	☐	☐	☐	☐
☐ Verdura	☐	☐	☐	☐	☐
☐ Frutta/Frutta a nocciolo	☐	☐	☐	☐	☐
☐ Bacche	☐	☐	☐	☐	☐
☐ Viti	☐	☐	☐	☐	☐
☐ Selve	☐	☐	☐	☐	☐
☐ Altro, si prega di specificare:	☐	☐	☐	☐	☐
....					

4.4	**Qual è la dimensione totale approssimativa dell’azienda agricola?** Si prega di barrare una sola risposta.

- ☐ Più piccola di 5 ettari
- ☐ 5-10 ettari
- ☐ 11-20 ettari
- ☐ 21-50 ettari
- ☐ Più grande di 50 ettari

4.5	**Quale metodo di produzione viene utilizzato nell’azienda agricola?** Sono possibili più risposte.

- ☐ Convenzionale
- ☐ Produzione integrata (IP)
- ☐ Biologico
- ☐ Attualmente si sta effettuando una conversione alla produzione biologica
- ☐ Altro, si prega di specificare: ....

4.6	**Da quanti anni viene usata questo metodo di produzione, rispettivamente da quanti anni si trova in un processo di conversione alla produzione biologica?** Sono possibili più risposte.

☐ Convenzionale: ____________________ (in anni interi)

☐ Produzione integrata: ____________________ (in anni interi)

☐ Biologico: ____________________ (in anni interi)

☐ Conversione alla produzione biologica: ____________________ (in anni interi)

☐ Altro: ____________________ (in anni interi)

4.7	**Quali sono le tre principali fonti di reddito dell'azienda agricola? Indichi 3 prodotti o servizi che generano il maggior reddito per l'azienda.** Le risposte possono essere date in qualsiasi ordine.

1 ....

2 ....

3 ....

4.8	**Quante persone vivono permanentemente o a lungo termine nella Sua famiglia, Lei compreso/a?**

Numero di adulti (Lei compreso/a):	☐☐
Numero di persone con meno di 18 anni di età:	☐☐

4.9	**Quante persone lavorano nell’azienda agricola, Lei compreso/a?** Questo include i/le dipendenti a tempo parziale e a tempo pieno, i/le dipendenti stagionali, così come ad esempio i membri della famiglia che lavorano nell’azienda agricola senza retribuzione.

**Stagione più fredda**<br>Numero di adulti (Lei compreso/a):	☐☐☐
**Stagione più calda**<br>Numero di adulti (Lei compreso/a):	☐☐☐

Salute generale

5.1	**In generale, direbbe che la Sua salute è:** Si prega di barrare una sola risposta.

☐ Eccellente ☐ Molto buona ☐ Buona ☐ Passabile ☐ Scadente

5.2	**Le seguenti domande riguardano alcune attività che potrebbe svolgere nel corso di una qualsiasi giornata. Per ognuna di esse, indichi <u>se la Sua salute La limita attualmente nello svolgimento di queste attività</u>.** Si prega di barrare una sola risposta per ogni attività (a-b). Se gli esempi di attività non sono rilevanti per Lei, risponda alla domanda per un'attività con un'intensità fisica simile.

		Sì, mi limita parecchio	Sì, mi limita parzialmente	No, non mi limita per nulla
a	Attività di moderato impegno fisico, come spostare un tavolo, usare l'aspirapolvere o giocare a bocce	☐	☐	☐
b	Salire qualche piano di scale	☐	☐	☐

5.3	**Nelle ultime 4 settimane, quanto spesso ha riscontrato i seguenti problemi sul lavoro o durante altre attività quotidiane al lavoro o a casa, a causa della Sua salute <u>fisica</u>?** Si prega di barrare una sola risposta per ogni affermazione (a-b).

		Sempre	Quasi sempre	Una parte del tempo	Quasi mai	Mai
a	Ho reso meno di quanto avrei voluto.	☐	☐	☐	☐	☐
b	Ho dovuto limitare alcuni tipi di lavoro o di altre attività.	☐	☐	☐	☐	☐

5.4	**Nelle ultime 4 settimane, quanto spesso ha riscontrato i seguenti problemi sul lavoro o durante altre attività quotidiane, a causa del Suo stato emotivo (quale il sentirsi depresso/a o ansioso/a)?** Si prega di barrare una sola risposta per ogni affermazione (a-b).

		Sempre	Quasi sempre	Una parte del tempo	Quasi mai	Mai
a	Ho reso meno di quanto avrei voluto.	☐	☐	☐	☐	☐
b	Ho avuto un calo di concentrazione sul lavoro o in altre attività.	☐	☐	☐	☐	☐

5.5	**Nelle ultime 4 settimane, in che misura il dolore L'ha ostacolato/a nel lavoro che svolge abitualmente (sia in casa sia fuori casa)?**<br>Si prega di barrare una sola risposta.

☐ Per nulla ☐ Molto poco ☐ Un po' ☐ Molto ☐ Moltissimo

5.6	**Le seguenti domande si riferiscono a come si è sentito/a nelle ultime 4 settimane. Risponda a ciascuna domanda scegliendo la risposta che più si avvicina al Suo caso.** Si prega di barrare una sola risposta per ogni affermazione (a-c).

**Per quanto tempo nelle ultime 4 settimane si è sentito/a…**		Sempre	Quasi sempre	Una parte del tempo	Quasi mai	Mai
a	calmo/a e sereno/a?	☐	☐	☐	☐	☐
b	pieno/a di energia?	☐	☐	☐	☐	☐
c	scoraggiato/a e triste?	☐	☐	☐	☐	☐

5.7	**Nelle ultime 4 settimane, per quanto tempo la Sua salute fisica o il Suo stato emotivo hanno interferito nelle Sue attività sociali, in famiglia, con gli amici?**<br>Si prega di barrare una sola risposta.

☐ Sempre ☐ Quasi sempre ☐ Una parte del tempo ☐ Quasi mai ☐ Mai

Attività fisica, alimentazione, alcol e tabacco

6.1	**Approssimativamente quante ore al giorno passa seduto?**		
a	In un giorno di lavoro nella stagione più fredda	☐☐	Ore al giorno
b	In un giorno di riposo nella stagione più fredda	☐☐	Ore al giorno
c	In un giorno di lavoro nella stagione più calda	☐☐	Ore al giorno
c	In un giorno di riposo nella stagione più calda	☐☐	Ore al giorno

6.2 **Quanti giorni alla settimana è fisicamente attivo/a nella Sua occupazione agricola per un totale di 30 minuti o più in modo tale da avere il fiato corto o sudare?**
Si prega di barrare una sola risposta.
➡ Se non lavora in un'azienda agricola, passi direttamente alla domanda 6.2.1.

		Giorni a settimana							
a	Stagione più fredda	0 ☐	1 ☐	2 ☐	3 ☐	4 ☐	5 ☐	6 ☐	7 ☐
b	Stagione più calda	0 ☐	1 ☐	2 ☐	3 ☐	4 ☐	5 ☐	6 ☐	7 ☐

6.2.1	**Se non lavora in un'azienda agricola: Quanti giorni alla settimana è fisicamente attivo/a nella <u>Sua occupazione</u> per un totale di 30 minuti o più in modo tale da avere il fiato corto o sudare?** Si prega di barrare una sola risposta.<br>➡ Se lavora in un'azienda agricola, passi direttamente alla domanda 6.3.

Giorni a settimana

		0	1	2	3	4	5	6	7
a	Stagione più fredda	☐	☐	☐	☐	☐	☐	☐	☐

Giorni a settimana

		0	1	2	3	4	5	6	7
b	Stagione più calda	☐	☐	☐	☐	☐	☐	☐	☐

☐ Non ho una occupazione.

6.3	**Quanti giorni alla settimana è fisicamente attivo/a nel Suo <u>tempo libero</u> per un totale di 30 minuti o più in modo tale da avere il fiato corto o sudare?**<br>Si prega di barrare una sola risposta.

Giorni a settimana

		0	1	2	3	4	5	6	7
a	Stagione più fredda	☐	☐	☐	☐	☐	☐	☐	☐

Giorni a settimana

		0	1	2	3	4	5	6	7
b	Stagione più calda	☐	☐	☐	☐	☐	☐	☐	☐

6.4	**Quanti giorni alla settimana mangia generalmente...?**<br>Si prega di barrare una sola risposta per ogni prodotto (a-d).

				**Giorni a settimana**							
		Mai	Meno frequentement e di un giorno a settimana	1	2	3	4	5	6	7	Non so
a	Carne rossa o salsiccia	☐	☐	☐	☐	☐	☐	☐	☐	☐	☐
b	Verdure cotte	☐	☐	☐	☐	☐	☐	☐	☐	☐	☐
c	Verdure crude o insalata	☐	☐	☐	☐	☐	☐	☐	☐	☐	☐
d	Frutta	☐	☐	☐	☐	☐	☐	☐	☐	☐	☐

6.5	**Quando mangia verdura o frutta, quante prozioni in media al giorno ne consuma?** Una porzione corrisponde a circa una manciata/un grande pomodoro. Si prega di barrare una sola risposta per ogni prodotto (a-c).					
		Meno di 1 porzione	1 Porzion e	2-4 Porzioni	5 porzioni o più	Non so
a	Verdure crude o insalata	☐	☐	☐	☐	☐
b	Verdure cotte	☐	☐	☐	☐	☐
c	Frutta	☐	☐	☐	☐	☐

6.6	**Quanto spesso beve le seguenti bevande alcoliche?** Si prega di barrare una sola risposta per ogni bevanda (a-b).				
		Mai	1-4 volte al mese o meno	Più volte alla settimana	Quotidiana-mente
a	Birra/vino/spumante/mosto fermentato	☐	☐	☐	☐
b	Grappa/Liquori	☐	☐	☐	☐

6.7	**Usa uno dei seguenti prodotti del tabacco?** Si prega di barrare una sola risposta per ogni affermazione (a-c).				
		Mai fumato	Prima, ora non più	Sì, alcuni giorni	Sì, ogni giorno
a	Sigarette	☐	☐	☐	☐
b	Sigarette elettroniche	☐	☐	☐	☐
c	Altri prodotti del tabacco	☐	☐	☐	☐

6.8	**Quanto pesa approssimativamente? Per le donne, se attualmente incinta: all'incirca quanto pensava all'inizio della Sua gravidanza?** Indichi se il peso è una Sua stima o se si è pesato/a da solo/a.
	☐☐☐ kg ☐ stimato ☐ pesato

6.9	**Approssimativamente quanto è alto/a?**
	☐☐☐ cm

➡Se non lavorate in un'azienda agricola, salti tutte le domande del capitolo "La Sua valutazione dei rischi nel lavoro quotidiano" e passi direttamente al capitolo "Qualità della vita, sonno e stress".

La Sua valutazione dei rischi nel lavoro quotidiano

Nella ricerca sulla salute in agricoltura, si fa una distinzione di base tra 5 aree di rischio:

- Fisico (per esempio seduta prolungata, lavoro manuale)
- Chimico (per esempio fertilizzanti, pesticidi, gas e altre sostanze pericolose)
- Biologico (per esempio malattie trasmissibili, polline, polvere)
- Psicosociale (per esempio isolamento sociale, conflitti/discussioni, stress/pressione)
- Ambientale (per esempio tempeste, canicola/freddo, frane)

Vorremmo sapere da Lei:
a) **quanto spesso, in** media, è stato/a esposto/a a singoli rischi delle cinque aree nel Suo **lavoro agricolo quotidiano** negli **ultimi 12 mesi** e
b) **quanto dannoso** considera ogni rischio per la salute, **indipendentemente dalla frequenza con cui è stato/a esposto/a personalmente.**

Per ogni area sono elencate quattro attività (rischi). Nell'ultima riga libera, può scrivere un rischio che pensa debba essere menzionato e spuntare le caselle corrispondenti.

"Mai" significa che tali attività sono svolte nell’azienda agricola ma non da Lei, o che altre persone sono esposte a tali rischi ma non Lei stesso/a.

"Non rilevante" significa che nessuno nell'azienda svolge tali attività/è esposto/a a tali rischi.

7.1 **Rischi fisici:**
In media, quanto spesso ha svolto le seguenti attività (a-d) nella Sua vita agricola quotidiana negli ultimi 12 mesi?
Indipendentemente dalla frequenza con cui le ha eseguite, quanto considera dannosa ogni attività?

		Frequenza						Dannosità per la salute			
		Mai	Raramente	Occasional-mente	Spesso	Sempre	Non rilevante	Non dannoso per la salute	Un po'	Abbastanza	Molto dannoso per la salute
a	Lavoro fisico pesante (per esempio, trasporto di carichi, falciatura manuale)	☐	☐	☐	☐	☐	☐	☐	☐	☐	☐
b	Azionare/Manovrare veicoli/macchine	☐	☐	☐	☐	☐	☐	☐	☐	☐	☐
c	Lavorare in altezza o su terreni difficili (per esempio su una scala/tetto o su pendii)	☐	☐	☐	☐	☐	☐	☐	☐	☐	☐
d	Stare seduti più a lungo (4 o più ore per volta)	☐	☐	☐	☐	☐	☐	☐	☐	☐	☐
e	....	☐	☐	☐	☐	☐	☐	☐	☐	☐	☐

7.2	**Rischi chimici:**<br>**In media, quanto spesso ha svolto le seguenti attività (a-d) nella Sua vita agricola quotidiana negli ultimi 12 mesi?**<br>**Indipendentemente dalla frequenza con cui le ha eseguite, quanto considera dannosa ogni attività?**

		Frequenza						Dannosità per la salute			
		Mai	Raramente	Occasional-mente	Spesso	Sempre	Non rilevante	Non dannoso per la salute	Un po'	Abbastanza	Molto dannoso per la salute
a	Applicazione di fertilizzanti	☐	☐	☐	☐	☐	☐	☐	☐	☐	☐
b	Miscelazione e/o applicazione di prodotti fitosanitari (per esempio erbicidi, fungicidi o insetticidi).	☐	☐	☐	☐	☐	☐	☐	☐	☐	☐
c	Attività in cui si entra in contatto con gas o fumi (per esempio da liquame)	☐	☐	☐	☐	☐	☐	☐	☐	☐	☐
d	Uso di detergenti corrosivi (per esempio prodotto per pulire macchine agricole)	☐	☐	☐	☐	☐	☐	☐	☐	☐	☐
e	....	☐	☐	☐	☐	☐	☐	☐	☐	☐	☐

7.3	**Rischi biologici:** **In media, quanto spesso ha svolto le seguenti attività (a-d) nella Sua vita agricola quotidiana negli ultimi 12 mesi? Indipendentemente dalla frequenza con cui le ha eseguite, quanto considera dannosa ogni attività?**

**Lavori dove...**		Frequenza						Dannosità per la salute			
		Mai	Raramente	Occasional-mente	Spesso	Sempre	Non rilevante	Non dannoso per la salute	Un po'	Abbastanza	Molto dannoso per la salute
a	entra in contatto con polline/spore (per esempio polline d'erba o spore di funghi)	☐	☐	☐	☐	☐	☐	☐	☐	☐	☐
b	entra in contatto con la polvere (per esempio polvere di legno o polvere di fieno).	☐	☐	☐	☐	☐	☐	☐	☐	☐	☐
c	entra in contatto con animali o escrementi di animali (per esempio contatto avvenuto poiché animali non recintati o non legati o attraverso lo sterco/sangue)	☐	☐	☐	☐	☐	☐	☐	☐	☐	☐
d	è punto/a o morso/a da insetti (per esempio api o zecche)	☐	☐	☐	☐	☐	☐	☐	☐	☐	☐
e	....	☐	☐	☐	☐	☐	☐	☐	☐	☐	☐

7.4	**Rischi psicosociali:** **In media, quanto spesso è stato esposto ai seguenti rischi psicosociali (a-d) nella Sua vita agricola quotidiana negli ultimi 12 mesi? Indipendentemente dalla frequenza con cui è stato/a esposto/a personalmente: quanto considera dannoso ciascun rischio?**

		Frequenza						Dannosità per la salute			
		Mai	Raramente	Occasional-mente	Spesso	Sempre	Non rilevante	Non dannoso per la salute	Un po'	Abbastanza	Molto dannoso per la salute
a	Conflitti (ad es. con i/le colleghi/e, in famiglia o nel vicinato)	☐	☐	☐	☐	☐	☐	☐	☐	☐	☐
b	Problemi di sonno	☐	☐	☐	☐	☐	☐	☐	☐	☐	☐
c	Stress/Pressione	☐	☐	☐	☐	☐	☐	☐	☐	☐	☐
d	Solitudine	☐	☐	☐	☐	☐	☐	☐	☐	☐	☐
e	....	☐	☐	☐	☐	☐	☐	☐	☐	☐	☐

7.5	**Rischi ambientali:**<br>**In media, quanto spesso è stato esposto ai seguenti rischi ambientali (a-d) nella Sua vita agricola quotidiana negli ultimi 12 mesi?**<br>**Indipendentemente dalla frequenza con cui è stato/a esposto/a personalmente: quanto considera dannoso ciscun rischio?**

		Frequenza						Dannosità per la salute			
		Mai	Raramente	Occasional-mente	Spesso	Sempre	Non rilevante	Non dannoso per la salute	Un po'	Abbastanza	Molto dannoso per la salute
a	Lavorare fuori sotto il sole cocente	☐	☐	☐	☐	☐	☐	☐	☐	☐	☐
b	Tempeste (ad es. con grandine o molte precipitazioni)	☐	☐	☐	☐	☐	☐	☐	☐	☐	☐
c	Frane/valanghe	☐	☐	☐	☐	☐	☐	☐	☐	☐	☐
d	Rumore	☐	☐	☐	☐	☐	☐	☐	☐	☐	☐
e	....	☐	☐	☐	☐	☐	☐	☐	☐	☐	☐

Qualità della vita, sonno e stress

8.1	**Troverete 12 domande e affermazioni (a-l) qui sotto. Per favore, risponda ad ogni domanda sulla scala da 0 a 10.** Si prega di barrare una sola risposta per ogni domanda/affermazioni (a-l).

		0 = Per niente soddisfatto/a										*10 = Completamente soddisfatto/a*
a	Chiesto in termini generali - quanto è soddisfatto/a della Sua vita?	0 ☐	1 ☐	2 ☐	3 ☐	4 ☐	5 ☐	6 ☐	7 ☐	8 ☐	9 ☐	10 ☐
		0 = Per niente felice										*10 = Completamente felice*
b	Chiesto in termini generali - quanto si sente felice o infelice?	0 ☐	1 ☐	2 ☐	3 ☐	4 ☐	5 ☐	6 ☐	7 ☐	8 ☐	9 ☐	10 ☐
		0 = Pessima										*10 = Eccellente*
c	Chiesto in termini generali - come valuta la Sua salute fisica?	0 ☐	1 ☐	2 ☐	3 ☐	4 ☐	5 ☐	6 ☐	7 ☐	8 ☐	9 ☐	10 ☐
		0 = Pessima										*10 = Eccellente*
d	Chiesto in termini generali - come valuta la Sua salute mentale?	0 ☐	1 ☐	2 ☐	3 ☐	4 ☐	5 ☐	6 ☐	7 ☐	8 ☐	9 ☐	10 ☐
		0 = Per niente gratificante										*10 = Assolutamente gratificante*
e	Domanda in termini generali - fino a che punto sente che ciò che fa nella vita è significativo e gratificante?	0 ☐	1 ☐	2 ☐	3 ☐	4 ☐	5 ☐	6 ☐	7 ☐	8 ☐	9 ☐	10 ☐
		0 = Non sono affatto d'accordo										*10 = Totalmente d'accordo*
f	Quanto è d'accordo con la seguente affermazione: "So qual è il senso della mia vita"?	0 ☐	1 ☐	2 ☐	3 ☐	4 ☐	5 ☐	6 ☐	7 ☐	8 ☐	9 ☐	10 ☐

		0 =<br>Non sono affatto d'accordo										*10 =<br>Totalmente d'accordo*
g	In che misura è d'accordo con la seguente affermazione: "Agisco sempre in modo da promuovere il bene in ogni caso, anche in situazioni difficili e impegnative"?	0 ☐	1 ☐	2 ☐	3 ☐	4 ☐	5 ☐	6 ☐	7 ☐	8 ☐	9 ☐	10 ☐
		0 =<br>Non sono affatto d'accordo										*10 =<br>Totalmente d'accordo*
h	In che misura è d'accordo con la seguente affermazione: "Posso fare a meno di una parte di felicità oggi, se questa rinuncia rende la mia felicità tanto più grande in un secondo momento"?	0 ☐	1 ☐	2 ☐	3 ☐	4 ☐	5 ☐	6 ☐	7 ☐	8 ☐	9 ☐	10 ☐
		0 =<br>Non sono affatto d'accordo										*10 =<br>Totalmente d'accordo*
i	In che misura è d'accordo con la seguente affermazione: "Sono soddisfatto/a delle amicizie e delle relazioni che ho"?	0 ☐	1 ☐	2 ☐	3 ☐	4 ☐	5 ☐	6 ☐	7 ☐	8 ☐	9 ☐	10 ☐
		0 =<br>Non sono affatto d'accordo										*10 =<br>Totalmente d'accordo*
j	In che misura è d'accordo con la seguente affermazione: "Le mie relazioni sono soddisfacenti come vorrei che fossero"?	0 ☐	1 ☐	2 ☐	3 ☐	4 ☐	5 ☐	6 ☐	7 ☐	8 ☐	9 ☐	10 ☐
		0 =<br>Mi preoccupo sempre										*10 =<br>Non mi preoccupo mai*
k	Quanto spesso si preoccupa di non potersi pagare i normali costi mensili della vita?	0 ☐	1 ☐	2 ☐	3 ☐	4 ☐	5 ☐	6 ☐	7 ☐	8 ☐	9 ☐	10 ☐
		0 =<br>Mi preoccupo sempre										*10 =<br>Non mi preoccupo mai*
l	Quanto spesso si preoccupa della sicurezza, del cibo o dell'alloggio?	0 ☐	1 ☐	2 ☐	3 ☐	4 ☐	5 ☐	6 ☐	7 ☐	8 ☐	9 ☐	10 ☐

8.2	**Quante ore per notte ha dormito in media negli ultimi 12 mesi? Questo non deve essere uguale al numero di ore che ha passato a letto.** Indicare le ore intere.

a Stagione più fredda	☐☐	per notte
b Stagione più calda	☐☐	per notte

8.3	**Nel complesso, come valuterebbe la qualità del suo sonno negli ultimi 12 mesi?** Si prega di barrare una sola risposta.

	Molto buona	Buona	Mediocre	Cattiva	Pessima
a Stagione più fredda	☐	☐	☐	☐	☐
b Stagione più calda	☐	☐	☐	☐	☐

8.4	**Quanto spesso le è successo negli ultimi 12 mesi che Lei...** Si prega di barrare una sola risposta per ogni domanda (a-d).

	Mai	Raramente	Occasional mente	Spesso
a avere difficoltà ad addormentarsi?	☐	☐	☐	☐
b avere un sonno agitato?	☐	☐	☐	☐
c svegliarsi più volte durante la notte?	☐	☐	☐	☐
d svegliarsi troppo presto la mattina?	☐	☐	☐	☐

8.5	**Su una scala da 1 a 6, come valuterebbe la Sua capacità di affrontare lo stress? (1 = posso scrollarmi lo stress di dosso, 6 = lo stress mi divora)** Si prega di barrare una sola risposta.

Posso scrollarmi lo stress di dosso 1	2	3	4	5	Lo stress mi divora 6
☐	☐	☐	☐	☐	☐

8.6	**Negli ultimi 12 mesi, quanto valuterebbe il livello di stress nella Sua vita (a casa e al lavoro)?** **(1 = nessuno stress, 6 = stress estremo)** Si prega di barrare una sola risposta.					
	Nessuno stress 1	2	3	4	5	Stress estremo 6
a Stagione più fredda	☐	☐	☐	☐	☐	☐
	Nessuno stress 1	2	3	4	5	Stress estremo 6
b Stagione più calda	☐	☐	☐	☐	☐	☐

8.7	**Come sono cambiati i seguenti aspetti della Sua salute generale durante la pandemia di coronavirus (da marzo 2020)?** Si prega di barrare una sola risposta per ogni affermazione (a-e).				
	Molto peggiorato/a	Piuttosto peggiorato/a	Non è cambiato/a affatto	Piuttosto migliorato/a	Molto migliorato/a
a Salute fisica	☐	☐	☐	☐	☐
b Salute mentale	☐	☐	☐	☐	☐
c Qualità della vita	☐	☐	☐	☐	☐
d Qualità del sonno	☐	☐	☐	☐	☐
e Stile di vita sano (dieta, attività fisica, consumo di alcol/tabacco)	☐	☐	☐	☐	☐

Soddisfazione sul lavoro

9.1 **Negli ultimi 12 mesi, quanto è stato/a complessivamente soddisfatto/a del suo lavoro in l'azienda agricola?** Si prega di barrare una sola risposta.

➡ Se non lavora in un'azienda agricola, passi direttamente alla domanda 9.1.1**.**

- ☐ Molto soddisfatto/a
- ☐ Abbastanza soddisfatto/a
- ☐ Né soddisfatto/a né insoddisfatto/a
- ☐ Abbastanza insoddisfatto/a
- ☐ Molto insoddisfatto/a

9.1.1	**Se non si lavora in un'azienda agricola: Negli ultimi 12 mesi, quanto è stato/a complessivamente soddisfatto/a del suo lavoro?** Si prega di barrare una sola risposta.<br>➡ Se lavora in un'azienda agricola, passi direttamente alla domanda 9.2.

- ☐ Molto soddisfatto/a
- ☐ Abbastanza soddisfatto/a
- ☐ Né soddisfatto/a né insoddisfatto/a
- ☐ Abbastanza insoddisfatto/a
- ☐ Molto insoddisfatto/a

9.2	**Indipendentemente dal fatto che si trattasse di un lavoro retribuito o non retribuito (per esempio lavori domestici), quanto spesso negli ultimi 12 mesi ha lavorato...**

**Stagione più fredda**	Mai	Raramente	Occasional mente	Spesso
a più di 5 giorni a settimana?	☐	☐	☐	☐
b più di 10 ore al giorno?	☐	☐	☐	☐
**Stagione più calda**				
c più di 5 giorni a settimana?	☐	☐	☐	☐
d più di 10 ore al giorno?	☐	☐	☐	☐

9.3	**Quali del seguenti ragioni sono state le 3 ragioni più importanti che l'hanno spinta a scegliere la Sua professione nell'ambito agricolo?** Per favore, indichi 3 ragioni.<br>➡ Se non lavora in un'azienda agricola, passi direttamente al capitolo "Malattie e incidenti".

- ☐ Sicurezza finanziaria
- ☐ Libertà di organizzare il lavoro in modo indipendente
- ☐ Sensatezza della professione
- ☐ Relazioni sociali con colleghi/e, conoscenti, familiari e/o vicini
- ☐ Orario di lavoro flessibile
- ☐ Riconoscimento sociale
- ☐ Lavoro fisico/lavoro fuori nella natura
- ☐ Sicurezza del lavoro
- ☐ Altro, si prega di specificare: ....
- ☐ Non so

9.4	**Supponiamo che Lei decida di abbandonare la Sua professione di agricoltore/agricoltrice: Quali delle seguenti ragionisarebbero le 3 ragioni più probabili che La spingerebbero a lasciare la Sua professione di agricoltore/agricoltrice?** Per favore, indichi 3 ragioni.

- ☐ Pressione finanziaria
- ☐ Troppo poco tempo libero/vacanze
- ☐ Mancanza di riconoscimento sociale
- ☐ Solitudine
- ☐ Controversie sul posto di lavoro, in famiglia o nel quartiere
- ☐ Pressione politica o sociale
- ☐ Problemi di salute
- ☐ Orari di lavoro irregolari/lunghi
- ☐ Problemi a trovare un successore per l'azienda
- ☐ Altro, si prega di specificare: ....
- ☐ Non so

Malattie e incidenti

Tutte le informazioni saranno trattate con la massima riservatezza.

10.1	**Un medico Le ha mai detto di avere una delle seguenti malattie?** Si prega di barrare una sola risposta per ogni malattia (a-l). Se ha una diagnosi medica per una malattia, per favore risponda anche alla domanda nei campi colorati in grigio.			
			Approssimativamente in che anno è stato diagnosticato per la prima volta?	Prende regolarmente le medicine prescritte dal Suo medico per questa malattia/sintomi?
a	Dolore alla schiena per 3 mesi o più e quasi ogni giorno	☐ Si → ☐ No ☐ Non so ☐ Nessuna risposta	☐☐☐☐	☐ Sì ☐ No
b	Artrosi o usura delle articolazioni	☐ Si → ☐ No ☐ Non so ☐ Nessuna risposta	☐☐☐☐	☐ Sì ☐ No
c	Asma	☐ Si → ☐ No ☐ Non so ☐ Nessuna risposta	☐☐☐☐	☐ Sì ☐ No

d	Bronchite cronica o malattia polmonare ostruttiva cronica (COPD)	☐ Si → ☐ No ☐ Non so ☐ Nessuna risposta	☐☐☐☐	☐ Sì ☐ No
e	Allergie (raffreddore da fieno, allergia alimentare, eczema, allergia al veleno degli insetti, ecc.)	☐ Si → ☐ No ☐ Non so ☐ Nessuna risposta	☐☐☐☐	☐ Sì ☐ No
f	Malattie cardiovascolari (infarto, insufficienza cardiaca, aritmie cardiache, ecc.)	☐ Si → ☐ No ☐ Non so ☐ Nessuna risposta	☐☐☐☐	☐ Sì ☐ No
g	Diabete o diabete mellito (incl. diagnosi di diabete durante la gravidanza)	☐ Si → ☐ No ☐ Non so ☐ Nessuna risposta	☐☐☐☐	☐ Sì ☐ No
h	Cancro (Se sì: quale tipo di cancro? ....)	☐ Si → ☐ No ☐ Non so ☐ Nessuna risposta	☐☐☐☐	☐ Sì ☐ No

i	Ictus	☐ Si → ☐ No ☐ Non so ☐ Nessuna risposta	☐☐☐☐	☐ Sì ☐ No
j	Sindrome di Parkinson, chiamata anche paralisi da scuotimento	☐ Si → ☐ No ☐ Non so ☐ Nessuna risposta	☐☐☐☐	☐ Sì ☐ No
k	Depressione o disturbo d'ansia	☐ Si → ☐ No ☐ Non so ☐ Nessuna risposta	☐☐☐☐	☐ Sì ☐ No
l	Incidente/infortunio/caduta con visita medica (se sì: è successo al lavoro o nel tempo libero? ☐ Lavoro ☐ tempo libero)	☐ Si → ☐ No ☐ Non so ☐ Nessuna risposta	☐☐☐☐	☐ Sì ☐ No

10.2	**Quanto spesso è stato/a nei seguenti luoghi negli ultimi 12 mesi?**<br>**Un trasferimento da un ospedale all'altro conta come una volta - Se non è stato/a in nessuno di questi luoghi negli ultimi 12 mesi, scriva il numero 0.**
a	☐ Dal medico/medico di famiglia per un trattamento ambulatoriale<br>☐☐ Volte ☐ Non so
b	☐ In ospedale/clinica specializzata **(senza pernottamento)**<br>☐☐ Volte ☐ Non so
c	☐ In ospedale/clinica specializzata **(con pernottamento)**<br>☐☐ Volte ☐ Non so
d	☐ In un pronto soccorso medico<br>☐☐ Volte ☐ Non so
e	☐ Altro, si prega di specificare: ....<br>☐☐ Volte

Domanda finale

11.1	**Possiamo ricontattarla per un futuro sondaggio o studio sulla salute (ad esempio un questionario di follow-up sulla salute)?**
☐ Sì	☐ No

La Sua opinione è necessaria

12.1	**Se c'è qualcos'altro che vorrebbe dirci, qui troverà spazio per suggerimenti, desideri, commenti o critiche. La Sua opinione è importante per noi.**

Grazie mille per le Sue risposte e il Suo feedback!
Apprezziamo molto la sua partecipazione.
Il vostro team del Swiss TPH

S7 Non-responder questionnaire in German

Formular bei Nicht-Teilnahme

Wenn Sie nicht an der Gesundheitsumfrage teilnehmen möchten, bitten wir Sie, uns trotzdem kurze Fragen zu den Gründen Ihrer Nicht-Teilnahme und zu Ihrer Person zu beantworten (es dauert maximal 5 Minuten) und uns im vorfrankierten Briefumschlag zurückzusenden. Sie können den Fragebogen auch online ausfüllen:

Link: https://redcap.link/farmco.swiss

Passwort:

Ihre Antworten zu den nachfolgenden Fragen benötigen wir, um zukünftige Projekte zu verbessern und um mögliche Verzerrungen der Studienresultate durch Unterschiede zwischen Nicht-Teilnehmenden und Teilnehmenden zu korrigieren.

Zudem können wir Sie anhand Ihrer Angaben aus der Liste der angemeldeten Personen (die Anmeldung erfolgte über unsere Webseite: www.swisstph.ch/farmcoswiss) streichen, wodurch Sie in Zukunft nicht mehr für diese Studie kontaktiert werden. Alle Ihre Angaben werden streng vertraulich behandelt und anonymisiert.

1	**Aus welchem Grund lehnen Sie die Teilnahme an der Gesundheitsstudie ab?** Bitte kreuzen alle Gründe an, die auf Sie zutreffen.
☐	Ich erfülle die Teilnahmebedingungen nicht, nämlich:<br>☐ Ich bin unter 18 Jahre alt. ☐ Weder ich noch mein/e Ehe-/Lebenspartner/in sind in der Schweizer Landwirtschaft tätig. ☐ Ich wohne nicht längerfristig oder permanent in der Schweiz.
☐	Keine Zeit
☐	Krankheit
☐	Kein Interesse oder nicht überzeugt vom Zweck der Studie
☐	Zweifel an Datenschutzmassnahmen
☐	Zu viel Aufwand (Umfrage dauert zu lange)
☐	Ich lehne die Teilnahme an Studien im Allgemeinen ab
☐	Anderes, bitte präzisieren: ....

2	**Was ist Ihr Geschlecht?**
☐	Männlich
☐	Weiblich
☐	Anderes
☐	Keine Angabe

3	**Welches ist Ihr Alter (in ganzen Jahren)?**
☐☐☐	☐ Keine Angabe

4	**Was ist Ihr höchster Bildungsabschluss?**<br>Bitte nur eine Antwort ankreuzen.

- ☐ Obligatorische Primar- und Oberstufe
- ☐ Lehre/Berufsschule/Handelsschule
- ☐ Kantonsschule/Gymnasium
- ☐ Höhere Fach- oder Berufsschule
- ☐ Universität/Hochschule
- ☐ Anderes, bitte präzisieren:

……………………………………………………………………......

Bitte füllen Sie nachfolgende Fragen für den landwirtschaftlichen Betrieb aus, auf welchem Sie die meiste Zeit in den letzten 12 Monaten tätig waren (oder auf welchem Ihr/e Partner/in die meiste Zeit in den letzten 12 Monaten tätig war, falls Sie selbst nicht in der Landwirtschaft arbeiten).

5	**Falls Sie und/oder Ihr/e Partner/in in der Landwirtschaft tätig sind: Was ist die betriebswirtschaftliche Ausrichtung des landwirtschaftlichen Betriebs?** Mehrere Antworten sind möglich.
☐	Weideviehbetrieb
☐	Veredelungsbetrieb (z.B. Schweine- oder Geflügelmast)
☐	Ackerbaubetrieb
☐	Spezialkulturbetrieb (z.B. Obst, Gemüse, Beeren oder Reben)
☐	Gartenbaubetrieb
☐	Forstwirtschaft
☐	Andere, bitte präzisieren: ……………………………………………………………….
☐	Weiss nicht
☐	Keine Angabe

6	**<u>Falls Sie und/oder Ihr/e Partner/in in der Schweizer Landwirtschaft tätig sind:</u>**<br>**Wie gross ist der Betrieb insgesamt ungefähr (in Hektaren)?**<br>Bitte nur eine Antwort ankreuzen.
☐	Kleiner als 5 ha
☐	5-10 ha
☐	11-20 ha
☐	21-50 ha
☐	Grösser als 50 ha
☐	Weiss nicht
☐	Keine Angabe

7	**<u>Falls Sie und/oder Ihr/e Partner/in in der Schweizer Landwirtschaft tätig sind:</u>**<br>**Welche Produktionsform wird auf dem Betrieb angewendet?**<br>Mehrere Antworten sind möglich.
☐	Konventionell
☐	Integrierte Produktion (IP)
☐	Biologisch
☐	Aktuell Umstellung auf biologische Produktion
☐	Andere, bitte präzisieren: ………………………………………………………………..
☐	Weiss nicht
☐	Keine Angabe

8	**<u>Falls Sie und/oder Ihr/e Partner/in in der Schweizer Landwirtschaft tätig sind:</u>**<br>**Wer besitzt den landwirtschaftlichen Betrieb?**<br>Mehrere Antworten sind möglich.	
☐ Sie selbst	☐ Partner/in	☐ Vater/Mutter
☐ Bruder/Schwester	☐ Anderes Familienmitglied	☐ Nicht-verwandte Person
☐ Andere, bitte präzisieren:	…………………………….....	☐ Weiss nicht
☐ Keine Angabe		

9	**Wie würden Sie Ihren Gesundheitszustand im Allgemeinen beschreiben?**<br>Bitte nur eine Antwort ankreuzen.
	☐ Ausgezeichnet ☐ Sehr gut ☐ Gut ☐ Weniger gut ☐ Schlecht

10	**Wie schätzen Sie allgemein Ihre Lebensqualität ein?**<br>Bitte nur eine Antwort ankreuzen.
	☐ Ausgezeichnet ☐ Sehr gut ☐ Gut ☐ Weniger gut ☐ Schlecht

Herzlichen Dank für Ihre Antworten! Wir schätzen diese sehr.
Ihr Swiss TPH Team

S8 Non-responder questionnaire in French

Formulaire en cas de non-participation

Si vous ne souhaitez pas participer à l'enquête sur la santé, nous vous prions de bien vouloir tout de même répondre à de brèves questions sur les raisons de votre non-participation et sur vous-même (cela vous prendra au maximum 5 minutes) et de nous les renvoyer dans l'enveloppe préaffranchie. Vous pouvez également remplir le questionnaire en ligne:

Lien: https://redcap.link/farmco.swiss

Mot de passe:

Nous avons besoin de vos réponses aux questions suivantes pour améliorer les projets futurs et pour corriger les éventuelles distorsions des résultats de l'étude dues aux différences entre les non-participants et les participants.

En outre, vos données nous permettent de vous retirer de la liste des personnes inscrites (l'inscription a été effectuée via notre site web: www.swisstph.ch/farmcoswiss) ce qui signifie que vous ne serez plus contacté pour cette étude. Toutes vos données seront traitées de manière strictement confidentielle et anonyme.

1	**Pour quelle(s) raison(s) refusez-vous de participer à l'étude sur la santé?** Veuillez cocher toutes les raisons qui s'appliquent.
☐	Je ne remplis pas les conditions de participation:<br>☐ J'ai moins de 18 ans. ☐ Ni moi ni mon/ma partenaire ne travaillons dans l'agriculture suisse. ☐ Je n'habite pas en Suisse à long terme ou de manière permanente.
☐	Pas de temps
☐	Maladie
☐	Pas intéressé.e ou pas convaincu.e de l'objectif de l'étude
☐	Doutes sur les mesures de protection des données
☐	Trop de travail (l'enquête prend trop de temps)
☐	Je refuse de participer à des études en général
☐	Autre, veuillez préciser: ………………………………………………………………

2	**Quel est votre genre?**
☐	Homme
☐	Femme
☐	Autre
☐	Aucune réponse

3	**Quel est votre âge? (en années entières)?**
☐☐☐	☐ Aucune réponse

4	**Quel est votre niveau d'études le plus élevé?**<br>Veuillez ne cocher qu'une seule réponse.
☐	Enseignement primaire et secondaire obligatoire
☐	Apprentissage/École professionnelle/École de commerce
☐	École cantonale/Gymnase
☐	École technique ou professionnelle supérieure
☐	Université/Haute école
☐	Autre, veuillez préciser: …………………………………………………………….….....

Veuillez remplir les questions suivantes pour l'exploitation agricole sur laquelle vous avez travaillé la pluspart du temps au cours des 12 derniers mois (ou sur laquelle votre partenaire a travaillé la pluspart du temps au cours des 12 derniers mois si vous ne travaillez pas vous-même dans l'agriculture).

5	**Si vous et/ou votre partenaire travaillez dans l'agriculture: Quelle est l'orientation économique de l'exploitation agricole?** Plusieurs réponses sont possibles.
☐	Elevage de bétail sur pâturage
☐	Exploitation axée sur la transformation (p. ex. porc ou volaille)
☐	Exploitation agricole
☐	Exploitation de cultures spéciales (p. ex. fruits, légumes, baies ou vignes)
☐	Entreprise horticole
☐	Exploitation forestière
☐	Autre, veuillez préciser: …………………………………………………………………..
☐	Je ne sais pas
☐	Aucune réponse

6	**<u>Si vous et/ou votre partenaire travaillez dans l'agriculture:</u> Quelle est la taille approximative de l'exploitation (en hectares)?**<br>Veuillez ne cocher qu'une seule réponse.
☐	Moins de 5 ha
☐	5-10 ha
☐	11-20 ha
☐	21-50 ha
☐	Plus de 50 ha
☐	Je ne sais pas
☐	Aucune réponse

7	**<u>Si vous et/ou votre partenaire travaillez dans l'agriculture:</u> Quelle forme de production est pratiquée sur l'exploitation?**<br>Plusieurs réponses sont possibles.
☐	Conventionnelle
☐	Production intégrée (PI)
☐	Biologique
☐	Actuellement en reconversion vers la production biologique
☐	Autre, veuillez préciser: …………………………………………………………………..
☐	Je ne sais pas
☐	Aucune réponse

8	**<u>Si vous et/ou votre partenaire travaillez dans l'agriculture:</u> Qui est propriétaire de l'exploitation agricole?** Plusieurs réponses sont possibles.

☐ Vous-même	☐ Partenaire	☐ Père/mère
☐ Frère/sœur	☐ Autre membre de la famille	☐ Personne non apparentée
☐ Autre, veuillez préciser:	……………………………....	☐ Je ne sais pas
☐ Aucune réponse		

9	**Dans l'ensemble, pensez-vous que votre santé est:** Veuillez ne cocher qu'une seule réponse.

☐ Excellente ☐ Très bonne ☐ Bonne ☐ Médiocre ☐ Mauvaise

10	**Dans l'ensemble, pensez-vous que votre qualité de vie est:** Veuillez ne cocher qu'une seule réponse.

☐ Excellente ☐ Très bonne ☐ Bonne ☐ Médiocre ☐ Mauvaise

Merci beaucoup pour vos réponses! Nous les apprécions beaucoup.

Votre équipe Swiss TPH

S9 Non-responder questionnaire in Italian

Modulo in caso di non partecipazione

Se non vuole partecipare al sondaggioe sulla salute, Le chiediamo comunque di rispondere a brevi domande sui motivi della Sua non-partecipazione e su di Lei (la compilazione richiede al massimo 5 minuti) e di rispedire il modulo nella busta preaffrancata. È possibile compilare il questionario anche online:

Link: https://redcap.link/farmco.swiss

Password:

Abbiamo bisogno delle Sue risposte alle seguenti domande per migliorare i progetti futuri e per correggere eventuali distorsioni nei risultati dello studio dovute alle differenze tra i non-partecipanti e i partecipanti.

Inoltre, le informazioni fornite ci permetteranno di rimuoverla dall'elenco delle persone che si sono registrate (la registrazione è stata effettuata tramite il nostro sito web: www.swisstph.ch/farmcoswiss), il che significa che Lei non sarà più contattato/a per questo studio. Tutte le Sue informazioni saranno anonimizzate e mantenute strettamente confidenziali.

1	**Per quale motivo/quali motivi rifiuta di partecipare allo studio?**<br>Per favore, selezioni tutti i motivi che si applicano.
☐	Non soddisfo le condizioni di partecipazione:<br>☐ Ho meno di 18 anni. ☐ Né io né il mio partner siamo attivi nell'agricoltura svizzera. ☐ Non vivo permanentemente o a lungo termine in Svizzera.
☐	Non ho tempo
☐	Malattia
☐	Non sono interessato/a o convinto/a dello scopo dello studio
☐	Dubbi sulle misure di protezione dei dati
☐	Troppo impegno (il sondaggio richiede troppo tempo)
☐	In genere rifiuto di partecipare a studi
☐	Altro, si prega di specificare: …………………………………………………………………..

2	**Qual è il Suo genere?**
☐	Maschio
☐	Femmina
☐	Altro
☐	Nessuna risposta

3	**Qual è la Sua età (in anni interi)?**
☐☐☐	☐ Nessuna risposta

4	**Qual è il Suo titolo di studio più alto?**<br>Si prega di barrare una sola risposta.
☐	Scuola dell'obbligo (scuole elementari e scuole medie)
☐	Apprendistato/Scuola professionale/Scuola di commercio
☐	Liceo
☐	Scuola tecnica superiore o professionale
☐	Università/Scuola universitaria
☐	Altro, si prega di specificare:<br>……………………………………………………………….....

La preghiamo di compilare le seguenti domande per l'azienda agricola in cui ha lavorato per la maggior parte del tempo negli ultimi 12 mesi (o in cui ha lavorato il Suo partner per la maggior parte del tempo negli ultimi 12 mesi, se Lei stesso/a non lavora nel settore agricolo).

5	**Se Lei e/o il Suo/la Sua partner lavora nel settore agricolo: Qual è l'orientamento commerciale dell'azienda?**<br>Sono possibili più risposte.
☐	Allevamento di bestiame da pascolo
☐	Allevamento di perfezionamento (ad es. maiali o pollame)
☐	Azienda dedicata alla campicoltura
☐	Azienda agricola con colture speciali (ad. es. frutta, verdura, bacche o viti)
☐	Attività di orticoltura
☐	Azienda dedicata alla selvicoltura
☐	Altro, si prega di specificare: ………………………………………………………………..
☐	Non so
☐	Nessuna risposta

6	**<u>Se Lei e/o il Suo/la Sua partner lavora nel settore agricolo:</u> Qual è la dimensione totale approssimativa dell'azienda agricola (in ettari)?**<br>Si prega di barrare una sola risposta.
☐	Più piccola di 5 ha
☐	5-10 ha
☐	11-20 ha
☐	21-50 ha
☐	Più grande di 50 ha
☐	Non so
☐	Nessuna risposta

7	**<u>Se Lei e/o il Suo/la Sua partner lavora nel settore agricolo:</u> Quale metodo di produzione viene utilizzato nell'azienda agricola?**<br>Sono possibili più risposte.
☐	Convenzionale
☐	Produzione integrata (IP)
☐	Biologico
☐	Attualmente si sta effettuando una conversione alla produzione biologica
☐	Altro, si prega di specificare: …………………………………………………………..
☐	Non so
☐	Nessuna risposta

8	**<u>Se Lei e/o il Suo/la Sua partner lavora nel settore agricolo:</u> Chi è il/la proprietario/a dell'azienda agricola?**<br>Sono possibili più risposte.

☐ Lei stesso/a	☐ Partner/Coniuge	☐ Padre/Madre
☐ Fratello/sorella	☐ Altro membro della famiglia	☐ Persona non parente
☐ Altro, si prega di specificare:	……………………………...	☐ Non so
☐ Nessuna risposta		

9	**In generale, direbbe che la Sua salute è:**<br>Si prega di barrare una sola risposta.

☐ Eccellente ☐ Molto buona ☐ Buona ☐ Passabile ☐ Scadente

10	**In generale, direbbe che la Sua qualità di vita è:**<br>Si prega di barrare una sola risposta.

☐ Eccellente ☐ Molto buona ☐ Buona ☐ Passabile ☐ Scadente

Grazie mille per le Sue risposte! Le apprezziamo molto.

Il Suo team Swiss TPH
